# Design, Synthesis, and Antiviral Activities of New Benzotriazole-Based Derivatives

**DOI:** 10.3390/ph16030429

**Published:** 2023-03-11

**Authors:** Roberta Ibba, Paola Corona, Francesca Nonne, Paola Caria, Gabriele Serreli, Vanessa Palmas, Federico Riu, Simona Sestito, Maria Nieddu, Roberta Loddo, Giuseppina Sanna, Sandra Piras, Antonio Carta

**Affiliations:** 1 Department of Medicine, Surgery and Pharmacy, University of Sassari, Via Muroni, 23/A, 07100 Sassari, Italy; ribba@uniss.it (R.I.); pcorona@uniss.it (P.C.); federico.riu@kemi.uu.se (F.R.); marvi@uniss.it (M.N.);; 2 GSK Vaccine Institute for Global Health GSK, Via Fiorentina, 1, 53100 Siena, Italy; francesca.x.nonne@gsk.com; 3 Department of Biomedical Sciences, Section of Microbiology and Virology, University of Cagliari, Cittadella Universitaria, 09042 Monserrato, Italy; paola.caria@unica.it (P.C.); gabriele.serreli@unica.it (G.S.); vanessapalmas@hotmail.it (V.P.); rloddo@unica.it (R.L.); 4 Department of Chemistry, Biomedicinskt Centrum, BMC, Uppsala University, Box 576, 75123 Uppsala, Sweden; 5 Department of Chemical, Physical, Mathematical and Natural Sciences, University of Sassari, Via Vienna 2, 07100 Sassari, Italy; ssestito@uniss.it

**Keywords:** benzotriazoles, antiviral compounds, enterovirus, coxsackievirus B5, RNA-virus, *Picornaviridae*

## Abstract

Several human diseases are caused by enteroviruses and are currently clinically untreatable, pushing the research to identify new antivirals. A notable number of benzo[*d*][1,2,3]triazol-1(2)-yl derivatives were designed, synthesized, and in vitro evaluated for cytotoxicity and antiviral activity against a wide spectrum of RNA positive- and negative-sense viruses. Five of them (**11b**, **18e**, **41a**, **43a**, **99b**) emerged for their selective antiviral activity against Coxsackievirus B5, a human enteroviruses member among the *Picornaviridae* family. The EC_50_ values ranged between 6 and 18.5 μM. Among all derivatives, compounds **18e** and **43a** were interestingly active against CVB5 and were selected to better define the safety profile on cell monolayers by transepithelial resistance test (TEER). Results indicated compound **18e** as the hit compound to investigate the potential mechanism of action by apoptosis assay, virucidal activity test, and the time of addition assay. CVB5 is known to be cytotoxic by inducing apoptosis in infected cells; in this study, compound **18e** was proved to protect cells from viral infection. Notably, cells were mostly protected when pre-treated with derivative **18e**, which had, however, no virucidal activity. From the performed biological assays, compound **18e** turned out to be non-cytotoxic as well as cell protective against CVB5 infection, with a mechanism of action ascribable to an interaction on the early phase of infection, by hijacking the viral attachment process.

## 1. Introduction

For centuries, infectious diseases have been the leading cause of death. Unfortunately, this sad record is still upheld today, primarily in the poorest countries; however, it was sadly proved that the pandemic risk was not negligible even in the most advanced countries and viral diseases must be considered a threat that we should not underestimate.

Within the past century, multiple viruses caused pandemics across the word. It is worth mentioning the Spanish flu (1918-19), which was due to the H1N1 virus, which caused 50 million deaths [1], the Asian flu (1957), caused by the H2N2 virus [2,3], and the Hong Kong avian flu (1968), which originated from the H3N2 virus [4]. In recent history, HIV infection, causing AIDS, was arguably the most important huge viral infection, that claimed more than 36 million deaths, according to the World Health Organization (WHO) [5]. In the twenty-first century, SARS (severe acute respiratory syndrome), an atypical and particularly severe form of pneumonia, appeared in 2003 across a Chinese region and registered several cases in eight months, with a lethality rate of 10%; it was caused by SARS-CoV [6]. Later, in 2009, swine flu occurred across the American continent, caused by a H1N1 strain [7]. In 2012, another coronavirus, MERS-CoV, was identified as the cause of Middle East respiratory syndrome (MERS) [8]. Two years later, the Ebola virus was responsible for a new epidemic wave with a death rate of 50–90% [9]. Lastly, in 2019, SARS-CoV-2 circulated across the world causing the current COVID-19 pandemic [10], the effects of which we still experience. 

Even though the World Health Organization (WHO) undertook specific control and research programs for the most known viral infections (AIDS, hepatitis C, avian flu and Dengue fever) and their impact in terms of morbidity and mortality was significantly reduced compared to the past, they still remain important global public health challenges [11]. Numerous other infections caused by emerging viruses (Chikungunya, Zika) are considered very threatening by the WHO and, therefore, some of them were placed under observation, while some others were indicated as future risks for epidemics. 

Unfortunately, for most human pathogenic viruses, no vaccines or specific drugs are available, and the existing treatments are essentially symptomatic. In a few cases, targeted therapies are available (e.g., HIV, HCV), allowing access to acute and chronic treatments. 

Among the currently untreatable viruses, the enteroviruses family includes seven species: human enterovirus A–D (referred to as poliovirus, Coxsackievirus A, Coxsackievirus B, echovirus and enterovirus in the previous taxonomy) and rhinovirus A–C. These viruses can trigger numerous diseases such as common cold, non-differentiated febrile diseases, hand, foot, and mouth syndrome, epidemic pleurodynia, herpangina, poliomyelitis, aseptic meningitis, myocarditis, and various respiratory infections [12]. A benign prognosis was observed in most cases, but severe respiratory disturbances [12] or other serious complications, such as dilated cardiomyopathy or flaccid myelitis frequently occur in infants or immuno-compromised adults [13,14,15]. Due to their high diffusion in many states [16,17,18], surveillance programs for diseases caused by enteroviruses are active, confirming their significant incidence in both pediatric and adult populations. Recently, many studies also reported a peculiar correlation between enterovirus infections and celiac disease or diabetes [19,20], and therefore, they undergo specific surveillance [21,22,23]. With the worrying spread of enterovirus infections and the lack of targeted therapies and preventive vaccines, except for poliovirus and two inactivated enterovirus A71 only approved in China for the prevention of hand, foot, and mouth human disease [24], the search for therapeutic agents able to tackle or heal enteroviruses-caused infections is of great relevance and usefulness. Although, in recent years, many synthetic compounds showed good antiviral activity against enteroviruses, they failed to enter the clinic. For instance, pleconaril was rejected by the FDA due to a set of adverse effects, while vapendavir failed a phase IIb trial due to insufficient efficacy [25,26,27]. Hence, basic antiviral drug discovery programs still represent a valid contribution to the development of new antiviral compounds. Under such circumstances, the present study investigated the antiviral activities of newly synthesized benzotriazole derivatives on a panel of selected viruses.

### Rationale

Nitrogen-based heterocycles represent a precious source of scaffolds for the development of therapeutic agents in medicinal chemistry. Most of the FDA-approved drugs possess the *N*-heterocycle skeleton [28], which can be differently functionalized to obtain different compounds endowed with biological properties and, therefore, various pharmacological applications [29]. A wide number of nitrogen-containing heterocyclic derivatives were recognized to exhibit a broad range of pharmacological effects [30,31,32,33,34] and were widely reported in the literature to show intriguing biological activities for these types of compounds [35,36,37].

**Figure 1:**
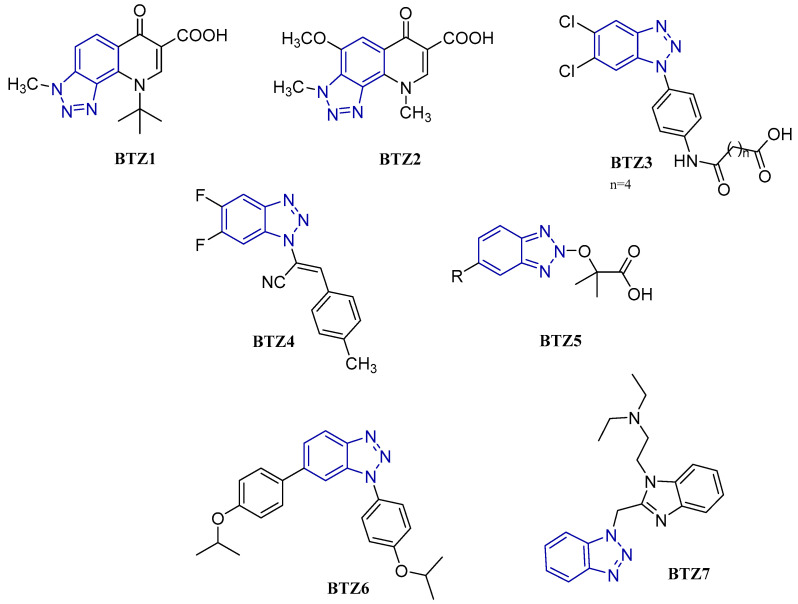
Previously reported benzotriazole-based compounds with biological activities, **BTZ1** [41], **BTZ2** [42], **BTZ3** [43], **BTZ4** [44], **BTZ5** [46], **BTZ6** [47], **BTZ7** [48].

Benzo[*d*][1,2,3]triazole (benzotriazole, BTZ) nucleus is a bicyclic scaffold frequently used in drug design for its proved endowment with a wide range of biological properties [38], including antibacterial and antiprotozoal [39], antimycotic [40], antimycobacterial [41,42], antitumor [43,44,45], and antiviral [46,47,48,49,50] activities, as reported in Figure 1.

**Figure 2:**
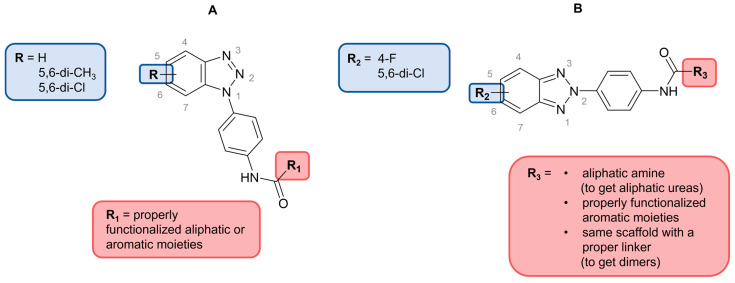
General structures of our benzotriazole-based antiviral compounds: series (**A**), active against CVB (EC_50_ values between 8–10 µM) and RSV (EC_50_ values 3–7 µM). Series (**B**), active on HTNV (EC_50_ < 5 µM), CVB (EC_50_ values between 4–8 µM) and RSV (EC_50_ values 2–3 µM) [51,52,53,54].

We previously reported different generations of BTZ-based compounds variously functionalized to exert in turn selective or wide-spectrum antiviral activity [51,52,53,54]. By screening the synthesized compounds on a panel of viruses, including representative pathogens of the *Flaviviridae*, *Picornaviridae*, *Reoviridae*, *Rhabdoviridae*, *Paramyxoviridae*, *Poxviridae*, and *Herpesviridae* families, we identified a number of hit compounds, whose general structures are active against three viral strains, as depicted in Figure 2: Coxsackievirus B (CVB) [51,53], respiratory syncytial virus (RSV) [54] and Orthohantavirus (HTNV) [52]. 

**Figure 3:**
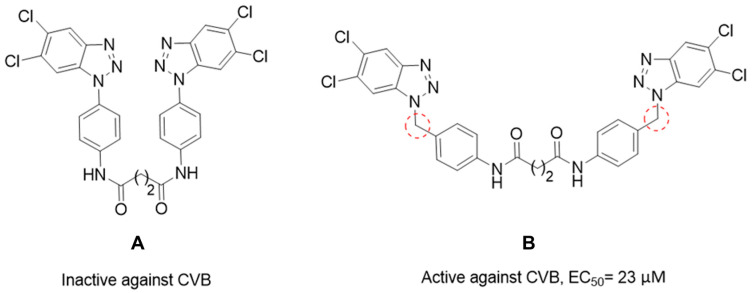
The precursor *N1*,*N4*-bis(4-(5,6-dichloro-1*H*-benzo[*d*][1,2,3]triazol-1-yl)phenyl)succinamide (**A**) and its active derivative *N1*,*N4*-bis(4-((5,6-dichloro-1*H*-benzo[*d*][1,2,3]triazol-1-yl)methyl)phenyl)succinamide (**B**).

Aiming to expand the structure-analysis knowledge on these types of molecules, in this work, we present new derivatives obtained through the manipulation of our previously developed hit compounds. Notably, we previously observed that the introduction of a methylene spacer on the not active *N1,N4*-bis(4-(5,6-dichloro-1*H*-benzo[*d*][1,2,3]triazol-1-yl)phenyl)succinimide derivative (A, Figure 3) successfully led to *N1,N4*-bis(4-((5,6-dichloro-1*H*-benzo[*d*][1,2,3]triazol-1-yl)methyl)phenyl)succinimide derivative (B, Figure 3), which was active on CVB at micromolar concentration (EC_50_ = 23 μM) [43]. 

**Figure 4:**
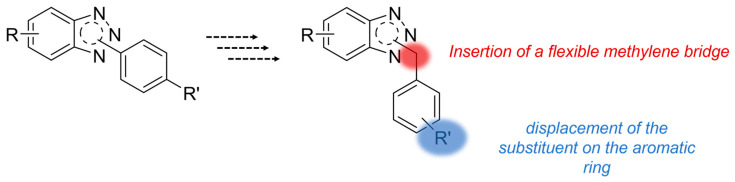
Structural modifications on our hit compounds: introduction of a methylene bridge (red) and displacement of the substituent on the benzene ring (blue).

Therefore, the structural modifications were mainly focused on a) the introduction of a methylene spacer between the benzotriazole moiety and the para-substituted benzene ring and b) the position variation of the substitution on the aromatic ring, as exemplified in Figure 4. The structural manipulations were rationally designed to allow a greater degree of molecule flexibility to evaluate the effects induced by different molecular conformations. 

## 2. Results

Three series of derivatives were obtained starting from the plain benzotriazole scaffold, substituted in position 4, or disubstituted in positions 5 and 6 with methyl groups or halogens. Then, a 3′ or 4′ benzylamine moiety was inserted on N1 or N2 of the triazole ring. The subsequent *N*-functionalization of the benzylamine portion led to aliphatic amides (**15e-18e**, **23b**, **24b**, **35a**, **36a**, **37b**, **38b**, **75b-78b**, **79c**, **80c**, **81d**, **82d**), aromatic amides (**19e**–**22e**, **25b-29b**, **39a-48a**, **49b-57b**, **83b**, **84b**, **85c-88c**, **89d-94d**), and urea-derivatives (**30b-34b**, **58a-62a**, **63b-74b**, **95a-98a**, **99b**, **100b**, **101c-103c**, **104d-107d**). 

### 2.1. Chemistry 

**Scheme 1:**
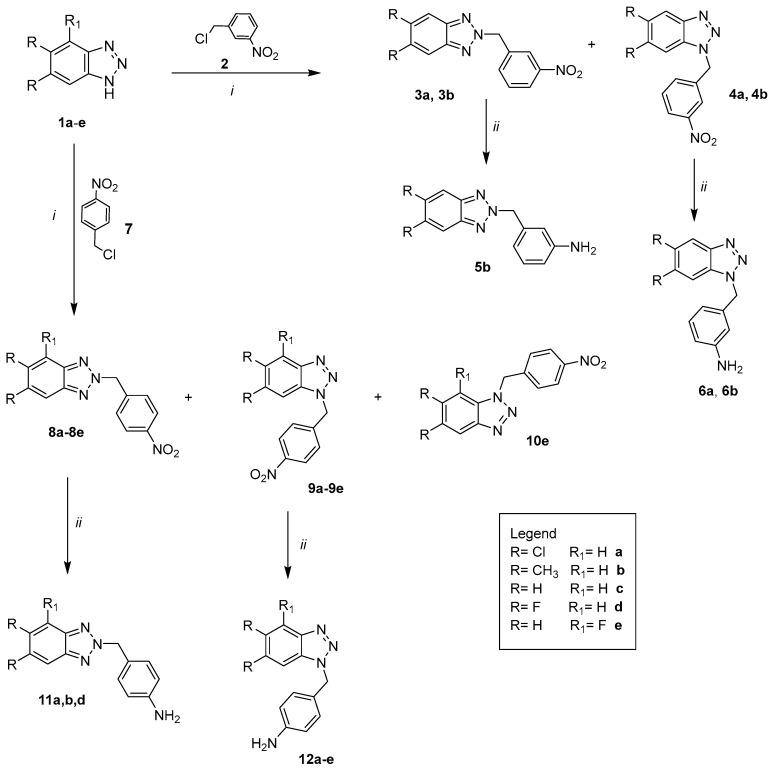
General procedure for intermediates compounds: **3a**, **b**; **4a**,**b**; **5b**; **6a**,**b**; **8a–e**; **9a–e**; **10e**, **11a**,**b**,**d**; **12a–e** Reaction conditions: *I*, from **1a**,**b**, anhydrous *N,N*-Dimethylformamide (DMF) or *N,N*-dimethylacetamide (DMA), Cs_2_CO_3_ at t = 50/70 °C; *ii*, from **1a**–**e**, ethanol, hydrated hydrazine in molar ratio 1:10, 10% palladium on activated charcoal at t = 80/90 °C or methylhydrazine in 1:10 molar ratio, at 100 °C in autoclave for 48 h.

To obtain the desired compounds, firstly, the amine intermediates were synthesized via the two-steps synthetic routes reported in Scheme 1, which yielded the uniquely aniline derivatives **5b**, **6a**,**b**, **11b**, **12a–e**. The appropriate benzotriazoles **1a–e** were condensed with 3- or 4-nitrochlorobenzyl (**2**, **7**, respectively) in a basic environment for Cs_2_CO_3_. The obtained products **3a**,**b**, **4a**,**b**, **8a**–**e**, **9a**–**e**, and **10e** were always obtained as a mixture of isomers, which were separated by flash chromatography. Each of them was subjected to a reduction reaction with methylhydrazine in autoclave in the case of chlorinated derivatives **4a**, **8a**, **9a**, while in all other cases, hydrated hydrazine with palladium on activated charcoal was used, refluxed in ethanol. Not all reductions were successful; the gained amines (**5b**, **6a**,**b**, **11a**,**b**,**d**, **12a–e**) were obtained in fair to good yields. 

**Scheme 2:**
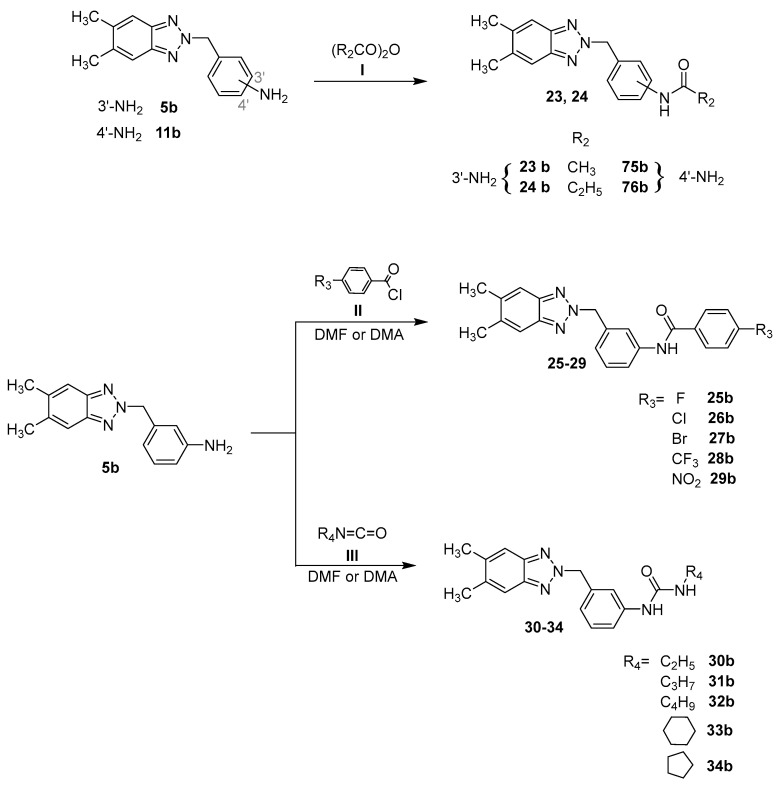
**I**. stirred at r.t.; **II**. DMA or DMF at 80 °C; **III**. DMF at 100 °C. General procedure to obtain the final compounds: amides **23b–29b**, **75b**, and **76b** and urea-derivatives **30b**–**34b** were obtained by using benzotriazol-2-ylphenylamino derivatives **5b** and **11b** as starting compounds.

**Scheme 3:**
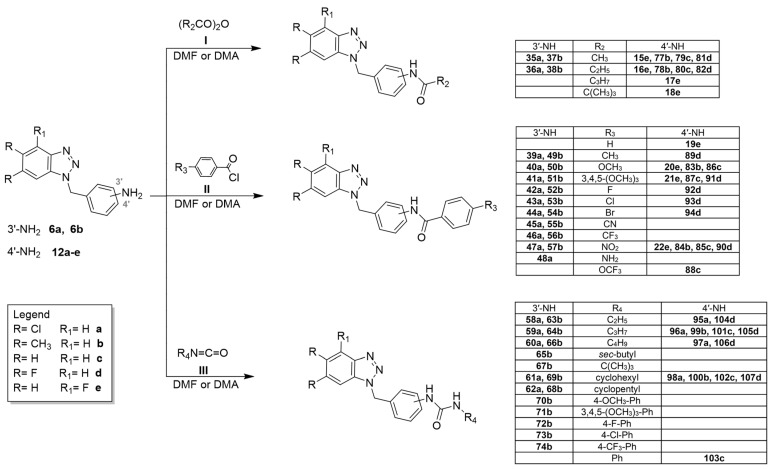
**I**. stirred at r.t.; **II**. DMA or DMF at 80 °C; **III**. DMF at 100 °C. General procedure to obtain the final compounds: Amides **15e**–**22e**, **35a**, **36a**, **37b**, **38b**, **39a**–**48a**, **49b**–**57b**, **77b**, **78b**, **79c**, **80c**, **81d**, **82d**, **83b**, **84b**, **85c**–**88c**, **89d**–**94d**. Urea-derivatives **58a**–**62a**, **63b**–**74b**, **95a**–**98a**, **99b**, **100b**, **101c**–**103c**, **104d**–**107d** were gained by using benzotriazol-1ylphenylamino derivatives **6a**, **6b**, **12a**–**e** as starting compounds.

Final derivatives **23b**–**34b**, **75b**, and **76b** were synthesized as reported in Scheme 2, while the remaining are presented in Scheme 3.

Benzotriazol-1ylphenylamino (Scheme 3) and 2-ylphenylamino (Scheme 2) intermediates (**5b**, **6a**,**b**, **11b**, **12a**–**e**) were, respectively, condensed with:

*i.*  The proper anhydride **I** (acetic anhydride, propionic anhydride, butyric anhydride and pivalic anhydride) at room temperature or for 1–72 h. The crude products were in turn obtained pure or required purification by flash chromatography;
*ii.*  The required benzoyl chloride derivatives **II** in *N*,*N*-dimethylacetamide (DMA) or *N*,*N*-dimethylformamide (DMF) at 80 °C from 3 h to 7 days. The purification of the compounds was carried out by recrystallization from ethanol or by flash chromatography;
*iii.*  The appropriate isocyanate R_2_N=C=O **III** in DMF, stirring the mixture at 100 °C from 2 to 9 days. The crude products were triturated with diethyl ether to obtain solids that were purified through recrystallization from ethanol or by flash chromatography.

### 2.2. Biology 

#### 2.2.1. Antiviral Assay

All the newly synthesized compounds were tested against representative members of several RNA and DNA viruses in cell-based assays. In detail: ssRNA- viruses: vesicular stomatitis virus (VSV) (*Rhabdoviridae*) and respiratory syncytial virus (RSV) (*Pneumoviridae*); ssRNA+ viruses: BVDV and YFV (*Flaviviridae*) and two *Picornaviridae*: human enterovirus B (coxsackievirus B5, CVB5), human enterovirus C (poliovirus type-1, Sb-1); dsRNA viruses: reovirus type-1 (Reo-1) (*Reoviridae*); DNA virus: human herpesvirus 1 (herpes simplex type-1, HSV-1) (*Herpesviridae*), and Vaccinia virus (VV) (*Poxviridae*). 2’-C-methylcytidine (NM 107), 2′-C-methyl-guanosine (NM108), ribavirin, 6-azauridine, acycloguanosine (ACG), and pleconaril were used as reference compounds. Furthermore, the cytotoxicity of all compounds was also evaluated in parallel with the antiviral activity, results are reported in Table 1.

Among the whole series of synthesized compounds, only about 15% of which showed antiviral activity, and they were the sole reported in Table 1. The non-active compounds were withheld to simplify the table readability. Most of the appealing compounds (**6a**, **11b**, **11d**, **18e**, **25b**, **41a**, **43a**, **99b**, **100b**) were found selectively active against CVB5 with EC_50_ values ranging between 6 and 52 µM, when non-inhibitory activity against the remaining virus replication was detected. Non-substituted benzotriazole-based compound **86c** was selectively active against BVDV (EC_50_ = 3 µM), while compound **21e** preferentially inhibited RSV (EC_50_ = 20 µM). 

Overall, the most promising and effective derivatives are **18e** and **43a**, whose EC_50_ values were 12.4 and 9 µM, respectively, when tested against CVB5. 

**Figure 5:**
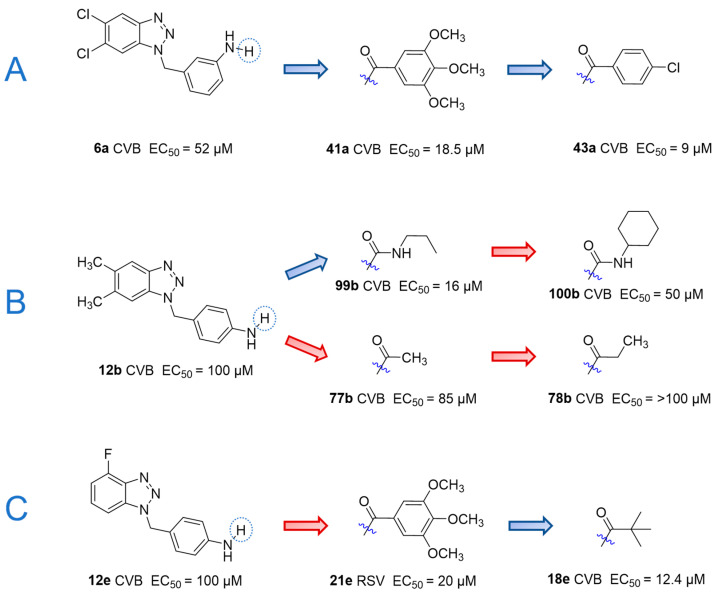
(**A**–**C**) SARs analysis from the synthetized compounds.

To further outline the structure–activity relationships (SARs), Figure 5 reported the structures of the most active compounds together with precursors and the corresponding EC_50_ values. Favorable chemical manipulations are indicated with a blue arrow, while unfavorable modifications are indicated with a red one. 

The intermediate **6a**, bearing the side chain on position C-3′, presented moderate activity towards CVB5, with an EC_50_ of 52 µM. Notably, the substitution of the amine group with 3,4,5-trimethoxybenzoyl or *p*-chlorobenzoyl groups increased the antiviral activity against the same virus, with EC_50_ values decreased to 18.5 and 9 µM for compounds **41a** and **43a**, respectively. The latter compounds bore two chlorine atoms on C-4 and C-5 of the benzotriazole scaffold (in Figure 5A). The chlorine atoms seemed to be responsible for the greater activity, since their replacement with methyl groups led to inactive derivatives **51b** and **53b** (data not shown). Aliphatic amides (**35a** and **36a**) and urea derivatives (**58a**–**62a**) obtained from chlorinated intermediate **6a** showed no antiviral activity (data not shown). Aromatic amide moiety is allegedly required for anti-CVB5 activity. Concerning the C-4′-aminobenzyl derivatives, dimethyl benzotriazole-based aliphatic-urea compounds **99b** and **100b** showed a moderate anti-CVB5 activity resulting in EC_50_ values of 16 and 50 µM, while corresponding aliphatic amides **77b** and **78b** were revealed to be inactive (in Figure 5B, data not shown). When aliphatic-urea steric hindrance was increased from **99b** to **100b**, activity decreased. 

A remarkable SAR analysis may be described when compound **21e** is compared with **18e** derivative. The former was active against RSV, while the latter inhibited the CVB5 viral replication. The two derivatives both shared the 4-F benzotriazole intermediate **12e**, but derivative **21e** carried on a trimethoxy-phenyl amide moiety, while **18e** was the simplest pivalamide (in Figure 5C). 

Among all derivatives, we selected compounds **18e** and **43a** for their interesting activity against CVB5, with comparable CC_50_ and EC_50_ values, and further experiment were performed to better define the safety profile on cell monolayers by transepithelial resistance test and deeply analyze the mechanism of anti-CVB5 action. 

#### 2.2.2. Transepithelial–Transendothelial Electrical Resistance (TEER) Test

**Figure 6:**
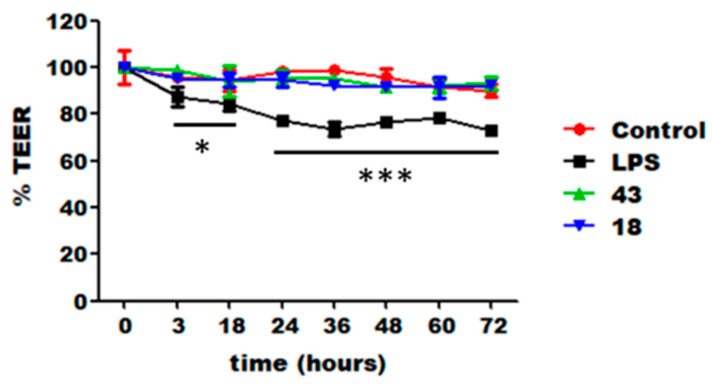
Evaluation of cell monolayer permeabilization as transepithelial electrical resistance (TEER) assay. Caco-2 cell monolayers were incubated with LPS at 1 μg/mL (black squares) as negative control, compound **18e** at 20 µM (blue triangles), compound **43a** at 20 µM (green triangles), and Control (red circles) as positive control. Statistically significant differences are expressed as follows: * = *p* < 0.05 LPS/Control; *** = *p* < 0.001 LPS vs. Control. Each value represents the mean ± SD of independent experiments (*n* = 3).

In parallel with the low cytotoxic profile shown by our derivatives against evaluated cell lines, compounds **18e** and **43a** were selected to ascertain their potential toxic effect on human cells. We tested this on differentiated intestinal Caco-2 monolayers, commonly used to simulate the gut epithelium and to evaluate changes in intestinal permeability. Cells were treated with the bacterial endotoxin lipopolysaccharide (LPS) as a negative control, and it was observed that it caused permeability imbalance and a significant alteration of the cell monolayer integrity with time (Figure 6), starting from 18 h of incubation, when the TEER value was about 80% of the level of the untreated cells. TEER values measured in monolayers treated with compounds **18e** and **43a** (20 µM), which did not significantly differ from control values throughout all the time points, showing no enhancement of cell monolayer permeability.

#### 2.2.3. Protective Effect of **18e** on Vero-76 Cell from CVB5 Infection

**Figure 7:**
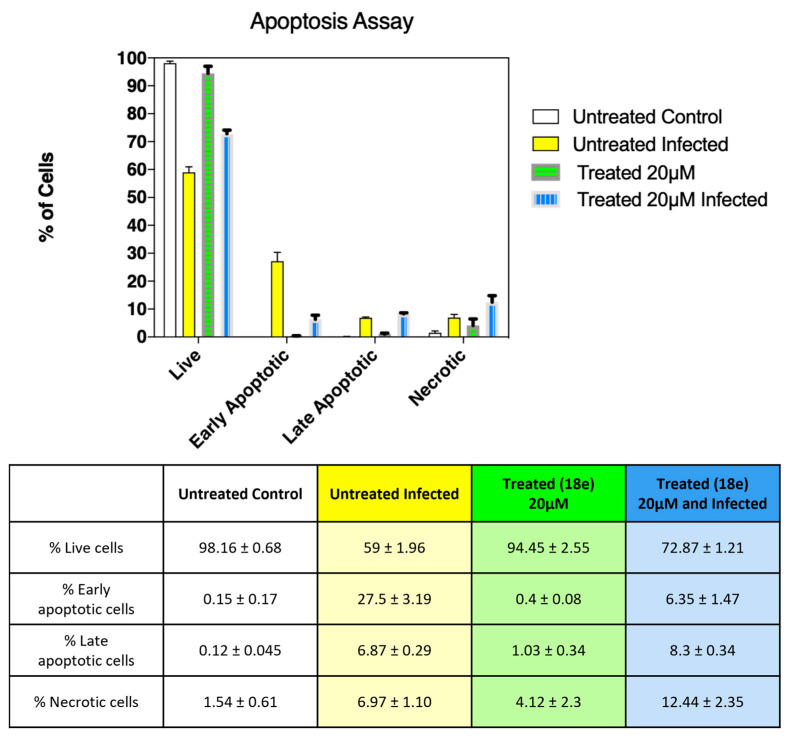
Derivative **18e** protected Vero 76 cells from apoptosis induced by CVB5 infection. The percentage of live, apoptotic, and necrotic cells were measured by flow cytometry using the PI-annexin V assay. The graph shows the percentage of live, apoptotic, and necrotic cells. Each value represents the mean ± SD of independent experiments (*n* = 3).

Among the two interesting derivatives active against CVB5 and based on the described results, we selected compound **18e** to verify whether it could hinder CVB5-induced apoptosis and preserve the monolayers viability. Vero-76 cells, growing in 12-well plates, were infected with CVB5 or left untreated. After adsorption, the cells were incubated in the absence or presence of 20 µM of compound **18e**. The cells were incubated for 48 h and then stained with Annexin-V-fluorescein and propidium iodide and, subsequently, subjected to flow cytometry analysis. Figure 7 shows that CVB5 infection induced cell death mainly by apoptosis (27.05% ± 3.19 and 6.87 ± 0.29, early and late apoptotic cells, respectively), whereas in non-infected cells treated with 20 µM of compound **18e**, a minimal number of early, late apoptotic and necrotic cells was detected, confirming the absence of cytotoxicity of the tested compound. In CVB5-infected cells, the administration of 18e at 20 µM concentration elicited a significant decrease in apoptotic cells (from 27.05% ± 3.19 in untreated infected cells versus 6.35% ± 1.47 in treated infected cells; *p* = 0.002). These results confirmed that CVB5 virus induces cell death by apoptosis and that compound **18e** protects the cells from infection. 

#### 2.2.4. Virucidal Activity 

**Figure 8:**
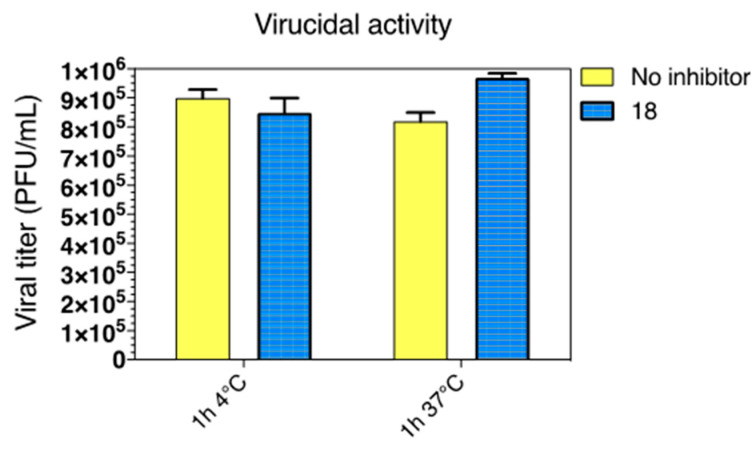
Virucidal activity (expressed as plaque-forming units (PFU/mL) of benzotriazole **18e** (20 µM) against CVB5 infectivity at either 4 °C or 37 °C for 2 h. Blue columns, viral titer for benzotriazole **18e** and viral solution; yellow columns indicate the viral titer of untreated solution.

To determine whether compound **18e** acts directly on the viral particle leading to infectivity inactivation, a virucidal assay against CVB5 virions was conducted. As shown in Figure 8, any virucidal effect was detected testing **18e** at the concentration of 20 µM at either 4 °C or 37 °C, since no difference between the titers of CVB5 treated at the two different temperatures was recorded. These results suggested that the inhibitions detected by the plaque reduction assay, reported in Table 1, may result from the interference with a CVB5 replication cycle stage.

#### 2.2.5. Time of Addition (ToA)

**Figure 9:**
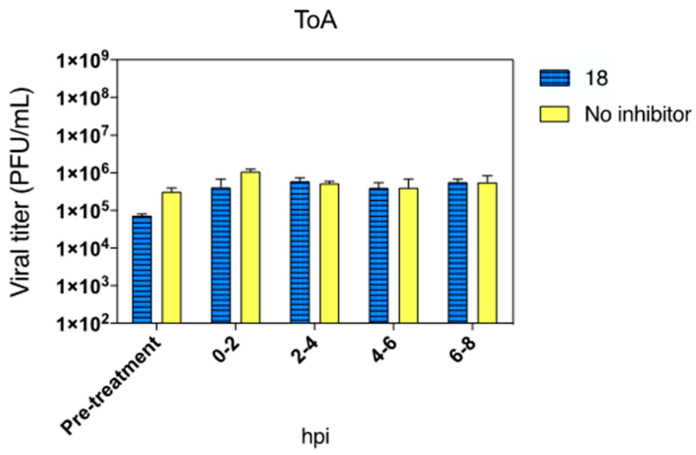
Pre-treatment and time course experiment. Vero-76 cells were pre-treated with compound **18e** (20 µM) for 2 h. After this period, the monolayers were infected with CVB5 (m.o.i. = 1). After infection period for time of addition condition, the compound **18e** (20 µM) was added at the indicated times. Viral yields were then determined by plaque assay. Dark columns, the viral yield for control cells; grey columns; viral yield for cells treated with **18e**.

In order to investigate the potential inhibitory mechanism for derivative **18e**, a cell pre-treatment and a time course assay were performed on Vero-76 monolayers. For cell pre-treatment test, Vero cells were incubated with an active and not cytotoxic concentration of compound **18e** (20 µM) for 2 h. The unbound compound was then removed, the cells infected with CVB5 for 2 h at room temperature and then washed, overlayed with new media and incubated to 37 °C for 3 days. The yield of viral particles was then determined by plaque assay and results are reported in Figure 9. Under the described experimental conditions, a decrease in viral load from 3 × 10^5^ PFU/mL (not treated control) to 7× 10^4^ (**18e**-treated) was detected. 

The antiviral effect was kept even in the time of addition assay when the compound **18e** was added during the infection period. No titer reduction was observed adding **18e** during subsequent steps of the replication cycle. These findings prompted us to hypothesize a possible inhibition during an early phase of infection, by reducing the viral attachment process to the host cell. 

## 3. Materials and Methods

### 3.1. Chemistry 

Melting points were carried out with a Köfler hot stage or Digital Electrothermal melting point apparatus. Nuclear magnetic resonance (^1^H NMR and ^13^C NMR-APT) spectra were determined in CDCl_3_ or DMSO-*d*_6_ and were recorded with a Bruker Avance III 400 NanoBay. Chemical shifts are reported in parts per million (ppm) downfield from tetramethylsilane (TMS) used as the internal standard. Splitting patterns were designated as follows: s, singlet; d, doublet; t, triplet; q, quadruplet; quin, quintet; sext, sextet; sept, septet; m, multiplet; br s, broad singlet; dd, doublet of doublets. Mass spectra (MS) were performed on combined Liquid Chromatograph-Agilent 1100 series Mass Selective Detector (MSD). Analytical thin-layer chromatography (TLC) was performed on Merck silica gel F-254 plates. Pure compounds showed a single spot in TLC. For flash chromatography, Merck silica gel 60 was used with particle sizes 0.040 and 0.063 mm (230 and 400 mesh ASTM). 

#### 3.1.1. Starting Material and Known Intermediates

Benzotriazole, 4,5-dimethylbenzotriazole, 4,5-dichloro-o-phenylendiamine, 3-nitrobenzylchloride, 4-nitrobenzylchloride, isocyanates, anhydrides, benzoyl derivatives, and inorganic compounds are commercially available. The key intermediates 3-fluorobenzene-1,2-diamine, 4-fluorobenzotriazole [41], 5,6-dichlorobenzotriazole [55], 5,6-difluorobenzotriazole [44], 3-fluorobenzene-1,2-diamine [56,57] were prepared according to the procedures described in the literature. 

#### 3.1.2. General Procedure to Obtain (5,6-R (4R))-1(2)-(3(4)-nitrobenzyl)-1(2)H-benzo[d][1,2,3]triazole (**3a**–**b**; **4a**–**b**; **8a**–**e**; **9a**–**e** and **10e**)

To a mixture of proper 5,6-dicholobenzotriazole (**1a**), 5,6-dimethylbenzotriazole (**1b**), benzotriazole (**1c**), 5,6-difluorobenzotriazole (**1d**) or 4-fluorobenzotriazole (**1e**) (6.79 mmol) in *N*,*N*- dimethylformamide anhydrous (DMF) or *N*,*N*-dimethylacetamide (DMA) (for **1e**) (40 mL), and Cs_2_CO_3_ (6,79 mmol), a solution of 3-nitro- or 4-nitrobenzylchloride (13.6 mmol) in 10 mL of DMF or DMA was added. The mixture was stirred and heated to 70 °C for 7 days (**3a**, **b**; **4a**, **b**), for 27 h (**8a**, **c**; **9a**, **c**), or for 96 h (**8b**; **9b**), 60 °C for 6 days (**8d**; **9d**), and to 50 °C for 3 h (**8e**, **9e**, **10e**). After cooling to room temperature, the solution was filtered off in vacuo to remove the Cs_2_CO_3_ and the mothers were diluted with water until complete precipitation of the products. The filtered solid was in all cases a 1:3 mixture of two isomers (**3a** and **4a**; **3b** and **4b**; **8a** and **9a**; **8b** and **9b**; **8c** and **9c**; **8d** and **9d**) or three isomers (**8e**, **9e**, **10e**). The pairs of isomers were separated and purified by flash chromatography using a mixture of petroleum ether and ethyl acetate in 8/2 or 7/3 (for **8a**, **9a** and **8e**, **9e**, **10e**) ratio. For all pairs of isomers, the first isomer to be eluted was the N-2 derivative.

#### 3.1.3. General Procedure to Obtain 4-((5,6-R-1H-benzo[d][1,2,3]triazol-1-yl)methyl)aniline **5b**, **6b**, **11b**, **11d**, **12b**, **12c**, **12e**

To a mixture of proper nitrobenzyl-benzotriazole **3b**, **4b**, **8b**, **8d**, **9b**, **9c**, and **9e** (1.77 mmol) in ethanol (20–30 mL), and hydrated hydrazine in ratio 1:10 (17.7 mmol), 10% Palladium on activated charcoal was added. The mixture was stirred and heated at 80 °C for: 15 min (**6b**, **11d**), 1h (**5b**, **11b**, **12b**, **12c**) and at 90 °C for 1.5 h (**12e**). After removal of palladium on carbon by filtration, in the case of **12b**, **12c**, the mothers obtained were concentrated into half volume. By cooling down, the resulting solid was filtered and washed twice with diethyl ether (20 mL) and, subsequently, crystallized from ethanol. In all other cases, the filtered mothers were evaporated under vacuum to obtain: the amine **5b**, **6b**, and **11b** as unit products, while the products **11d** and **12e** were purified by flash chromatography using dichloromethane/ethyl acetate 8:2 (**12e**) or petroleum ether/ ethyl acetate 7:3 (**11d**) as eluting system.

#### 3.1.4. General Procedure to Obtain 4(3)-((5,6-dichloro-1(2)H-Benzo[d][1,2,3]Triazol-1(2)-yl)methyl)aniline **6a**, **11a**, **12a**, and **12d**

Derivatives **4a**, **8a**, **9a**, and **9d**, (3.09 mmol) were solubilized in ethanol (100 mL) and reduced with methylhydrazine in 1/10 molar ratio, at 100 °C in autoclave for 48 h. The solution was concentrated under vacuum to half the volume and cooled in the freezer to separate compound **6a** as a pure solid. In the case of **11a**, **12a**, and **12d**, mother liquors were evaporated under reduced pressure and the crude products were purified by flash chromatography using an 8/2 mixture of petroleum ether/ethyl acetate. From derivative **3a**, it was not possible to obtain the corresponding amine **5a** which was debenzylated in each tested reaction condition.

#### 3.1.5. General Procedure for the Preparation of Amides **15e**–**18e**, **23b**, **24b**, **35a**, **36a**, **37b**, **38b**, **75b**–**78b**, **79c**, **80c**, **81d**, **82d**

A total of 1.2 mmol of appropriate benzylamine (**5b**, **6a**, **6b**, **11b**, **12b**, **12c**, **12d**, and **12e**) were dissolved in 3 mL of the required anhydride **I** (acetic anhydride, propionic anhydride, butyric anhydride and pivalic anhydride). The resulting mixture was stirred at room temperature for 1 h (**15e-17e**) or for 24 h (**18e**), at 50 °C for 2 h (**37b**, **38b**), at 100 °C for 1 h (**75b**, **77b**, 7**8b**, **79c**, **81d**), for 24 h (**23b**, **24b**, **35a**, **36a**, **76b**, **80c**), or for 72 h (**82d**). Then, it was cooled to room temperature and rushed ice was added. The solid obtained in each reaction was filtered under vacuum to furnish the amide derivatives. Some products were obtained pure (**16e**, **17e**, **24b**, **36a**, **37b**, **38b**, **75b-78b**, **79c**, **80c**, **81d**, **82d)**, while others required purification by flash chromatography using an appropriate eluting system as specified below: a 7:3 mixture of dichloromethane/ethyl acetate (**15e**), mixture of petroleum ether/ethyl acetate 7:3 (**18e**), 8/2 (**35a**), 6/4 (**23b**, **78b**).

#### 3.1.6. General Procedure for the Preparation of Amides **19e**–**22e**, **25b**–**29b**, **39a**–**48a**, **49b**–**57b**, **83b**, **84b**, **85c**–**88c**, **89d**–**94d**

To a solution of appropriate anilines (**5b**, **6a**, **6b**, **12b**, **12c**, **12d**, **12e**), (1.19 mmol) in *N,N*-dimethylacetamide (DMA) or *N,N*-dimethylformamide (DMF) (5–7 mL) was added by dripping an equimolar amount (**19e-22e**) or an excess of 20% in all other cases of the required benzoyl chloride derivatives **II** in 3 mL of DMF. The solution was stirred at 80 °C for different times as reported below: 3 h (**20e**, **21e**, **22e**), 24 h (**19e**, **26b**, **27b**, **29b**, **39a-46a**, **49b**, **52b**, **54b**, **57b**, **87c**, **88c**, **92d**), 48 h (**50b**, **57b**, **90d**, **93d**), 72 h (**25b**, **28b**, **53b**, **56b**, **91d**), 96 h (**84b**, **85c**), 120 h (**47a**, **86c**), 7 days (**55b**, **83b**, **89d**, **94d**). Then, it was cooled to room temperature and rushed ice was added. The solid obtained in each reaction was filtered under vacuum. The purification of the compounds was carried out by crystallization from ethanol (**27b-29b**, **39a**, **40a**, **44a-47a**, **51b**, **53b**) or by flash chromatography using as eluting system a mixture of petroleum ether/ ethyl acetate 6/4 (**20e**, **83b**, **84b**) 4/6 (**85c**, **87c**) 7/3 (**25b**, **26b**, **41a-43a**, **49b**, **50b**, **52**, **54b-57b**, **86c**, **88c**, **92d**–**94d**); dichloromethane/ethyl acetate 9/1 (**19e**, **21e**, **22e**); chloroform/methanol 9/1 (**90d**, **91d**).

#### 3.1.7. General Procedure for the Preparation of Urea-Derivatives **30b**–**34b**, **58a**–**62a**, **63b**–**74b**, **95a**–**98a**, **99b**–**100b**, **101c**–**103c**, **104d**–**107d**

To a stirred solution of corresponding amines **5b**, **6a**, **6b**, and **12a-d** (1.18 mmol) in anhydrous *N*,*N*-dimethylformamide (DMF) (7 mL), the required isocyanate **III** (ratio 1:2 + 20%) dissolved in DMF (3 mL) was added. The mixture was stirred at 100 °C for a variable time from 2 to 9 days: 2 (**67b**, **104d**), 3 (**34b**, **95a**, **105d**), 4 (**33b**, **63b**, **65b**, **66b**, **68b-74b**, **96b-98b**), 5 (**59a**, **61a**, **99b**, **100b**, **101c-103c**), 6 (**31b**, **32b**, **64b**, **106d**), 7 (**58a**, **107d**), 9 (**30b**, **60a**, **62a**). The reaction mixtures were evaporated to dryness. The crude products were triturated with diethyl ether to obtain solids that were purified through recrystallization from ethanol or, in some cases by flash chromatography using a mixture of diethyl ether and light petroleum as eluents, in ratio *v/v* of 7/3 (**99b**, **100b**), 8/2 (**101c**, **102c**), 1/1 (**103c**), or light petroleum and ethyl acetate 6/4 (**62a**, **68b**, **97a**, **104d**).

### 3.2. Biology 

#### 3.2.1. Cells and Viruses

Cell lines were purchased from American Type Culture Collection (ATCC). The absence of mycoplasma contamination was determined periodically by the Hoechst staining method. Cell lines supporting the multiplication of RNA and DNA viruses were the following: Madin Darby Bovine Kidney (MDBK) [ATCC CCL 22 (NBL-1) *Bos Taurus*]; Baby Hamster Kidney (BHK-21) [ATCC CCL 10 (C-13) *Mesocricetus auratus*]; Monkey kidney (Vero-76) [ATCC CRL 1587 *Cercopithecus Aethiops*]. Viruses were purchased from American Type Culture Collection (ATCC), with the exception of yellow gever virus (YFV). Viruses representative of positive-sense, single-stranded RNAs (ssRNA+) were: (i) *Flaviviridae*: yellow fever virus (YFV) [strain 17-D vaccine (Stamaril Pasteur J07B01)] and bovine viral diarrhea virus (BVDV) [strain NADL (ATCC VR-534)]; (ii) *Picornaviridae*: human enterovirus B [coxsackie type B5 (CVB5), strain Faulkner (ATCC VR-185)], and human enterovirus C [poliovirus type-1 (Sb-1), Sabin strain Chat (ATCC VR-1562)]. Viruses representative of negative-sense, single-stranded RNAs (ssRNA-) were: (iii) *Pneumoviridae*: human respiratory syncytial virus (RSV) strain A2 (ATCC VR-1540); (iv) *Rhabdoviridae*: vesicular stomatitis virus (VSV) [lab strain Indiana (ATCC VR 1540)]. The virus representative of double-stranded RNAs (dsRNA) was: (v) *Reoviridae* Reovirus type-1 (Reo-1) [simian virus 12, strain 3651 (ATCC VR-214)]. DNA virus representatives were: (vi) *Poxviridae*: Vaccinia virus (VV) [vaccine strain Elstree-Lister (ATCC VR-1549)]; (vii) *Herpesviridae*: human herpes 1 (HSV-1) [strain KOS (ATCC VR-1493)].

Viruses were maintained in our laboratory and propagated in appropriate cell lines. All viruses were stored in small aliquots at −80 °C until use.

#### 3.2.2. Cytotoxicity Assay

MDBK and BHK-21 cells were seeded at an initial density of 6 × 10^5^ and 1 × 10^6^ cells/mL, respectively, in 96-well plates containing minimum essential medium with Earle’s salts (MEM-E), l-glutamine, 1 mM sodium pyruvate and 25 mg/L kanamycin, supplemented with 10% horse serum (MDBK) or 10% fetal bovine serum, FBS (BHK-21). Vero-76 cells were seeded at an initial density of 5 × 10^5^ cells/mL in 96-well plates containing in Dulbecco’s modified eagle medium (D-MEM) with l-glutamine and 25 mg/L kanamycin, supplemented with 10% FBS. Cell cultures were then incubated at 37 °C in a humidified, 5% CO_2_ atmosphere, in the absence or presence of serial dilutions of test compounds. The test medium used for cytotoxic and antiviral assay contained 1% of the appropriate serum. Cell viability was determined after 72 or 96 h at 37 °C by the MTT method for MDBK, BHK-21 and Vero-76 cells [58]. 

#### 3.2.3. Transepithelial Electrical Resistance (TEER) Assay

The cytotoxicity of the compounds **18e** and **43a** was tested on intestinal epithelial cell by estimating the TEER (Transepithelial Electrical Resistance) values as a measure of cell monolayer integrity. Caco-2 cells (ECACC Salisbury, Wiltshire UK) were cultured in Dulbecco’s modified eagle’s medium (DMEM), supplemented with 10% heat-inactivated bovine serum, 100 U/mL penicillin, 2 mM l-glutamine, 1% non-essential amino acids, and 100 mg/mL streptomycin at 37 °C in a humidified atmosphere of 5% CO_2_, replacing the medium twice a week [59]. All cell culture materials were purchased from Euroclone (Milan, Italy). Caco-2 cells (5 × 10^4^ cells/well), at passage 31–40, were grown in 12 mm i.d. Transwell inserts (polycarbonate membrane, 0.4 µm pore size) (Corning Costar Corp., New York, NY, USA) and culture medium was dispensed both in the apical (0.5 mL) and in the basolateral (1.5 mL) compartment of each well. Resistance was assessed using Millicell–ERS voltohmmeter (Millicell-ERS system, Millipore, Bedford, MA, USA). After cell differentiation (>14 days), only cell monolayers in inserts with TEER values >300 Ω/cm^2^ were considered for the experiment [60]. Then, the compounds **18e** and **43a** (final concentration 30 µM) and, as a proinflammatory agent, the Gram-negative endotoxin lipopolysaccharide (LPS, 100 µg/mL) were added in the culture medium and TEER values were measured at intervals of 3, 18, 24, 36, 48, 60, and 72 h and reported as percentage of the corresponding TEER value at time zero (T = 0).

#### 3.2.4. Apoptosis Assay

To evaluate the levels of apoptosis following **18e** derivative treatment, a flow cytometric analysis, using the cell apoptosis kit Annexin V/Propidium Iodide (PI) double staining uptake (Invitrogen, Life Technologies, Italy), was used. Vero-76 cells, at the density of 3 × 10^5^ cells/mL, were seeded in 12-well plates (Corning, New York, NY, USA) with complete medium (described in the cell culture section). After CV-B5 viral adsorption, the cells were incubated in the absence or presence of different concentrations of **18e** for 48 h, until the cytopathic effect CPE of the virus control reached 70–80%. Cells were then washed once with PBS 1 X and re-suspended in 100 μL of Annexin binding buffer plus 1 μL of Annexin V and 1 μL of PI. Then, the reaction was performed in the dark for 15 minutes at room temperature. Stained cells were then analyzed by flow cytometry, measuring the fluorescence emission at 530 and 620 nm using a 488 nm excitation laser (MoFloAstrios EQ, Beckman Coulter, Pasadena, CA). Cell apoptosis was analyzed using the software Summit Version 6.3.1.1, Beckman Coulter. 

#### 3.2.5. Antiviral Assay 

Compound’s activity against YFV and Reo-1 was based on inhibition of virus-induced cytopathogenicity in BHK-21 cells acutely infected with a m.o.i. of 0.01. Compound’s activity against BVDV was based on inhibition of virus-induced cytopathogenicity in MDBK cells acutely infected with a m.o.i. of 0.01. After a 3- or 4-day incubation at 37 °C, cell viability was determined by the MTT method, as described by Pauwels et al. (1988). The compound’s activity against CVB5, Sb-1, VSV, VV, RSV A2, and HSV-1 was determined by plaque reduction assays in infected cell monolayers, as described by Sanna et al. [61].

#### 3.2.6. Virucidal Activity Assay

Benzotriazole derivatives (20 µM) were incubated with 1 × 10^5^ PFU/mL of CVB5, at either 4 or 37 °C for 1 h. The mix without test sample was employed as a control. After incubation period, samples were serially diluted in media and titers were determined on Vero-76 cells CVB5 at high dilutions, at which the derivative was not active. Titers were then determined by plaque assay in Vero-76 cells.

#### 3.2.7. Cell Pre-Treatment Assay

The monolayers of Vero-76 cell seeded in 24-well plates were incubated with 20 µM concentration of compound **18e** for 2 h. After the removal of the test compound and two washes, the cells were infected with CVB5. After the adsorption of the virus to the cells, the inoculum was removed and the monolayers were overlaid with fresh medium, incubated for 3 days at 37 °C, and then virus titers were determined by plaque assay.

#### 3.2.8. Time of Addition Assay

The monolayers of Vero-76 cells seed in 24-well tissue culture plates were infected for 1 h at room temperature with CVB5 dilutions to give final m.o.i. of 1. After adsorption, the monolayers were washed two times with D-MEM medium with l-glutamine, supplemented with 1% inactivated FBS, 1 mM sodium pyruvate and 0.025 g/L kanamycin (maintenance medium), and incubated with the same medium at 5% CO_2_ and 37 °C (time zero). Vero-76 cells CVB5 were treated with benzotriazole derivative (20 μM) or reference for 1 h during infection period (at -1 to 0) and at specific time point, 0 to 2, 2 to 4, 4 to 6, post infection. After incubation period, the monolayers were washed two times with maintenance medium and incubated with fresh medium until 12 h post infection. Then, the plates were frozen at −80 °C and the viral titers were determined by plaque assay.

#### 3.2.9. Statistical Analysis

All biological experiments were independently repeated at least three times. The data are reported as mean ± standard deviation (SD). The statistical significance (** *p* = 0.002) was performed in GraphPad Prism (San Diego, CA, USA.) 

### 3.3. Experimental 

The chemical characterization of the selected and deeply analyzed compounds **18e** and **43a** are reported below, all the other compounds’ analysis can be found in the Supplementary Material. 

*N-(4-((4-fluoro-1H-benzo[d][1,2,3]* *triazol-1-yl)methyl)phenyl)pivalamide (* ***18e*** *)*

Compound **18e** was obtained, in 19% total yield; mp: 160–163 °C; TLC (petroleum ether/ethyl acetate 7/3) R*_f_*: 0.23. ^1^H NMR (400 MHz, DMSO-*d_6_*) *δ*: 9.22 (1H, br s, NH), 7.67 (1H, d, *J* = 8.4 Hz, H-7), 7.62 (2H, d, *J* = 8.4 Hz, H-3’,5’), 7.55-7.50 (1H, m, H-6), 7.30 (2H, d, *J* = 8.4 Hz, H 2’-6’), 7.23 (1H, t, H-5), 5.94 (2H, s, CH_2_), 1.19 (9H, s, 3CH_3_). ^13^C-NMR (DMSO-*d_6_*) *δ*: 26.97 (3CH_3_), 50.87 (CH_2_), 107.23 (CH), 108.63 (CH), 119.04 (2CH), 128.14 (2CH), 128.63 (CH), 129.93 (C), 135.20 (C), 135.37 (C), 139.00 (C), 150.81 (C), 153.35 (C), 176.97 (C). LC/MS: *m*/*z* 327 [M+H]^+^.

*N-(3-((5,6-dichloro-1H-benzo[d][1,2,3]triazol-1-yl)methyl)phenyl)-4-chlorobenzamide (**43a**)*

Compound **43a** was obtained, in 15% total yield; mp: 213–214 °C; TLC (petroleum ether/ethyl acetate 7/3) R*_f_*: 0.47. ^1^H NMR (400 MHz, DMSO-*d_6_*) *δ*: 10.32 (1H, s, NH), 8.52 (1H, s, H-4), 8.4 (1H, s, H-7), 7.93 (2H, d, *J* = 8.4 Hz, H-2”,6”), 7.73 (1H, d, *J* = 7.8 Hz, H-4′), 7.68 (1H, s, H-2′), 7.59 (2H, d, *J* = 8.8 Hz, H-3”,5”), 7.36 (1H, t, *J* = 8.0 Hz, H-5′), 7.15 (1H, d, *J* = 6,8 Hz, H-6′), 6.00 (2H, s, CH_2_). ^13^C-NMR (DMSO-*d_6_*) δ:164.46 (CO), 144.25 (C), 139.42 (C), 136.42 (C), 135.93 (C), 133.40 (C), 132.08 (C), 130.79 (C), 129.59 (2CH), 129.13 (CH), 128.40 (2CH), 127.24 (C), 123.23 (CH), 120.64 (CH), 120.13 (CH), 119.33 (CH), 112.51 (CH), 51.14 (CH_2_). LC/MS *m*/*z* 415, 417 [M+H]^+^.

## 4. Conclusions

In this work, we reported the synthesis and characterization of a large series of benzotriazole-based derivatives variously functionalized on the main core and equipped with an aromatic or aliphatic chain. Compounds were assayed against a wide panel of viruses and the obtained results allowed a SARs analysis to highlight the moieties eventually endowed with antiviral activity. Most of the active compounds showed a specific activity against CVB5. Notably, derivative **18e** was found to be endowed with a considerable anti-enteroviral activity coupled with a cytotoxic profile in the high micromolar range and was selected to deepen its mechanism of action. TEER experiment was run on human epithelial cells and the results confirmed the safety of compound **18e**. When analyzed in apoptotic assay, this derivative protected cells from the CVB5-induced apoptosis. In the following time course assay, our compound displayed its utmost activity during the pre-treatment and infection period. So far, these findings prompted us to speculate on the main involvement of **18e** during the entry process of the virus and suggested our benzotriazole derivative as a potential anti-CVB5 agent worth investigating and optimizing further. 

## Figures and Tables

**Table 1:** Cytotoxicity and antiviral activity of the sole active derivatives against one or more among the following viruses: BVDV, YFV, Reo-1, CVB5, Sb-1, VV, HSV-1, VSV, and RSV. Data represent mean values of three independent determinations.

Compounds		MDBK	BVDV	BHK-21	YFV	Reo-1	Vero76	CVB5	Sb-1	VV	HSV-1	VSV	RSV
		^a^ CC_50_	^b^ EC_50_	^c^ CC_50_	^d^ EC_50_		^e^ CC_50_	^f^ EC_50_					
Amines	**6a**	>100	>100	57	<57	<57	>100	52 ± 3.6	>100	62.5 ± 16.2	>100	>100	>100
	**11b**	>100	>100	>100	>100	>100	>100	**6**	>100	>100	>100	>100	>100
	**11d**	>100	>100	>100	>100	>100	>100	7.6 ± 1.9	>100	>100	>100	>100	>100
Amides	**18e**	>100	>100	>100	>100	>100	>100/9	12.4 ± 0.6	>100	>100	**52 ± 9.2**	>100	>9
	**21e**	72	<72	60	<60	<60	>100	>100	>100	>100	>100	>100	**20 ± 2**
	**25b**	>100	>100	>100	>100	>100	>100	47 ± 2.2	>100	>100	>100	>100	>100
	**41a**	>100	>100	>100/7	>100	>7	>100/10.5	18.5 ± 2.1	>100	>100	>100	>100	>10.5
	**43a**	>100	>100	>100	>100	>100	>100/9.2	9 ± 1.6	>100	>100	>100	>100	>9.2
	**75b**	>100	>100	>100	>100	>100	>100	80	>100	>100	>100	>100	>100
	**77b**	>100	>100	>100	>100	>100	>100	85	>100	>100	>100	>100	>100
	**79c**	44	**>44**	86	>86	>86	72/9	>72	>72	>72	>72	>72	>9
	**86c**	87	**3**	95	<95	<95	85	<85	<85	<85	<85	<85	<85
Ureas	**32b**	>100	<100	>100	>100	>100	>100	>100	**85**	>100	>100	>100	>100
	**99b**	>100	>100	>100	>100	>100	>100	16	>100	>100	>100	>100	>100
	**100b**	>100	>100	>100	>100	>100	>100	50	>100	<100	>100	>100	>100
Ref cmps	NM107			>100		6 ± 2.1							
	NM108	>100	1.5	>100	1.0								
	Ribav	35/52	12/18										
	6az-ur			>100	46 ± 2.5		9.3 ± 1.6						1.1 ± 0.4
	ACG	s					>100				2.4 ± 0.6		
	Plec						77 ± 6.8	0.005 ± 0.002	2 ± 0.62				

^a^ Compound concentration (μM) required to reduce the viability of mock infected MDBK cells by 50%, as determined by the MTT method. ^b^ Compound concentration (μM) required to achieve 50% protection of MDBK cells from the BVDV-induced cytopathogenicity, as determined by the MTT method. ^c^ Compound concentration (μM) required to reduce the viability of mock-infected BHK-21 monolayers by 50%, as determined by the MTT method. ^d^ Compound concentration (μM) required to achieve 50% protection of BHK-21 cells from the YFV and Reo-induced cytopathogenicity, as determined by the MTT method. ^e^ Compound concentration (μM) required to reduce the viability of mock-infected VERO-76 monolayers by 50%. ^f^ Compound concentration (μM) required to reduce the plaque number of the CVB5, Sb-1, VV, HSV-1, VSV and RSV virus by 50% in VERO-76 monolayers. Data represent mean values + SD for three independent determinations. Where not reported, variation among duplicate samples was less than 15%.

## Data Availability

Data is contained within the article.

## Supplementary Materials

The following supporting information can be downloaded at: https://www.mdpi.com/article/10.3390/ph16030429/s1, Figure S1. Cytotoxicity and broad-spectrum antiviral activity of selected benzotriazole derivatives (**86c**, **21e**, **43a**, **41a**, **11d**, **18e**), Experimental characterization of the all the synthetized compounds, Figures S2–12. ^1^H NMR and ^13^C NMR spectra of selected benzotriazole derivatives (**86c**, **21e**, **43a**, **41a**, **11d**, **18e**).
