# Supplementary material for: Design, Synthesis, and Antiviral Activities of New Benzotriazole-Based Derivatives
Source: Pharmaceuticals (Basel). 2023 Mar 11;16(3):429. doi: 10.3390/ph16030429 (PMC10054465; doi:10.3390/ph16030429)
Supplement: Supplementary file 1 [file pharmaceuticals-16-00429-s001.zip › pharmaceuticals-2243712-supplementary.pdf]

pharmaceuticals

Supporting Material

Design, Synthesis, and Antiviral Activities of New Benzotriazole-Based Derivatives

Roberta Ibba [1,†], Paola Corona [1,†], Francesca Nonne [2], Paola Caria [3], Gabriele Serreli [3], Vanessa Palmas [3], Federico Riu [1,4], Simona Sestito [5], Maria Nieddu [1], Roberta Loddo [3], Giuseppina Sanna [3,*], Sandra Piras [1,*] and Antonio Carta [1]

[1] Department of Medicine, Surgery and Pharmacy, University of Sassari, Via Muroni, 23/A, 07100 Sassari, Italy; ribba@uniss.it (R.I.); pcorona@uniss.it (P.C.); marvi@uniss.it (M.N.); federico.riu@kemi.uu.se (F.R.)
[2] GSK Vaccine Institute for Global Health GSK, Via Fiorentina, 1, 53100 Siena, Italy; francesca.x.nonne@gsk.com
[3] Department of Biomedical Sciences, Section of Microbiology and Virology, University of Cagliari, Cittadella Universitaria, 09042, Monserrato, Italy; paola.caria@unica.it (P.C.); gabriele.serreli@unica.it (G.S.); vanessapalmas@hotmail.it (V.P.); rloddo@unica.it (R.L.)
[4] Department of Chemistry, Biomedicinskt Centrum, BMC, Uppsala University, Box 576, 75123 Uppsala, Sweden
[5] Department of Chemical, Physical, Mathematical and Natural Sciences, University of Sassari, Via Vienna 2, 07100 Sassari, Italy; ssestito@uniss.it
* Correspondence: piras@uniss.it (S.P.); g.sanna@unica.it (G.S.)
† These authors equally contributed to this work.

Supporting Material

Dose-response curves for 6 most active compounds

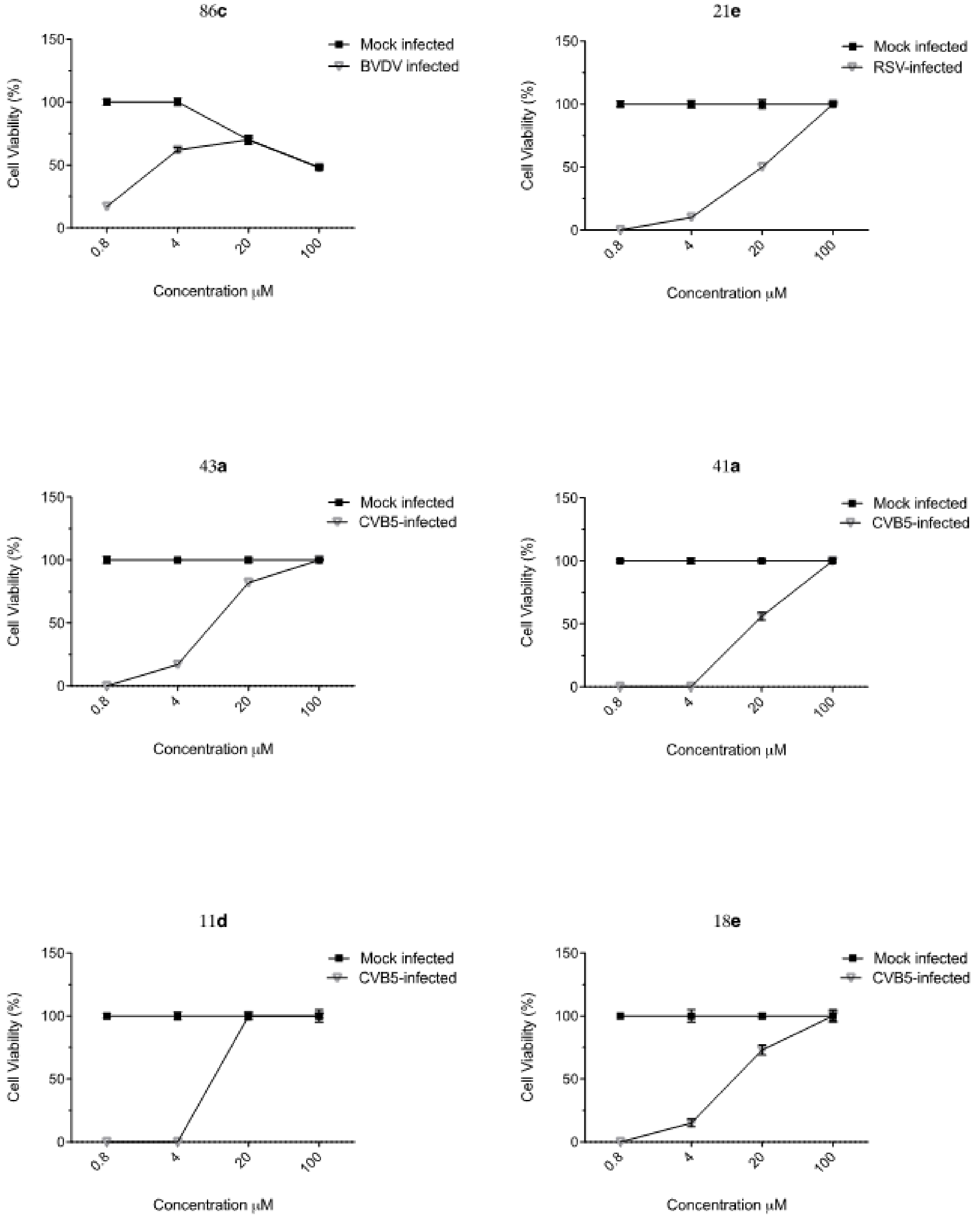

Figure S1. Cytotoxicity and broad-spectrum antiviral activity of selected benzotriazole derivatives (**86c, 21e, 43a, 41a, 11d, 18e**). The viability of mock-infected cells was estimated by MTT assay, three days after-infection. The number of live cells was ex-pressed as a percentage of mock-infected, untreated control cells. Data are expressed as means ± SD of at least two independent measurements.

Experimental – chemical characterization

5,6-dichloro-2-(3-nitrobenzyl)-2H-benzo [d][1,2,3]triazole *(3a).*

Compound **3a** was obtained in 5% total yield; m.p. : 237-239 °C; TLC (petroleum ether/ethyl acetate = 7/3) R_f: 0.58; ^{1}H-NMR (400 MHz, DMSO-d_6) δ: 8.43 (2H, s, H-4,7), 8.34 (1H, s, H-2′), 8.24 (1H, d, *J* = 8.0 Hz, H-4′), 7.85 (1H, d, *J* = 7.6 Hz, H-6′), 7.70 (1H, t, *J* = 8.0 Hz, H-5′), 6.22 (2H, s, CH_2). ^{13}C-NMR (DMSO-*d6*) δ: 142.9 (2C), 136.6 (2C), 136.5 (CH), 130.1 (CH), 129.9 (2C), 123.5 (CH), 123.4 (CH), 119.5 (2CH), 58.7 (CH_2). LC/MS *m/z* 323, 325 $[M+H]^+$.

5,6-dimethyl-2-(3-nitrobenzyl)-2H-benzo [d][1,2,3]triazole (3b)

Compound **3b** was obtained in 12% total yield; m.p.: 145-148 °C TLC (petroleum ether/ethyl acetate = 8,5/1,5) R_f : 0.40; ^{1}H-NMR (400 MHz, DMSO-d_6) δ: 10.59 (1H, s, NH), 8.35 (2H, d, *J* = 8.4 Hz, H-3″,5″), 8.15 (2H, d, *J* = 8.4 Hz, H-2″,6″), 7.77 (1H, d, *J* = 7.6 Hz, H-4′), 7.32 (1H, s, H-2′), 7.66 (2H, s, H-4,7), 7.15 (1H, d, *J* = 7.6 Hz, H-6′), 5.91 (2H, s, CH_2), 2.36 (6H, s, CH_3). ^{13}C-NMR (DMSO-d_6) δ: 163.9 (C=O), 149.1 (C), 143.3 (C), 140.4 (C), 139.0 (C), 136.6 (3C), 136.2 (C), 129.2 (C), 129.2 (2CH), 129.0 (CH), 123.9 (CH), 123.5 (CH), 120.2 (CH), 119.8 (CH), 116.2 (CH), 59.1 (CH_2), 20.3 (CH_3); LC/MS *m/z* 283 $[M+H]^+$.

5,6-dichloro-1-(3-nitrobenzyl)-1H-benzo [d][1,2,3]triazole (4a)

Compound **4a** was obtained in 44% total yield; m.p.: 156-157 °C TLC (petroleum ether/ethyl acetate = 7/3) R_f: 0.46;^{1}H-NMR (400 MHz, DMSO-d_6) δ: 8.50 (1H, s, H-4), 8.48 (1H, s, H-7), 8.30 (1H, s, H-2′), 8.17 (1H, d, *J* = 8.0 Hz, H-4′), 7.78 (1H, d, *J* = 7.8 Hz, H-6′), 7.65 (1H, t, *J* = 8.0 Hz, H-5′), 6.14 (2H, s, CH_2).^{13}C-NMR (DMSO-d_6) δ: 143.1 (2C), 136.6 (2C), 135.7 (CH), 130.9 (CH), 130.1 (2C), 123.5 (CH), 123.4 (CH), 119.5 (2CH), 58.1 (CH_2). LC/MS *m/z* 323, 325 $[M+H]^+$.

5,6-dimethyl-1-(3-nitrobenzyl)- 1H-benzo [d][1,2,3]triazole (4b)

Compound **4b** was obtained in 40% total yield; m.p.: 130-132 °C; TLC (petroleum ether/ethyl acetate = 8,5/1,5) R_f: 0,16; ^{1}H-NMR (400 MHz, DMSO-d_6) δ: 8.15-8.24 (2H, m, H-4′,6′), 7.80 (1H, 1, H-2′), 7.76-7.67 (3H, m, H-4,7,5′) 6.10 (2H, s, CH_2), 2.34 (6H, s, $2CH_3$).^{13}C-NMR (DMSO-d_6) δ: 149.0 (C), 144.5 (C), 138.3 (C), 137.8 (C), 134.6 (CH), 134.1 (C), 131.7 (C), 130.4 (CH), 122.6 (CH), 122.3 (CH), 116.2 (CH), 109.5 (CH), 49.6 (CH_2), 20.4 (CH_3), 19.8 (CH_3). LC/MS *m/z* 283 $[M+H]^+$.

3-((5,6-dimethyl-2H-benzo[d][1,2,3]triazol-2-yl)methyl)aniline (5b)

Compound **5b** was obtained in 93% total yield; m.p.: 140-144 °C; TLC (petroleum ether/ethyl acetate 6/4) R_f : 0.56; ^{1}H-NMR (400 MHz, DMSO-d_6) δ: 7.63 (2H, s, H-4,7), 6.95 (3H, t, H-5′), 6.45 (3H, m, H-2′,4′,6′), 5.68 (2H, s, CH_2), 5.13 (2H, s, NH_2), 2.34 (6H, s, $2CH_3$); ^{13}C-NMR (DMSO-d_6) δ: 148.9 (C), 143.2 (2C), 136.4 (2C), 136.1 (C), 129.0 (CH), 115.8 (CH), 115.2 (CH), 113.6 (CH), 113.0 (CH), 112.6 (CH), 59.5 (CH_2), 23.2 (CH_3), 20.3 (CH_3). LC/MS *m/z* 253 $[M+H]^+$.

3-((5,6-dichloro-1H-benzo[d][1,2,3]triazol-1-yl)methyl)aniline (6a)

Compound **6a** was obtained in 55% total yield; m.p.161-162 °C; TLC: (petroleum ether/ethyl acetate 7/3) R_f : 0.29; ^{1}H-NMR (400 MHz, DMSO-d_6) δ: 8.49 (1H, s, H-4), 8.30 (1H, s, H-7), 6.97 (1H, t, *J* = 7.5 Hz, H-5′), 6.45-6.43 (2H, m, H-4′,6′), 6.40 (1H, s, H-2′), 5.80 (2H, s, CH_2), 5.15 (2H, s, NH_2); ^{13}C-NMR (DMSO-d_6): 149.1 (C), 144.2 (C), 135.9 (C), 132.1 (C), 130.6 (C), 129.3 (CH), 127.1 (C), 120.6 (CH), 114.9 (CH), 113.7 (CH), 112.5 (CH), 112.5 (CH), 51.1 (CH_2). LC/MS: *m/z* 293, 295 $[M+H]^+$.

3-((5,6-dimethyl-1H-benzo[d][1,2,3]triazol-1-yl)methyl)aniline (6b)

Compound **6b** was obtained in 90% total yield; m.p. 146-149 °C; TLC (petroleum ether/ethyl acetate 6/4) R_f : 0.32; ^{1}H-NMR (400 MHz, DMSO-d_6) δ: 7.78 (1H, s, H-4), 7.52 (1H, s, H-7), 6.96 (3H, t, *J* = 8,0 Hz, H-5′), 6.43 (2H, m, H-4′,6′), 6.37 (1H, s, H-2′), 5.71 (2H, s, CH_2), 5.10 (2H, s, NH_2), 2.34 (6H, s, $2CH_3$);^{13}C-NMR (DMSO-d_6) δ: 149.0 (C), 144.5 (C), 137.2 (C), 136.6 (C), 133.4 (C), 131.8 (C), 129.1 (C), 118.1 (C), 114.7 (C), 113.4 (C), 112.3 (C), 109.8 (C), 51.0 (C), 20.4 (C), 19.8 (C). LC/MS *m/z* 253 $[M+H]^+$.

5,6-dichloro-2-(4-nitrobenzyl)-2H-benzo [d][1,2,3]triazole (8a)

Compound **8a** was obtained in 14% total yield; m.p.: 146-148 °C; TLC (petroleum ether/ethyl acetate = 7,5/2,5) R_f : 0.58; ^{1}HNMR (400 MHz, $CDCl_3$) δ: 8.35-8.23 (4H, m, H-3′,5′, H-7, H-4), 7.63 (2H, d, *J* = 8.4, H-2′,6′), 5.51 (2H, s, CH_2);^{13}C-NMR (DMSO-d_6) δ: 146.8 (2C), 145.9 (C), 141.2 (C), 133.4 (2C), 129.9 (2CH), 127.2 (2CH), 123.8 (2CH), 61.3 (CH_2). LC/MS *m/z* 323, 325 $[M+H]^+$.

5,6-dimethyl-2-(4-nitrobenzyl)- 2H-benzo [d][1,2,3]triazole (8b)

Compound **8b** was obtained in 37% total yield; m.p.: 140-142 °C; TLC (petroleum ether/ethyl acetate 8 / 2) R_f : 0.46; ^{1}H-NMR (400 MHz, DMSO-d_6) δ: 8.28 (2H, d, *J* = 8.6 Hz, H-3′,5′), 7.76 (2H, d, *J* = 8.6 Hz, H-2′,6′), 7.67 (1H, s, H-4,7), 5.55 (2H, s, CH_2), 2.35 (6H, s, $2CH_3$). ^{13}C-NMR (DMSO-d_6) δ: 147.2 (C), 146.9 (2C), 141.5 (C), 138.2 (2C), 127.9 (2CH), 125.5 (2CH), 116.5 (2CH), 55.4 (CH_2), 18.8 ($2CH_3$); LC/MS *m/z* 283 $[M+H]^+$.

2-(4-nitrobenzyl)-2H-benzo [d][1,2,3]triazole (8c)

Compound **8c** was obtained in 20% total yield; m.p.: 118-122 °C;(petroleum ether/ethyl acetate 8/2) R_f : 0.39;^{1}H-NMR (200 MHz, DMSO-d_6) δ: 8.37 (2H, d, *J* = 8.6 Hz, H-3′,5′), 8.29-8.26 (4H, m, arom), 7.79 (2H, d, *J* = 8.8 Hz, H-2′,6′),

5.56 (2H, s, CH_2); ^{13}C-NMR (DMSO-*d6*) δ: 145.6 (C), 144.9 (2C), 142.3 (C), 128.9 (2CH), 126.3 (2CH), 123.8 (2CH), 118.5 (2CH), 58.2 (CH_2). LC/MS *m/z* 255 [M+H]+.

5,6-difluoro-2-(4-nitrobenzyl)- 1H-benzo [d][1,2,3]triazole (8d)

Compound **8d** was obtained in 27% total yield; m.p.: 179-180 °C; TLC (petroleum ether/ethyl acetate 7/3) R_f : 0.63; ^{1}H-NMR (200 MHz, DMSO-*d6*) δ: 8.23 (2H, d, *J* = 7.8 Hz, H- 3′, 5′), 8.10-7.81 (2H, m, H-4, 7), 7.56 (2H, d, *J* = 7.8 Hz, H- 2′, 6′), 6.16 (2H, s, CH_2).^{13}C-NMR (DMSO-*d6*) δ: 149.5 (2C), 145.5 (C), 145.4 (C), 132.8 (C), 126.2 (C), 129.8 (2CH), 117.1 (2 CH), 115.1 (2CH), 54.7 (CH_2). LC/MS *m/z* : 291 [M+H]+.

4-fluoro-2-(4-nitrobenzyl)- 2H-benzo [d][1,2,3]triazole (8e)

Compound **8e** was obtained in 3% total yield; mp: 136-139 °C; TLC (petroleum ether/ethyl acetate 8:2) R_f : 0.33; ^{1}H-NMR (400 MHz, DMSO-*d6*) δ: 8.25 (2H, d, *J* = 8.4 Hz, H-3',5'), 7.80 (1H, d, *J* = 8.4 Hz, H-7), 7.64 (2H, d, *J* = 8.4 Hz, H-2',6'), 7.48-7.43 (1H, m, H-6), 7.29 (1H, t, H-5), 6.23 (2H, s, CH_2).^{13}C-NMR (DMSO-*d6*) δ:151.4 (C), 147.4 (C), 146.6 (C), 142.1 (C), 134.9 (C), 129.6 (2CH), 127.1 (CH), 123.9 (2CH), 114.5 (CH), 110.2 (CH), 58.8 (CH_2). LC/MS: *m/z* 273 [M+H]+.

5,6-dichloro-1-(4-nitrobenzyl)-1H-benzo [d][1,2,3]triazole (9a)

Compound **9a** was obtained in 32% total yield; m.p.:158-160 °C; TLC (petroleum ether/ethyl acetate 7.5/2.5) R_f : 0.42; ^{1}H-NMR (400 MHz, $CDCl_3$) δ: 8.21 (3H, d, *J* = 7.8 Hz, H-3′,5′, H-4), 7.88 (1H, s, H-7), 7.51 (2H, d, *J* = 8.6 Hz, H-2′,6′), 6.03 (2H, s, CH_2). ^{13}C-NMR (DMSO-*d6*) δ: 145.9 (C), 145.3 (C), 142.5 (C), 133.8 (2C), 132.6 (C), 128.6 (2CH), 126.5 (2CH), 124.3 (2CH), 52.0 (CH_2).LC/MS *m/z* 323, 325 [M+H]+.

5,6-dimethyl-1-(4-nitrobenzyl)-1H-benzo [d][1,2,3]triazole (9b)

Compound **9b** was obtained in 45% total yield; m.p.143-145 °C; TLC (petroleum ether/ethyl acetate 8/2) R_f : 0.17; ^{1}H-NMR (200 MHz, DMSO-*d6*) δ: 8.28 (2H, d, *J* = 8.8 Hz, H-3′,5′), 7.82 (1H, s, H-4) 7.62 (1H, s, H-7), 7.48 (2H, d, *J* = 8.8 Hz, H-2′,6′), 6.10 (2H, s, CH_2), 2.35 (6H, s, $2CH_3$); ^{13}C-NMR (DMSO-*d6*) δ: 145.6 (C), 144.9 (C), 142.2 (C), 132.9 (C), 131.9 (2C), 127.9 (2CH), 118.5 (2CH), 116.5 (2CH), 52.1 (CH_2), 18.8 ($2CH_3$). LC/MS *m/z* 283 [M+H]+.

1-(4-nitrobenzyl)- 1H-benzo [d][1,2,3]triazole (9c) [34]

Compound **9c** was obtained in 44% total yield; m.p.: 126-128 °C; TLC (petroleum ether/ethyl acetate 8/2) R_f : 0.13; ^{1}H-NMR (200 MHz, DMSO-*d6*) δ: 8.22 (2H, d, *J* = 8.4 Hz, H-3′, 5′), 8.09 (1H, d, *J* = 8.2 Hz, H-4), 7.87 (1H, d, *J* = 8.2 Hz, H-7), 7.70–7.60 (1H, m, H arom), 7.54 (2H, d, *J* = 8.4 Hz, H-2′,6′), 7.47–7.43 (1H, m, H arom), 6.19 (2H, s, CH_2). ^{13}C-NMR (DMSO-*d6*) δ: 145.6 (C), 144.9 (C), 142.3 (C), 132.9 (C), 128.9 (2CH), 126.3 (2CH), 123.8 (2CH), 118.5 (CH), 110.5 (CH), 52.2 (CH_2). LC/MS *m/z* 283 [M+H]+.

5,6-difluoro-1-(4-nitrobenzyl)-1H-benzo [d][1,2,3]triazole (9d)

Compound **9d** was obtained in 73 % total yield; m.p.: 139-140 °C; TLC (petroleum ether/ethyl acetate 7/3) R_f : 0.37; ^{1}H-NMR (200 MHz, DMSO-*d6*)δ: 8.30-8.14 (4H, m, H-4, 7, 3′, 5′), 7.56 (2H, d, *J* = 8.8 Hz, H-2′, 6′), 6.14 (2H, s, CH_2).^{13}C-NMR (DMSO-*d6*) δ: 149.5 (2C), 145.5 (C), 144.9 (C), 142.3 (C), 132.8 (C), 128.9 (2CH), 123.8 (2CH), 117.1 (2 CH), 54.7 (CH_2). LC/MS *m/z*: 291 [M+H]+.

4-fluoro-1-(4-nitrobenzyl)- 1H-benzo [d][1,2,3]triazole (9e)

Compound **9e** was obtained in 15% total yield; mp: 161-164 °C; TLC (petroleum ether/ethyl acetate 8:2): R_f : 0.10. ^{1}H NMR (400 MHz, DMSO-*d6*):δ: 8.22 (2H, d, *J* = 8.8 Hz, H-3',5'), 7.73 (1H, d, *J* = 8.4 Hz, H-7), 7.56 (2H, d, *J* = 8.8 Hz, H-2',6' + 1H, H-6), 7,26 (1H, t, H-5), 6,21 (2H, s, CH_2).^{13}C-NMR (DMSO-*d6*): 152.3 (C), 147.3 (C), 142.9 (C), 135.8 (C), 135.2 (C), 128.9 (CH), 128.9 (2CH), 123.8 (2CH), 108.8 (CH), 107.2 (CH), 50.3 (CH_2). LC/MS: *m/z* 273 [M+H]+.

7-fluoro-1-(4-nitrobenzyl)-1H-benzo[d][1,2,3]triazole(10e)

Compound **10e** was obtained in 13% total yield; mp: 155-158 °C; TLC (petroleum ether/ethyl acetate 8:2) R_f : 0.22. ^{1}H NMR (400 MHz, DMSO-*d6*) δ: 8.22 (2H, d, *J* = 8 Hz, H-3',5'), 7.95 (1H, d, *J* = 7.2 Hz, H-4), 7.48 (2H, d, *J* = 8 Hz, H-2',6'), 7.43-7.39 (2H, m, H-5,6), 6.20 (2H, s, CH_2).^{13}C-NMR (DMSO-*d6*) δ: 148.8 (C), 148.3 (C), 146.5 (C), 143.3 (C), 128.4 (2CH), 125.2 (CH), 124.0 (2CH), 122.5 (C), 115.8 (CH), 112.6 (CH), 52.0 (CH_2). LC/MS: *m/z* 273 [M+H]+.

4-((5,6-dichloro-2H-benzo[d][1,2,3]triazol-2-yl)methyl)aniline (11a)

Compound **11a** was obtained in 10% total yield; m.p. 247-250 °C; TLC: (petroleum ether/ethyl acetate 7/3) R_f : 0.49; ^{1}H-NMR (400 MHz, DMSO-*d6*) δ: 8.03 (2H, s, H-4,7), 7.10 (2H, d, *J* = 8.2 Hz, H-2′,6′), 6.48 (2H, d, *J* = 8.4 Hz, H-3′,5′), 5.74 (2H, s, CH_2), 5.26 (2H, s, NH_2).^{13}C-NMR (DMSO-*d6*) δ: 145.9 (2C), 145.2 (C), 133.8 (2C), 129.0 (2CH), 126.3 (2CH), 125.0 (C), 114.9 (2CH), 58.7 (CH_2). LC/MS: *m/z* 293, 295 [M+H]+.

4-((5,6-dimethyl-2H-benzo[d][1,2,3]triazol-2-yl)methyl)aniline (11b)

Compound **11b** was obtained in 27% total yield; m.p. 169-171°C; TLC (petroleum ether/ethyl acetate 6/4); R_f : 0.45; ^{1}H-NMR (400 MHz, DMSO-*d6*) δ: 7.62 (1H, s, H-4,7), 7.07 (2H, d, *J* = 8.2 Hz, H-2′,6′), 6.50 (2H, d, *J* = 8.4 Hz, H-3′,5′), 5.63 (2H, s, CH_2), 5.16 (2H, s, NH_2), 2.34 (6H, s, $2CH_3$). ^{13}C-NMR (DMSO-*d6*) δ: 145.5 (2C), 145.4 (C), 136.8 (2C), 129.8 (2CH), 124.4 (C), 124.1 (2CH), 115.1 (2CH), 61.6 (CH_2), 18.8 ($2CH_3$). LC/MS *m/z* 253 [M+H]+.

4-((5,6-difluoro-2H-benzo[d][1,2,3]triazol-2-yl)methyl)aniline (11d)

Compound **11d** was obtained in 20% total yield; m.p.: 149-150 °C; TLC (petroleum ether/ethyl acetate 7/3); R$_f$: 0.34; ^{1}H-NMR (400 MHz, DMSO-*d$_6$*) *δ*: 8.04 (2H, t, H-4,7), 7.11 (2H, d, *J* = 8.00 Hz, H- 2′,6′), 6.52 (2H, d, *J* = 8.4 Hz, H-3′,5′), 5.71 (2H, s, CH_2), 5.19 (2H, s, NH_2). ^{13}C-NMR (DMSO-*d$_6$*) *δ*: 149.5 (2C), 145.5 (2C), 145.4 (C), 129.8 (2CH),124.4 (C), 117.1 (2CH), 115.1 (2CH), 61.6 (CH_2). LC/MS: *m/z* 261 [M+H]$^+$.

4-((5,6-dichloro-1H-benzo[d][1,2,3]triazol-1-yl)methyl)aniline (12a)

Compound **12a** was obtained in 30% total yield; m.p.: 203-206 °C; TLC (petroleum ether/ethyl acetate 7/3); R$_f$: 0.29; ^{1}H-NMR (400 MHz, $CDCl_3$) *δ*: 8.45 (1H, s, H-4), 8.32 (1H, s, H-7), 7.11 (2H, d, *J* = 8.4 Hz, H-2′,6′), 6.51 (2H, d, *J* = 8.4 Hz, H-3′,5′), 5.75 (2H, s, CH_2), 5.13 (2H, s, NH_2), ^{13}C-NMR (DMSO-*d$_6$*) *δ*: 145.7 (C),144.9 (C), 133.8 (2C), 133.0 (C), 129.5 (2CH), 126.6 (2CH), 125.9 (C), 115.4 (2CH), 52.3 (CH_2). LC/MS: *m/z* 293, 295 [M+H]$^+$.

4-((5,6-dimethyl-1H-benzo[d][1,2,3]triazol-1-yl)methyl)aniline (12b)

Compound **12b** was obtained in 88% total yield; m.p.: 184-186 °C; TLC (petroleum ether/ethyl acetate 6/4); R$_f$: 0.17; ^{1}H-NMR (200 MHz, DMSO-*d$_6$*) *δ*: 7.76 (1H, s, H-4), 7.57 (1H, s, H-7), 7.06 (2H, d, *J* = 8.6 Hz, H-2′,6′), 6.48 (2H, d, *J* = 8.6 Hz, H-3′,5′), 5.65 (2H, s, CH_2), 5.13 (2H, s, NH_2), 2.36 (6H, s, 2CH_3). ^{13}C-NMR (DMSO-*d$_6$*) *δ*: 149.2 (C), 145.5 (C), 132.5 (2C), 132.2 (C), 128.8 (C), 128.2 (2CH), 124.2 (2CH), 114.3 (2CH), 52.3 (CH_2), 18.8 (2CH_3). LC/MS *m/z* 253 [M+H]$^+$.

4-((1H-benzo[d][1,2,3]triazol-1-yl)methyl) aniline (12c)

Compound **12c** was obtained in 78% total yield; m.p.: 151-153 °C; TLC (petroleum ether/ethyl acetate 4/6); R$_f$: 0.47; ^{1}H-NMR (400 MHz, DMSO-*d$_6$*) *δ*: 8.03 (1H, d, *J* = 8.4 Hz, H-4), 7.81 (1H, d, *J* = 8.4 Hz, H-7), 7.50 (1H, t, H-5), 7.41 (1H, t, H-6) 7.07 (2H, d, *J* = 8.2 Hz, H-3′,5′), 6.49 (2H, d, *J* = 8.2 Hz, H-2′,6′), 5.74 (2H, s, CH_2), 5.15 (2H, s, NH_2). ^{13}C-NMR (DMSO-d$_6$) *δ*: 149.2 (C), 145.5 (C), 132.4 (C), 132.8 (C), 128.7 (2CH), 126.6 (2CH), 119.7 (CH), 114.6 (2CH), 110.3 (CH), 52.3 (CH_2). LC/MS *m/z* 225 [M+H]$^+$.

4-((5,6-difluoro-1H-benzo[d][1,2,3]Triazol-1-yl)methyl)aniline (12d)

Compound **12d** was obtained in 70% total yield; m.p.: 116-117 °C; TLC (petroleum ether/ethyl acetate 8/2); R$_f$: 0.14; ^{1}H-NMR (400 MHz, $CDCl_3$) *δ*: 8.20 (H, t, H-4), 8.07 (H, t, H-7), 7.11 (2H, d, *J* = 8.6 Hz, H-2′, 6′), 6.51 (2H, d, *J* = 8.6 Hz, H-2′, 5′), 5.71 (2H, s, CH_2), 5.18 (2H, s, NH_2). ^{13}C-NMR (DMSO-*d$_6$*) *δ*: 149.5 (2C), 145.5 (C), 145.4 (C), 132.8 (C), 129.8 (2CH), 126.2 (C), 117.1 (2CH), 115.1 (2CH), 54.7 (CH_2). LC/MS: *m/z* [M+H]$^+$.

4-((4-fluoro-1H-benzo[d][1,2,3]triazol-1-yl)methyl)aniline (12e)

Compound **12e** was obtained in 43%. total yield; mp 164-167 °C; TLC (dichloromethane/ethyl acetate 9.5/0.5) R$_f$: 0.17; ^{1}H NMR (400 MHz, $CDCl_3$): *δ* 7.65 (1H, d, *J* = 7.0 Hz, H-7), 7.53-7.48 (1H, m, H-6), 7.20 (1H, t, H-5), 7.09 (2H, d, *J* = 8.0 Hz , H-3',5'), 6.51 (2H , d, *J* = 8.0 Hz , H-2'-6'), 5.77 (2H, s, CH_2), 5.16 (2H, s, NH_2).^{13}C-NMR (400 MHz, $CDCl_3$) *δ*: 152.1 (C), 148.6 (C), 135.3 (C), 135.2 (C), 129.1 (2CH), 128.3 (CH), 122.0 (C), 113.8 (2CH), 108.4 (CH), 107.4 (CH), 51.5 (CH_2). LC/MS: *m/z* 243 [M+H]$^+$.

N-(4-((4-fluoro-1H-benzo[d][1,2,3]triazol-1-yl)methyl)phenyl)acetamide (15e)

Compound **15e** was obtained in 11% total yield; m.p.: 233-234 °C; TLC (dichloromethane/ethyl acetate 7:3): R$_f$: 0.37. ^{1}H NMR (400 MHz, DMSO-*d$_6$*) *δ*: 9.97 (1H, br s, NH), 7.68 (1H, d, *J* = 8.4 Hz , H-7), 7.54 (2H, d, *J* = 7.6 Hz, H-3′,5′ and H-6), 7.30 (2H, d, *J* = 7.6 Hz, H-2′,6′), 7.23 (1H, t, H-5), 5.94 (2H, s, CH_2), 2.01 (3H, s, CH_3).^{13}C-NMR (DMSO-*d$_6$*) *δ*: 168.5 (C), 152.2 (C), 139.0 (C), 135.5 (C), 129.7 (C), 128.6 (2CH), 124.0 (CH), 119.2 (2CH), 110.6 (CH), 108.5 (CH), 107.3 (CH), 50.9 (CH_2),23.8 (CH_3). LC/MS: *m/z* 285 [M+H]$^+$.

N-(4-((4-fluoro-1H-benzo[d][1,2,3]triazol-1-yl)methyl)phenyl)propionamide(16e)

Compound **16e** was obtained, in 78% total yield; mp: 244-246 °C. TLC (petroleum ether /ethyl acetate 7:3): R$_f$: 0.19. ^{1}H NMR (400 MHz, DMSO-*d$_6$*) *δ*: 9.89 (1H, br s,), 7.91 (1H, d, *J* = 8.8 Hz, H-7), 7.68 (2H, d, *J* = 8.4 Hz, H-3',4'), 7.57-7.50 (1H, m, H-6), 7.37 (2H, d, *J* = 8.4Hz, H-2',6'), 7.25-7.20 (1H, m, H-5), 5.93 (2H, s, CH_2), 2.29 (2H, d, CH_2), 1.033 (3H, t, CH_3).^{13}C-NMR (DMSO-*d$_6$*) *δ*: 172.0 (C), 152.2 (C), 139.3 (C), 135.5 (C), 135.3 (C), 129.6 (C), 129.1 (3CH), 119.2 (2CH), 108.5 (CH), 107.4 (CH), 51.3 (CH_2), 29.4 (CH_2), 9.6 (CH_3). LC/MS: *m/z* 299 [M+H]$^+$.

N-(4-((4-fluoro-1H-benzo[d][1,2,3]triazol-1-yl)methyl)phenyl)butyramide (17e)

Compound **17e** was obtained, in 40% total yield; mp: 215-216 °C; TLC (petroleum ether /ethyl acetate 7:3): R$_f$: 0.16. ^{1}H NMR (400 MHz, DMSO-*d$_6$*) *δ*: 9.91 (1H, br s, NH), 7.66 (1H, d, *J* = 8.4 Hz, H-5), 7.56-7.49 (3H, m, H-3',5'and 7'), 7.30 (2H, d, *J* = 8.4 Hz, H- 2',6'), 7.22 (1H, dd, *J* = 3.2 e *J* = 7,6 Hz, H-6), 5.92 (2H, s, CH_2), 2.24 (2H, t, CH_2), 1.57 (2H, d, CH_2), 0.87 (3H, t, CH_3).^{13}C-NMR (DMSO-*d$_6$*) *δ*: 171.5 (C), 152.1 (C), 139.1 (C), 139.0 (C), 135.4 (C), 129.7 (C), 128.6 (CH), 128.5 (2CH), 119.3 (2CH), 108.6 (CH), 107.3 (CH), 51.0 (CH_2), 18.5 (CH_2), 13.5 (CH_3). LC/MS: *m/z* 313 [M+H]$^+$.

N-(4-((4-fluoro-1H-benzo[d][1,2,3]triazol-1-yl)methyl)phenyl)benzamide (19e)

Compound **19e** was obtained, in 89% total yield; mp: 214-217 °C; TLC (dichloromethane/ethyl acetate 9:1): R$_f$: 0.59. ^{1}H NMR (400 MHz, DMSO-*d$_6$*) *δ*: 10.29 (1H, br s, NH), 7.96 (2H, d, *J* = 7.2 Hz, H-2'',6''), 7.77 (2H, d, *J* = 8 Hz, H-3',5'),7.71 (1H, d, *J* = 8.4 Hz, H-7), 7.58-7.50 (4H, m, H-3'',4'',5'',6), 7.37 (2H, d, *J* = 8 Hz, H-2',6'), 7.23 (1H, t, H-5), 5.98

(2H, s, CH_2). ^{13}C-NMR (DMSO-d_6) δ: 166.0 (C), 152.7 (C), 139.6 (C), 136.0 (C), 135.8 (C), 135.2 (C), 132.1 (CH), 130.9 (C), 129.0 (CH), 128.9 (3CH), 128.1 (2CH), 121.0 (2CH), 109.1 (CH), 108.9 (CH), 17.8 (CH), 51.5 (CH_2). LC/MS *m/z* 347 $[M+H]^+$.

N-(4-((4-fluoro-1H-benzo[d][1,2,3]triazol-1-yl)methyl)phenyl)-4-methoxybenzamide (20e)

Compound **20e** was obtained, in 94% total yield; mp: 266-268 °C; TLC (petroleum ether/ethyl acetate 6:4): R_f: 0.29.^{1}H NMR (400 MHz, DMSO-d_6) δ: 10.13 (1H, br s, NH), 7.93 (2H, d, *J* = 8.8 Hz, H-2",6"), 7.75 (2H, d, *J* = 8 Hz, H-3',5'), 7.71 (1H, d, *J* = 8.4 Hz, H-7), 7.57-7.53 (1H, m, H-6), 7.36 (2H, d, *J* = 8.4 Hz, H-2',6'), 7.23 (1H, t, H-5), 7.05 (2H, d, *J* = 8.8 Hz, H-3",5"), 5.97 (2H, s, CH_2), 3.83 (3H, s, OCH_3). ^{13}C-NMR (DMSO-d_6) δ: 164.9 (C), 161.9 (C), 152.2 (C), 139.3 (C), 135.6 (C), 135.2 (C), 130.1 (C), 129.6 (2CH), 128.5 (CH), 128.3 (2CH), 126.7 (C), 120.5 (2CH), 113.6 (2CH), 108.5 (CH), 107.4 (CH), 55.4 (CH_3), 51.1 (CH_2). LC/MS *m/z* 377 $[M+H]^+$.

N-(4-((4-fluoro-1H-benzo[d][1,2,3]triazol-1-yl)methyl)phenyl)-3,4,5-trimethoxybenzamide (21e)

Compound **21e** was obtained, in 94% total yield; mp: 177-179 °C; TLC (dichloromethane/ethyl acetate 9:1): R_f: 0.22.^{1}H NMR (400 MHz, DMSO-d_6) δ:10.16 (1H, br s, NH), 7.73-7.70 (3H, d, *J* = 8.8 Hz, H-7-2",6"), 7.57-7.52 (1H, m, H-6), 7.36 (2H, d, *J* = 8.4 Hz, H-3',5'), 7.25-7.21 (3H, m, H-5, 2',6'), 5.99 (2H, s, CH_2), 3.86 (6H, s, 3,5-OCH_3), 3.73 (3H, s, OCH_3). ^{13}C-NMR (DMSO-d_6) δ: 164.9 (C), 152.6 (C), 152.2 (C), 140.3 (C), 139.0 (C), 135.4 (C), 135.4 (C), 135.2 (C), 130.5 (C), 129.8 (C), 128.6 (CH), 128.5 (2CH), 120.8 (2CH), 108.6 (CH), 107.3 (CH), 105.3 (2CH), 60.1 (CH_3), 56.1 (2CH_3), 51.0 (CH_2). LC/MS *m/z* 437 $[M+H]^+$.

N-(4-((4-fluoro-1H-benzo[d][1,2,3]triazol-1-yl)methyl)phenyl)-4-nitrobenzamide (22e)

Compound **22e** was obtained, in 91% total yield; mp: 272-274 °C; TLC (dichloromethane/ethyl acetate 9:1): R_f: 0.33.^{1}H NMR (400 MHz, DMSO-d_6) δ: 10.61 (1H, br s, NH), 8.36 (2H, d, *J* = 8.8 Hz, H-2",6"), 8.16 (2H, d, *J* = 8.8 Hz, H-3",5"), 7.76 (2H, d, *J* = 8.4 Hz, H-3',5'), 7.71 (1H, d, *J* = 8.4 Hz, H-7), 7.57-7.52 (1H, m, H-6), 7.39 (2H, d, *J* = 8.4 Hz, H-2',6'), 7.24 (1H, t, H-5), 6.00 (2H, s, CH_2). ^{13}C-NMR (DMSO-d_6) δ: 163.9 (C), 152.2 (C), 149.2 (C), 140.4 (C), 138.6 (C), 135.5 (C), 135.2 (C), 131.0 (C), 129.2 (3CH), 128.5 (2CH), 123.5 (2CH), 120.7 (2CH), 108.6 (CH), 107.4 (CH), 51.0 (CH_2). LC/MS *m/z* 392 $[M+H]^+$.

N-(3-((5,6-dimethyl-2H-benzo[d][1,2,3]triazol-2-yl)methyl)phenyl) acetamide (23b)

Compound **23b** was obtained, in 43% total yield; mp: 184-187 °C; TLC (petroleum ether /ethyl acetate 6:4): R_f: 0.13. ^{1}H NMR (400 MHz, DMSO-d_6) δ: 9.94 (1H, s, NH), 7.65 (2H, s, H-4,7), 7.55 (1H, d, *J* = 8Hz, H-4′), 7.49 (1H, s, H-2′), 7.27 (1H, t, *J* = 7.8 Hz, H-5′), 7.02 (1H, d, *J* = 7.6 Hz, H-6′), 5.85 (2H, s, CH_2), 2.35 (6H, s, 2CH_3), 1.99 (3H, s, CO-CH_3).^{13}C-NMR (DMSO-d_6) δ: 168.3 (CO), 145.5 (2C), 138.4 (C), 136.8 (2C), 136.4 (C), 128.8 (CH), 122.6 (CH), 118.6 (CH), 118.2 (CH), 116.2 (2CH), 59.2 (CH_2), 23.9 (CO-CH_3), 20.3 (2CH_3). LC/MS *m/z* 295 $[M+H]^+$, 317 $[M+Na]^+$.

N-(3-((5,6-dimethyl-2H-benzo[d][1,2,3]triazol-2-yl)methyl)phenyl)propionamide (24b)

Compound **24b** was obtained, in 80% total yield; mp: 178-182 °C; TLC (petroleum ether /ethyl acetate 6:4): R_f: 0.40. ^{1}H NMR (400 MHz, DMSO-d_6) δ: 9.94 (1H, s, NH), 7.84 (2H, s, H-4,7), 7.65 (1H, d, *J* = 7.5 Hz, H-2″, 6″), 7.55 (1H, s, H-2′), 7.30 (1H, t, *J* = 7.30 Hz, H-5′), 7.02 (1H, d, *J* = 7.0 Hz, H-4′), 6.85 (2H. d, *J* = 6.80 Hz, H-3″,5″), 6.57 (1H, d *J* = 6.6 Hz, H-6′), 5.86 (2H, s, CH_2), 2.34 (6H, s, 2CH_3).^{13}C-NMR (DMSO-d_6) δ: 165.2 (CO), 154.9 (C), 140.2 (C), 137.3 (C), 136.3 (C), 133.4 (C), 131.7 (C), 128.8 (CH), 128.8 (2CH), 124.4 (C), 123.5 (CH), 119.8 (CH), 119.3 (CH), 118.1 (CH), 112.5 (CH), 111.2 (CH), 109.7 (CH), 50.9 (CH_2), 20.4 (CH_3), 19.8 (CH_3). LC/MS *m/z* 309 $[M+H]^+$, 332 $[M+Na]^+$.

N-(3-((5,6-dimethyl-2H-benzo[d][1,2,3]triazol-2-yl)methyl)phenyl)-4-fluorobenzamide (25b)

Compound **25b** was obtained, in 27% total yield; mp: 211-212 °C; TLC (petroleum ether/ethyl acetate = 7/3) R_f: 0.10. ^{1}H NMR (400 MHz, DMSO-d_6) δ: 10.16 (1H, s, NH), 8.23 (1H, s, H.2′), 7.99 (1H, t, *J* = 8 Hz, H-5′), 7.65(2H, s, H-4,7), 7.55 (2H, d, *J* = 7.5 Hz, H-2″,6″), 7.32 (3H, m, H-4′, 3″, 5″), 7.05 (1H, d, *J* = 7.6 Hz, H-6′), 5.86 (2H, s. CH_2), 2.35 (6H, s, 2CH_3). ^{13}C-NMR (DMSO-d_6) δ: 166.2 (CO), 143.3 (2C), 138.5 (C), 136.5 (3C), 136.3 (C), 132.1 (CH), 132.0 (CH), 129.4 (CH), 123.3 (CH), 119.5 (CH), 118.8 (CH), 116.2 (2CH), 115.7 (CH), 115.5 (CH), 59.0 (CH_2), 20.30 (CH_3). LC/MS 300 *m/z* 375 $[M+H]^+$, 397 $[M+Na]^+$.

N-(3-((5,6-dimethyl-2H-benzo[d][1,2,3]triazol-2-yl)methyl)phenyl)-4-chlorobenzamide (26b)

Compound **26b** was obtained, in 56% total yield; mp: 246-247 °C; TLC (petroleum ether/ethyl acetate = 7/3) R_f: 0.50. ^{1}H NMR (400 MHz, DMSO-d_6) δ: 10.33 (1H, s, NH), 7.95 (2H, d, *J* = 7.95 Hz, H-2″,6″), 7.75 (2H, m, H-2′,4′), 7.66 (2H, s, H-4,7), 7.59 (2H, d, *J* = 7.6 Hz, H-3″,5″), 7.35 (1H, t, *J* = 7.35 Hz, H-5′), 7.12 (1H, d, *J* = 7.1, H-6′), 5.89 (2H, s, CH_2), 2.36 (6H, s, 2CH_3).^{13}C-NMR (DMSO-d_6) δ: 164.4 (CO), 143.3 (C), 139.3 (C), 137.7 (2C), 136.5 (C), 136.1 (C), 131.0 (2CH), 129.6 (2C), 129.6 (CH), 129.2 (2CH), 123.4 (CH), 119.9 (CH), 118.6 (CH), 116.2 (2CH), 59.1 (CH_2), 20.3 (2CH_3). LC/MS 300 *m/z* 391 $[M+H]^+$, 413 $[M+Na]^+$.

N-(3-((5,6-dimethyl-2H-benzo[d][1,2,3]triazol-2-yl)methyl)phenyl)-4-bromobenzamide (27b)

Compound **27b** was obtained, in 17% total yield; mp: 267-268 °C; TLC (petroleum ether/ethyl acetate = 7/3) R_f: 0.85. ^{1}H NMR (400 MHz, DMSO-d_6) δ: 10.34 (1H, s, NH), 7.87 (2H, s, H-4,7), 7.74 (4H, s, H-2″,3″,5″,6″), 7.66 (2H, s,

H-2′,4′), 7.35 (1H, s, H-5′), 7.13 (1H, s, H-6′), 5.89 (2H, s, CH_2), 2.35 (6H, s, CH_3). ^{13}C-NMR (DMSO-d_6) δ: 164.5 (CO), 143.3 (C), 139.3 (C), 136.8 (2C), 136.4 (C), 136.1 (C), 133.8 (C), 131.3 (2CH), 129.8 (2CH), 128.9 (CH), 125.4 (C), 123.6 (CH), 120.1 (CH), 119.7 (CH), 116.2 (CH), 59.2 (CH_2), 20.3 ($2CH_3$). LC/MS 300 *m/z* 436 [M+H]$^+$, 458 [M+Na]$^+$.

N-(3-((5,6-dimethyl-2H-benzo[d][1,2,3]triazol-2-yl)methyl)phenyl)-4-trifluoromethyl benzamide (28b)

Compound **28b** was obtained, in 18% total yield; mp: 237-239 °C; TLC (petroleum ether/ethyl acetate = 6/4) R_f: 0.64. ^{1}H NMR (400 MHz, DMSO-d_6) δ: 10.49 (1H, s, NH), 8.11 (2H, d, *J* = 8.1 Hz, H-2″,6″), 7.89 (2H, d, *J* = 7.95 Hz, H-3″,5″), 7.79-7.74 (3H, m, H-2′,4′), 7.66 (2H, s, H-4,7), 7.37 (1H, t, *J* = 7.3 Hz, H-5′), 7.14 (1H, d, *J* = 7.1 Hz, H-6′), 5.90 (2H, s, CH_2), 2.35 (6H, s, $2CH_3$). ^{13}C-NMR (DMSO-d_6) δ: 164.4 (CO), 143.3 (2C), 139.1 (2C), 138.6 (C), 136.6 (2C), 136.1 (C), 131.5 (CF_3), 129.0 (CH), 128.6 (2CH), 125.3 (2CH), 123.7 (CH), 122.5 (CH), 120.2 (CH), 116.2 (2CH), 59.2 (CH_2), 20.3 (CH_3). LC/MS *m/z* 425 [M+H]$^+$.

N-(3-((5,6-dimethyl-2H-benzo[d][1,2,3]triazol-2-yl)methyl)phenyl)-4-nitrobenzamide (29b)

Compound **29b** was obtained, in 43% total yield; mp: 184-187 °C; TLC (petroleum ether/ethyl acetate = 6/4) R_f: 0.13. ^{1}H NMR (400 MHz, DMSO-d_6) δ: 10.59 (1H, s, NH), 8.35 (2H, d, *J* = 8.4 Hz, H-2″,6″). 8.15 (2H, d, *J* = 8.4Hz, H-3″,5″), 7.77 (1H, d, *J* = 7.6Hz), H-4′), 7.73 (1H, s, H-2′), 7.66 (2H, s, H-4,7), 7.38 (1H, t, *J* = 7.8 Hz, H-5′), 5.91 (2H, s, CH_2), 2.36 (6H, s, $2CH_3$). ^{13}C-NMR (DMSO-d_6) δ: 163.9 (CO), 149.1 (C), 143.3 (C), 140.4 (C), 139.0 (C), 136.6 (3C), 136.2 (C), 129.2 (2CH),129.0 (CH), 123.9 (CH), 123.5 (2CH), 120.2 (CH), 119.8 (CH), 116.2 (2CH), 59.1 (CH_3), 20.3 (CH_3). LC/MS *m/z* 402 [M+H]$^+$, 424 [M+Na]$^+$.

1-(3-((5,6-dimethyl-2H-benzo[d][1,2,3]triazol-2-yl)methyl)phenyl)-3-ethylurea (30b)

Compound **30b** was obtained, in 32% total yield; mp: 218-219 °C; TLC (petroleum ether/ethyl acetate = 6/4) R_f: 0.15. ^{1}H NMR (400 MHz, DMSO-d_6) δ: 8.45 (1H, s, NH), 7.65 (2H, s, H-4,7), 7.34 (1H, d, *J* = 8.4 Hz, H-4′), 7.32 (1H, s, H-2′), 7.18 (1H, t, H-5′), 6.86 (1H, d, *J* = 7.2 Hz, H-6′), 6.02 (1H, t, NH), 5.81 (2H, s, CH_2), 3.07 (2H, q, CH_2), 2.35 (6H, s, $2CH_3$), 1.02 (3H, t, CH_3). ^{13}C-NMR (DMSO-d_6) δ: 154.9 (CO), 143.3 (C), 140.9 (C), 136.5 (2C), 136.0 (2C), 128.9 (CH), 120.5 (CH), 117.2 (CH), 116.8 (CH), 116.2 (2CH), 59.3 (CH_2), 33.9 (CH_2), 20.3 ($2CH_3$), 15.4 (CH_3). LC/MS *m/z*: 324 [M+H]$^+$.

1-(3-((5,6-dimethyl-2H-benzo[d][1,2,3]triazol-2-yl)methyl)phenyl)-3-propylurea (31b)

Compound **31b** was obtained, in 48% total yield; mp: 204-206 °C; TLC (petroleum ether/ethyl acetate = 6/4) R_f: 0.21. ^{1}H NMR (400 MHz, DMSO-d_6) δ: 8.44 (1H, s, NH), 7.65 (2H, s, H-4,7), 7.34 (1H, d, *J* = 8.0 Hz, H-4′), 7.31 (1H, s, H-2′), 7.18 (1H, t, H-5′), 6.86 (1H, d, *J* = 7.6 Hz, H-6′), 6.06 (1H, t, NH), 5.81 (2H, s, CH_2), 3.00 (2H, q, CH_2-NH), 2.35 (6H, s, $2CH_3$), 1.41-1.35 (2H, m, CH_2), 0.87-0.82 (3H, m, CH_3). ^{13}C-NMR (DMSO-d_6) δ: 155.0 (CO), 143.3 (C), 140.9 (C), 136.5 (2C), 136.0 (2C), 128.9 (CH), 120.5 (CH), 117.5 (CH), 117.2 (CH), 116.2 (2CH), 59.3 (CH_2), 40.8 (CH_2), 22.9 (CH_2), 20.3 ($2CH_3$), 11.3 (CH_3). LC/MS *m/z*: 338 [M+H]$^+$.

1-butyl-3-(3-((5,6-dimethyl-2H-benzo[d][1,2,3]triazol-2-yl)methyl)phenyl)urea (32b)

Compound **32b** was obtained, in 10% total yield; mp: 288-289 °C; TLC (petroleum ether/ethyl acetate = 6/4) R_f: 0.35. ^{1}H NMR (400 MHz, DMSO-d_6) δ: 8.65 (1H, s, NH), 7.66 (2H, s, H-4,7), 7.37 (1H, s, H-2′), 7.38 (1H, d, *J* = 8 Hz H-4′), 7.20 (1H, t, H-5′), 6.94 (1H, d, *J* = 7.6 Hz H-6′,), 6.04 (1H, t, NH), 5.81 (2H, s, CH_2), 3.04 (2H, q, CH_2), 1.38-1.27 (6H, m, $2CH_2$), 0.87 (3H, t, CH_3). ^{13}C-NMR(DMSO-d_6) δ: 155.0 (CO), 143.3 (C), 140.9 (C), 139.9 (C), 136.5 (C), 136.2 (2C), 129.1 (CH), 121.5 (CH), 117.8 (CH), 117.5 (CH), 116.2 (2CH), 59.2 ($2CH_2$), 31.8 (CH_2), 20.3 ($3CH_3$), 19.5 (CH_2). LC/MS *m/z*: 352 [M+H]$^+$.

1-cyclopentyl-3-(3-((5,6-dimethyl-2H-benzo[d][1,2,3]triazol-2-yl)methyl)phenyl)urea (33b)

Compound **33b** was obtained, in 33% total yield; mp: 298-299 °C; TLC (petroleum ether/ethyl acetate = 6/4) R_f: 0.46. ^{1}H NMR (400 MHz, DMSO-d_6) δ: 8.28 (1H, s, NH), 7.65 (2H, s, H-4,7), 7.38 (1H, d, *J* = 7.6 Hz, H-4′), 7.19 (1H, t, H-5′), 7.03 (1H, s, H-2′), 6.86 (1H, d, *J* = 6.8 Hz, H-6′), 6.08 (1H, d, *J* = 6.0 Hz, NH), 5.81 (2H, s, CH_2), 3.89 (1H, s, CH), 2.36 (6H, s, $2CH_3$), 1.88-1.75 (2H, m, CH_2), 1.65-1.45 (4H, m, $2CH_2$), 1.35-1.25 (2H, m, CH_2). ^{13}C-NMR (DMSO-d_6) δ: 155.1 (CO), 143.7 (C), 141.3 (C), 140.3 (C), 137.0 (2C), 136.5 (C), 129.6 (CH), 129.4 (CH), 121.0 (CH), 117.6 (CH), 117.2 (CH), 116.7 (2CH), 59.7 (CH_2), 33.2 ($2CH_2$), 23.6 ($2CH_2$), 20.8 ($2CH_3$). LC/MS *m/z*: 364 [M+H]$^+$.

1-cyclohexyl-3-(3-((5,6-dimethyl-2H-benzo[d][1,2,3]triazol-2-yl)methyl)phenyl)urea (34b)

Compound **34b** was obtained, in 73% total yield; mp: 299-300 °C; TLC (petroleum ether/ethyl acetate = 6/4) R_f: 0.26. ^{1}H NMR (400 MHz, DMSO-d_6) δ: 8,33 (1H, s, NH), 7,65 (2H, s, H-4,7), 7,32 (2H, d, *J* = 8.4 Hz, H-2,4′), 7,29 (1H, s, H-2′), 7.19 (1H, t, H-5′), 6.86 (1H, d, *J* = 7.2 Hz, H-6′), 5.99 (1H, d, *J* = 7.6 Hz, NH-CH), 5.81 (1H, s, CH_2), 2.36 (6H, s, $2CH_3$), 1.80-1.46 (4H, m, $2CH_2$), 1.35-1.05 (6H, m, $3CH_2$). ^{13}C-NMR (DMSO-d_6) δ: 154.2 (CO), 143.2 (C), 140.8 (C), 136.5 (2C), 136.0 (2C), 128.9 (CH), 120.5 (CH), 117.9 (CH), 117.2 (CH), 116.7 (CH), 116.2 (2CH), 59.3 (CH_2), 47.5 (CH), 33.3 (CH_2), 25.2 ($2CH_2$), 24.3 ($2CH_2$), 20.3 ($2CH_3$). LC/MS *m/z*: 378 [M+H]$^+$.

N-(3-((5,6-dichloro-1H-benzo[d][1,2,3]triazol-1-yl)methyl)phenyl)acetamide (35a)

Compound **35** was obtained, in 42% total yield; mp: 196-196 °C; TLC (petroleum ether /ethyl acetate 7:3): R_f : 0.09. ^{1}H NMR (400 MHz, DMSO-*d*$_6$) *δ*: 9.93 (1H, s, NH), 8.49 (1H, s, H-4), 8.35 (1H, s, H-7), 7.55 (1H, d, *J* = 8.0 Hz, H-4′), 7.46 (1H, s, H-2′), 7.28 (1H, t, *J* = 7.8 Hz, H-5′), 7.05 (1H, d, *J* = 7.6 Hz, H-6′), 5.97 (2H, s, CH_2), 1.99 (3H, s, CH_3). ^{13}C-NMR (DMSO-*d*$_6$): 168.3 (CO), 144.2 (C), 139.7 (C),135.9 (CH), 132.1 (C), 130.8 (C), 129.2 (CH), 127.2 (C), 122.3 (CH), 120.6 (CH), 118.7 (C), 117.8 (CH), 112.5 (CH), 51.1 (CH_2), 23.9 (2CH_3). LC/MS *m/z* 335, 337 [M+H]$^+$.

N-(3-((5,6-dichloro-1H-benzo[d][1,2,3]triazol-1-yl)methyl)phenyl)propionamide (36a)

Compound **36** was obtained, in 51% total yield; mp: 145-146 °C; TLC (petroleum ether /ethyl acetate 7:3): R_f : 0.21. ^{1}H NMR (400 MHz, DMSO-*d*$_6$) *δ*: 9.86 (1H, s, NH), 8.49 (1H, s, H-2′), 8.35 (1H, s, H-4), 7.57 (1H, t, *J* = 8 Hz, H-4′), 7.49 (1H, s, H-7), 7.28 (1H, t, *J* = 7.8 Hz, H-5′), 7.05 (1H, d, *J* = 7.2 Hz, H-6′), 5.96 (2H, s, CH_2), 2.29-2.24 (2H, m, *CH*$_2$-CH_3), 1.04 (3H, t, *J* = 7.4 Hz, *CH*$_3$-CH_2). ^{13}C-NMR (DMSO-*d*$_6$): 172.0 (CO), 144.2 (C), 139.8 (C),135.9 (CH), 132.1 (C), 130.8 (C), 129.1 (CH), 127.2 (C), 122.2 (CH), 120.6 (CH), 118.7 (C), 117.9 (CH), 112.5 (CH), 51.1 (CH_2), 29.4 (CH_2), 9.5 (CH_3). LC/MS *m/z* 349, 351 [M+H]$^+$.

N-(3-((5,6-dimethyl-1H-benzo[d][1,2,3]triazol-1-yl)methyl)phenyl)acetamide (37b)

Compound **37b** was obtained, in 66% total yield; mp: 198-200 °C; TLC (petroleum ether /ethyl acetate 6:4): R_f : 0.10. ^{1}H NMR (400 MHz, DMSO-*d*$_6$) *δ*: 9.91 (1H,s, NH), 7.80 (1H. s, H-4), 7.53 (3H, m, H-2,2′,4′), 7.40 (1H, s, H-7), 7.26 (1H, t, *J* = 8 Hz, H-5′), 6.98 (1H, d, *J* = 7.6 Hz, H-6′), 5.84 (2H, s, CH_2), 2.35 (6H, s, 2CH_3), 1.98 (3H. s, CH_3CO).^{13}C-NMR (DMSO-*d*$_6$) *δ*:168.3 (CO), 144.5 (C), 139.7 (C), 137.3 (C), 136.6 (C), 133.5 (C), 131.7 (C), 129.0 (CH), 122.1 (CH), 118.5 (CH), 118.1 (CH), 117.7 (CH), 109.7 (CH), 50.7 (CH_2), 23.9 (CH_3), 20.1 (2CH_3). LC/MS *m/z* 295 [M+H]$^+$, 317 [M+Na]$^+$.

N-(3-((5,6-dimethyl-1H-benzo[d][1,2,3]triazol-1-yl)methyl)phenyl)propionamide (38b)

Compound **38b** was obtained, in 25% total yield; mp: 155-157 °C; TLC (petroleum ether /ethyl acetate 6:4): R_f : 0.25. ^{1}H NMR (400 MHz, DMSO-*d*$_6$) *δ*: 9.8 (1H, s, NH), 7.79 (1H, s, H-4), 7.55 (2H, m, H-4′,2′), 7.44 (1H, s, H-7), 7.27 (1H, t, *J* = 7.8 Hz, H-5′), 6.98 (1H, d, *J* = 7.6 Hz, H-6′), 5.86 (2H, s, CH_2), 2.35 (6H, s, 2CH_3), 2.26 (2H, q, *J* = 7.4 Hz, CH_2-CH_3), 1.02 (3H, *J* = 7.8 Hz, CH_3-CH_2).^{13}C-NMR (DMSO-*d*$_6$): 172.0 (CO), 144.6 (C), 139.7 (C), 137.3 (C), 136.5 (C), 133.4 (C), 131.7 (C), 129.0 (CH), 122.5 (CH), 118.5 (CH), 118.1 (CH), 118.0 (CH), 117.7 (CH), 50.8 (CH_2), 29.4 (CH_2), 20.4 (CH_3), 20.6 (CH_3), 10.8 (CH_3-CH_2). LC/MS *m/z* 309 [M+H]$^+$, 331 [M+Na]$^+$.

N-(3-((5,6-dichloro-1H-benzo[d][1,2,3]triazol-1-yl)methyl)phenyl)-4-methylbenzamide (39a)

Compound **39a** was obtained, in 41% total yield; mp: 199-200 °C; TLC (petroleum ether/ethyl acetate = 7/3) R_f : 0.50. ^{1}H NMR (400 MHz, DMSO-*d*$_6$) *δ*: 10.17 (1H, s, NH), 8.51 (1H, s, H-4), 8.39 (1H, s, H-7), 7.83 (2H, d, *J* = 7.6 Hz, H-2″,6″), 7.75 (1H, d, *J* = 8 Hz, H-4′), 7.71 (1H, s, H-2′), 7.36 (1H, t, *J* = 8 Hz, H-5′), 7.32 (2H, d, *J* = 8 Hz, H-3″,5″), 7.13 (1H, d, *J* = 7,6 Hz, H-6′), 6.00 (2H, s, CH_2), 2.38 (3H, s, CH_3). ^{13}C-NMR (DMSO-*d*$_6$): 165.4 (CO), 144.3 (C), 141.6 (C), 139.7 (C), 135.8 (CH), 132.1 (C), 131.8 (C), 130.8 (C), 129.1 (CH), 128.8 (2CH), 127.7 (2CH), 127.2 (C), 122.9 (C), 120.6 (CH), 120.1 (CH), 119.3 (CH), 112.5 (CH), 51.2 (CH_2), 21.0 (CH_3). LC/MS *m/z* 410 [M+H]$^+$, 423 [M+Na]$^+$.

N-(3-((5,6-dichloro-1H-benzo[d][1,2,3]triazol-1-yl)methyl)phenyl)-4-methoxybenzamide (40a)

Compound **40a** was obtained, in 21% total yield; mp: 221-223 °C; TLC (petroleum ether/ethyl acetate = 7/3) R_f : 0.25. ^{1}H NMR (400 MHz, DMSO-*d*$_6$) *δ*: 10.10 (1H, s, NH), 8.52 (1H, s, H-4), 8.39 (1H, s, H-7), 7.92 (2H, d, *J* = 8.4 Hz, H-2″,6″), 7.74 (1H, d, *J* = 8,4 Hz, H-4′), 7.71 (1H, s, H-2′), 7.35 (1H, t, *J* = 8.0 Hz, H-5′), 7.12 (1H, d, *J* = 7,2 Hz, H-6′), 7.06 (2H, d, *J* = 8,4 Hz, H-3″,-5″), 6.00 (2H, s, CH_2), 3.83 (3H, s, OCH_3). ^{13}C-NMR (DMSO-*d*$_6$) *δ*: 164.9 (CO), 161.9 (C), 144.3 (C), 139.8 (C), 135.8 (CH), 132.1 (C), 130.8 (C), 129.6 (2CH), 129.0 (CH), 127.2 (C), 126.7 (C), 122.8 (C), 120.6 (CH), 120.1 (CH), 119.3 (CH), 113.5 (2CH), 112.5 (CH), 55.4 (CH_3), 51.2 (CH_2). LC/MS *m/z* 426, [M+H]$^+$.

N-(3-((5,6-dichloro-1H-benzo[d][1,2,3]triazol-1-yl)methyl)phenyl)-3,4,5-trimethoxybenzamide (41a)

Compound **41a** was obtained, in 22% total yield; mp: 142-144 °C; TLC (petroleum ether/ethyl acetate = 7/3) R_f : 0.15. ^{1}H NMR (400 MHz, DMSO-*d*$_6$) *δ*: 10.15 (1H, s, NH), 8.52 (1H, s, H-4), 8.39 (1H, s, H-7), 7.70 (2H, s, H-2′,4′), 7.37 (1H, t, *J* = 8 Hz, H-5′), 7.23 (2H, s, H-2″,6″), 7.15 (1H, d, *J* = 7,2 Hz, H-6′), 6.00 (2H, s, CH_2), 3.86 (6H, s, 2OCH_3), 3.73 (3H, s, OCH_3). ^{13}C-NMR (DMSO-*d*$_6$) *δ*: 165.0 (CO), 152.6 (2CH), 144.3 (C), 140.3 (C), 139.5 (C), 135.8 (C), 132.0 (C), 130.8 (C), 129.8 (C), 129.1 (CH), 127.3 (C), 123.2 (C), 120.6 (CH), 120.4 (C), 119.7 (CH), 112.5 (CH), 105.3 (2CH), 60.1 (OCH_3), 56.1 (2OCH_3), 51.2 (CH_2). LC/MS *m/z* 487, 489 [M+H]$^+$.

N-(3-((5,6-dichloro-1H-benzo[d][1,2,3]triazol-1-yl)methyl)phenyl)-4-fluorobenzamide (42a)

Compound **42a** was obtained, in 94% total yield; mp: 184-186 °C; TLC (petroleum ether/ethyl acetate = 7/3) R_f : 0.35. ^{1}H NMR (400 MHz, DMSO-*d*$_6$) *δ*: 10.28 (1H, s, NH), 8.52 (1H, s, H-4), 8.39 (1H, s, H-7), 7.99 (2H, d, *J* = 8 Hz, H-2″,6″), 7.74 (1H, d, *J* = 8.4 Hz, H-4′), 7.69 (1H, s, H-2′), 7.38-7.33 (3H, m, H-3″,5″,5′), 7.15 (1H, d, *J* = 7.6 Hz, H-6′), 6.00 (2H, s, CH_2). ^{13}C-NMR (DMSO-*d*$_6$): 164.5 (CO), 164.1 (C), 144.3 (C), 139.5 (C), 135.9 (C), 132.1 (C), 131.2 (C), 130.8 (C), 130.4 (CH), 130.3 (CH), 129.1 (CH), 127.2 (C), 123.1 (CH), 120.6 (CH), 120.1 (CH), 119.3 (CH), 115.4 (CH), 115.2 (CH), 112.5 (CH), 51.2 (CH_2). LC/MS *m/z* 415, 417 [M+H]$^+$.

N-(3-((5,6-dichloro-1H-benzo[d][1,2,3]triazol-1-yl)methyl)phenyl)-4-bromobenzamide (44a)

Compound **44a** was obtained, in 23% total yield; mp: 221-223 °C; TLC (petroleum ether/ethyl acetate = 7/3) R_f : 0.46. ^{1}H NMR (400 MHz, DMSO-d_6) δ: 10.32 (1H, s, NH), 8.51 (1H, s, H-4), 8.39 (1H, s, H-7), 7.87 (2H, d, *J* = 8.4 Hz, H-2″,6″), 7.8-7,0 (3H, m, H-4′,3″,5″), 7.72 (1H, s, H-2′), 7.37 (1H, t, *J* = 8 Hz, H-5′), 7.16 (1H, d, *J* = 7,6 Hz, H-6′), 6.00 (2H, s, CH_2). ^{13}C-NMR (DMSO-d_6): 164.6 (CO), 144.3 (C), 139.4 (C), 135.9 (CH), 133.8 (C), 132.1 (C), 131.4 (2CH), 130.8 (C), 129.8 (2CH), 129.1 (CH), 127.2 (C), 125.4 (C), 123.2 (C), 120.6 (CH), 120.1 (CH), 119.3 (CH), 112.5 (CH), 51.1 (CH_2). LC/MS *m/z* 477, 475 [M+H]$^+$.

4-cyano-N-(3-((5,6-dichloro-1H-benzo[d][1,2,3]triazol-1-yl)methyl)phenyl)benzamide (45a)

Compound **45a** was obtained, in 24% total yield; mp: 167-169 °C; TLC (petroleum ether/ethyl acetate = 7/3) R_f : 0.37. ^{1}H NMR (400 MHz, DMSO-d_6) δ: 10.48 (1H, s, NH), 8.51 (1H, s, H-4), 8.39 (1H, s, H-7), 8.06 (2H, d, *J* = 8 Hz, H-2″,6″), 8.0 (2H, d, *J* = 8.4 Hz, H-3″,5″), 7.75 (1H, d, *J* = 8.0 Hz, H-4′), 7.69 (1H, s, H-2′),7.38 (1H, t, *J* = 7.8 Hz, H-5′), 7.18 (1H, d, *J* = 7.6 Hz, H-6′), 6.02 (2H, s, CH_2). ^{13}C-NMR (DMSO-d_6) δ: 164.2 (CO), 144.3 (C), 139.2 (C), 138.7 (C), 136.0 (C), 132.4 (2CH), 132.1 (C), 130.8 (C), 129.2 (CH), 128.5 (2CH), 127.2 (C), 123.5 (CH), 120.6 (CH), 120.2 (CH), 119.3 (CH), 118.3 (C≡N), 113.9 (C), 112.5 (CH), 51.1 (CH_2). LC/MS *m/z* 422, 424 [M+H]$^+$.

N-(3-((5,6-dichloro-1H-benzo[d][1,2,3]triazol-1-yl)methyl)phenyl)-4-(trifluoromethyl)benzamide (46a)

Compound **46a** was obtained, in 44% total yield; mp: 211-213 °C; TLC (petroleum ether/ethyl acetate = 7/3) R_f : 0.67. ^{1}H NMR (400 MHz, DMSO-d_6) δ: 10.47 (1H, s, NH), 8.52 (1H, s, H-4), 8.39 (1H, s, H-7), 8.11 (2H, d, *J* = 7.6 Hz, H-2″,6″), 7.90 (2H, d, *J* = 8 Hz, H-3″,5″), 7.76 (1H, d, *J* = 8 Hz, H-4′), 7.69 (1H, d, H-2′), 7.39 (1H, t, *J* = 7.8 Hz, H-5′), 7.17 (1H, d, *J* = 6.8 Hz, H-6′), 6.02 (2H, s, CH_2). ^{13}C-NMR (DMSO-d_6) δ: 164.7 (CO), 145.7 (C), 139.1 (C), 137.8 (C), 137.5 (C), 135.9 (C), 133.2 (C), 131.4 (2C), 130.4 (CH), 130.5 (CH), 127.9 (CH), 127.2 (C), 123.8 (CH), 120.6 (CH), 120.3 (CH), 119.3 (CH), 115.4 (CH), 115.2 (CH), 113.0 (CH), 51.2 (CH_2). LC/MS *m/z* 465, 467 [M+H]$^+$.

N-(3-((5,6-dichloro-1H-benzo[d][1,2,3]triazol-1-yl)methyl)phenyl)-4-nitrobenzamide (47a)

Compound **47a** was obtained, in 14% total yield; mp: 217-218 °C; TLC (petroleum ether/ethyl acetate = 7/3) R_f: 0.29. ^{1}H NMR (400 MHz, DMSO-d_6) δ: 10.57 (1H, s, NH), 8.52 (1H, s, H-4), 8.40 (1H, s, H-7), 8.35 (2H, d, *J* = 8.8 Hz, H-3″,5″), 8.14 (1H, d, *J* = 8.4 Hz, H-2″,6″), 7.77 (1H, s, H-4′), 7.69 (1H, s, H-2′), 7.40 (1H, t, *J* = 7,8 Hz, H-5′), 7.19 (1H, d, *J* = 7,6 Hz, H-6′), 6.02(2H, s, CH_2). ^{13}C-NMR (DMSO-d_6) δ: 164.0 (CO), 149.2 (C), 144.3 (C), 140.4 (C), 139.2 (C), 136.0 (C), 132.1 (C), 130.8 (C), 129.2 (CH), 129.2 (2CH), 127.3 (C), 123.9 (CH), 123.5 (2CH), 120.7 (CH), 120.2 (CH), 119.4 (CH), 112.5 (CH), 51.1 (CH_2). LC/MS *m/z* 442, 444 [M+H]$^+$.

4-amino-N-(3-((5,6-dichloro-1H-benzo[d][1,2,3]triazol-1-yl)methyl)phenyl)benzamide (48a)

This compound was obtained starting from compound **47a** (7.43 mmol) in 50 ml of ethanol and 0.88 mL of methylhydrazine. It was heated in autoclave for 24h at 100 °C. In the end, the mother was evaporated in vacuo and the crude residue is triturated with petrol ether to obtain compound **48**. The crude product was not further purified.

Compound **48a** was obtained, in 66% total yield; mp: 101-102 °C; TLC (petroleum ether/ethyl acetate = 6/4) R_f : 0.18. ^{1}H NMR (400 MHz, DMSO-d_6) δ: 9.77 (1H, s, NH), 8.50 (1H, s, H-4), 8.37 (1H, s, H-7), 7.73-7.66 (4H, m, H-6′,2′,2″,6″), 7.31 (1H, t, *J* = 7.8 Hz, H-5′), 7.06 (1H, d, *J* = 7.2 Hz, H-4′), 6.58 (2H, d, *J* = 8 Hz, H-3″,5″), 5.98 (2H, s, CH_2), 5.76 (2H, s, NH_2). ^{13}C-NMR (DMSO-d_6) δ: 165.3 (CO), 152.2 (C), 144.3 (C), 140.2 (C), 135.7 (C), 132.1 (C), 130.7 (C), 129.3 (2CH), 128.9 (CH), 127.2 (C), 122.3 (CH), 120.8 (C), 120.6 (CH), 119.8 (CH), 119.1 (CH), 112.5 (CH), 112.5 (2CH), 51.3 (CH_2). LC/MS *m/z* 412, 414 [M+H]$^+$.

N-(3-((5,6-dimethyl-1H-benzo[d][1,2,3]triazol-1-yl)methyl)phenyl)-4-methylbenzamide (49b)

Compound **49b** was obtained, in 57% total yield; mp: 211-212 °C; TLC (petroleum ether/ethyl acetate = 7/3) R_f : 0.27. ^{1}H NMR (400 MHz, DMSO-d_6) δ: 10.16 (1H, s, NH), 7.82 (3H, m, H-2″,6″,4), 7.3 (2H, d, *J* = 8.4 Hz, H-4′), 7.7 (1H, s, H-7), 7.58 (1H, s, H-2′), 7.32 (3H, m, H-3″,5″,5′), 7.05 (1H, *J* = 7.2 Hz, 6′), 5.89 (2H, s, CH_2). ^{13}C-NMR (DMSO-d_6) δ: 165.3 (CO), 144.6 (C), 141.6 (C), 139.6 (C), 137.3 (C), 136.5 (C), 133.5 (C), 131.7 (C), 131.7 (C), 128.9 (CH), 128.8 (CH), 127.7 (CH), 122.7 (CH), 119.9 (CH), 119.2 (CH), 118.1 (CH), 109.7 (CH), 50.8 (CH_2), 21.0 (CH_3), 20.4 (CH_3), 19.8 (CH_3). LC/MS *m/z* 371 [M+H]$^+$.

N-(3-((5,6-dimethyl-1H-benzo[d][1,2,3]triazol-1-yl)methyl)phenyl)-4-methoxylbenzamide (50b)

Compound **50b** was obtained, in 17% total yield; mp: 203-206 °C; TLC (petroleum ether/ethyl acetate = 6/4) R_f : 0.36. ^{1}H NMR (400 MHz, DMSO-d_6) δ: 10.09 (1H, s, NH), 7.91 (2H, d, *J* = 7.9 Hz, H-2″, 6″), 7.80 (1H, s, H-4′), 7.72 (1H, d, *J* = 7.7 Hz, H-4′), 7.68 (1H, s, H-2′), 7.59 (1H, s, H-7), 7.32 (1H, t, *J* = 7.3 Hz, H-5′), 7.04 (3H, m, H-6′,3″,5″), 5.89 (2H, s, CH_2). ^{13}C-NMR (DMSO-d_6) δ: 164.9 (CO), 161.9 (C), 144.6 (C), 139.7 (C), 137.3 (C), 136.4 (C), 133.5 (C), 131.7 (C), 129. 6 (CH), 129.3 (CH), 128.9 (CH), 126.7 (C), 122.6 (CH), 119.9 (CH), 119.2 (CH), 118.1 (CH), 113.7 (CH), 113.5 (CH), 109.7 (CH), 55.4 (OCH_3), 50.8 (CH_2), 20.1 ($2CH_3$). LC/MS *m/z* 387 [M+H]$^+$, 409 [M+Na]$^+$.

N-(3-((5,6-dimethyl-1H-benzo[d][1,2,3]triazol-1-yl)methyl)phenyl)-3,4,5-trimethoxylbenzamide (51b)

Compound **51b** was obtained, in 20% total yield; mp: 160-162 °C; TLC (petroleum ether/ethyl acetate = 6/4) R_f : 0.21. ^{1}H NMR (400 MHz, DMSO-d_6) δ: 10.13 (NH), 7.80 (1H, s, H-4), 7.67 (2H, m, H-4′,2′), 7.59 (1H, s, H-7), 7.35 (3H,

t, J = 8.0 Hz, H-5′), 7.24 (2H, s, H-2″,6″), 7.08 (1H, d, J = 7.2, H-6′) 5.9 (2H, s, CH_2) 3.85 (6H, s, $2OCH_3$), 3.72 (3H, s, OCH_3), 2.35 (6H, s, $2CH_3$). ^{13}C-NMR (DMSO-d_6) δ: 165.3 (CO), 152.7 (C),152.6 (C), 144.6 (C), 140.4 (C), 139.4 (C), 137.4 (C), 136.5 (C), 133.5 (C), 131.7 (C), 129.8 (C), 129.0 (CH), 127.6 (CH), 123.0 (CH), 120.2 (CH), 119.5 (CH), 118.1 (CH), 109.7 (CH), 105.3 (CH), 105.0 (CH), 60.1 (OCH_3), 56.1 (OCH_3), 56.0 (OCH_3), 50.8 (CH_2), 20.1 (CH_3). LC/MS *m/z* 447 $[M+H]^+$, 469 $[M+Na]^+$.

N-(3-((5,6-dimethyl-1H-benzo[d][1,2,3]triazol-1-yl)methyl)phenyl)-4-fluorobenzamide (52b)

Compound **52b** was obtained, in 98% total yield; mp: 143-147 °C; TLC (petroleum ether/ethyl acetate = 7/3) R_f: 0.22. ^{1}H NMR (400 MHz, DMSO-d_6) δ: 10.26 (1H, s, NH), 7.99 (2H, t, H-3″,5″), 7.72 (1H, d, J = 7.72 Hz, H-4′), 7,66 (1H, s, H-7), 7.58 (1H, s, H-2′), 7.34 (3H, m, H-2″,6″,5′), 7.08 (1H, d, J = 7.08, H-6′), 5.90 (2H, s, $2CH_2$), 2.35 (6H, s, $2CH_3$). ^{13}C-NMR (DMSO-d_6) δ: 164.1 (CO), 144.6 (C), 139.5 (C), 137.4 (C), 136.5 (C), 133.5 (C), 131.7 (C), 131.2 (C), 130.4 (2CH), 129.0 (CH), 122.9 (CH), 119.9 (CH), 119.2 (CH), 118.1 (CH), 115.2 (CH), 109.7 (CH), 50.8 (CH_2), 20.4 (CH_3), 19.8 (CH_3). LC/MS *m/z* 375 $[M+H]^+$.

N-(3-((5,6-dimethyl-1H-benzo[d][1,2,3]triazol-1-yl)methyl)phenyl)-4-chlorobenzamide (53b)

Compound **53b** was obtained, in 56% total yield; mp: 227-229 °C; TLC (petroleum ether/ethyl acetate = 7/3) R_f : 0.30. ^{1}H NMR (400 MHz, DMSO-d_6) δ: 10.65 (1H, s, NH), 7.94 (2H, d, J = 8 Hz, H-3″,5″), 7.80 (1H, s, H-2′), 7.730 (1H, d, J = 8 Hz, H-4′), 7.59 (2H, d, J = 7.2 Hz, H-3″,5″), 7.08 (1H, d, J = 7.6 Hz, H-6′), 5.90 (2H, s, CH_2), 2.36 (6H, s, $2CH_3$). ^{13}C-NMR (DMSO-d_6) δ: 169.9 (CO), 149.8 (C), 144.6 (C), 142.6 (C), 141.8 (C), 141.7 (C), 138.7 (C), 138.7 (C), 137.0 (C), 134.8 (2CH), 134.3 (CH), 133.6 (2CH), 128.3 (CH), 125.2 (CH), 124.5 (CH), 123.4 (CH), 115.0 (CH), 56.0 (CH_2), 25.6 (CH_3), 25.1 (CH_3). LC/MS *m/z* 391 $[M+H]^+$, 413 $[M+Na]^+$.

N-(3-((5,6-dimethyl-1H-benzo[d][1,2,3]triazol-1-yl)methyl)phenyl)-4-bromobenzamide (54b)

Compound **54b** was obtained, in 15% total yield; mp: 237-238 °C; TLC (petroleum ether/ethyl acetate = 7/3) R_f: 0.25. ^{1}H NMR (400 MHz, DMSO-d_6) δ: 7.78 (2H, d, J = 8.4 Hz,H-3″,5″), 7.80 (1H, s, H-4), 7.72 (3H, m, H-4′,2″, 6″),7.65 (1H, s, H-2′), 7.59 (1H, s, H-7), 7.34 (1H, t, J = 7.6 Hz, H-5′), 7.08 (2H, d, J = 7.6 Hz, H-6′), 5.9 (2H, s, $2CH_2$), 2.35 (6H, s, $2CH_3$). ^{13}C-NMR (DMSO-d_6) δ: 164.5 (CO), 144.6 (C), 139.4 (C), 137.4 (C), 136.6 (CH), 133.8 (C), 133.5 (C), 131.7 (C), 131.3 (2CH), 129.8 (2CH), 129.0 (CH), 125.4 (C), 123.0 (C), 119.9 (CH), 118.1 (CH), 109.7 (CH), 50.8 (CH_2), 20.1 (CH_3). LC/MS *m/z* 436 $[M+H]^+$.

4-cyano-N-(3-((5,6-dimethyl-1H-benzo[d][1,2,3]triazol-1-yl)methyl)phenyl)benzamide (55b)

Compound **55b** was obtained, in 26% total yield; mp: 242-247 °C; TLC (petroleum ether/ethyl acetate = 6/4) R_f : 0.3. ^{1}H NMR (400 MHz, DMSO-d_6) δ: 7.85 (2H, d, J = 7.9 Hz, H-2″,6″), 7.86 (2H, d, J = 7.76, H-3″,5″), 7.28 (1H, s, H-2′), 7.62 (1H, d, J = 7.6 Hz, H-4′), 7.52 (1H, s, H-4), 7.38 (1H, s, H-7), 7.27 (1H, t, J = 7.3Hz, H-5′), 7.00 (1H, d, J= 7.03 Hz, H-6′), 5.71 (2H. s, CH_2), 2,35 (6H, s, $2CH_3$). ^{13}C-NMR (DMSO-d_6) δ: 164.2 (CO), 144.6 (C), 139.1 (C), 138.8 (C), 136.8 (C), 136.6 (C), 133.5 (C), 132.4 (2CH), 131.7 (C), 129.3 (CH), 128.5 (CH), 132.3 (CH), 120.0 (CH), 119.3 (CH), 118.2 (CN), 118.1 (CH), 113.8 (C), 109.7 (CH), 50.6 (CH_2), 20.4 (CH_3), 19.8 (CH_3). LC/MS *m/z* 382 $[M+H]^+$.

N-(3-((5,6-dimethyl-1H-benzo[d][1,2,3]triazol-1-yl)methyl)phenyl)-4-(trifluoromethyl)benzamide (56b)

Compound **56b** was obtained, in 20% total yield; mp: 201-203 °C; TLC (petroleum ether/ethyl acetate = 6/4) R_f : 0.84. ^{1}H NMR (400 MHz, DMSO-d_6) δ: 10.47 (1H, s, NH), 8.10 (2H, d, J = 8 Hz, H-2″,6″), 7.89 (2H, d, J = 8.4 Hz, H-3″,5″), 7.80 (1H, s, H-4), 7.74 (1H, d, J = 8Hz, H-6′), 7.66 (1H, s, H-2′), 7.59 (1H, s, H-7), 7.36 (1H, t, J = 8.0 Hz, H-5′), 7.12 (1H, d, J =7.1 Hz, H-4′), 5.91 (2H, s, CH_2), 2.36 (6H, s, $2CH_3$). ^{13}C-NMR (DMSO-d_6) δ: 164.4 (CO), 145.5 (C), 137.5 (C), 136.8 (2C), 136.4 (C), 135.8 (C), 134.4 (C), 132.8 (C), 128.8 (CH), 127.8 (2CH), 125.2 (2CH), 124.1 (2CH), 123.2 (CH), 121.3 (CH), 118.6 (CH), 50.7 (CH_2), 20.4 (CH_3), 19.8 (CH_3). LC/MS *m/z* 425 $[M+H]^+$.

N-(3-((5,6-dimethyl-1H-benzo[d][1,2,3]triazol-1-yl)methyl)phenyl)-4-nitrobenzamide (57b)

Compound **57b** was obtained, in 31% total yield; mp: 227-230 °C; TLC (petroleum ether/ethyl acetate = 4/6) R_f : 0.76. ^{1}H NMR (400 MHz, DMSO-d_6) δ: 10.57 (1H, s, NH), 8.34 (2H, d, J = 8.2 Hz, 3″,5″), 8.12 (2H, d, J = 8.2 Hz, H-2″,6″), 7-80 (1H, s, H-2′), 7.75 (1H, d, J = 9.2 Hz, H-4′), 7.64 (1H, s, H-4), 7.59 (1H, s, H-7), 7.36 (1H, t, J = 8 Hz, H-5′), 7.12 (1H, d, J = 7.6, H-6′), 5.92 (2H, s, CH_2), 2.35 ($2CH_3$). ^{13}C-NMR (DMSO-d_6) δ: 163.9 (CO), 149.1 (C), 144.6 (C), 140.4 (C), 139.1 (C), 137.4 (C), 136.7 (C), 133.5 (C), 131.7 (C), 129.2 (2CH), 129.1 (CH), 123.5 (CH), 123.3 (CH), 122.7 (CH), 120.0 (CH), 119.3 (CH), 118.1 (CH), 109.7 (CH), 50.7 (CH_2), 20.1 ($2CH_3$). LC/MS *m/z* 402 $[M+H]^+$.

1-(3-((5,6-dichloro-1H-benzo[d][1,2,3]triazol-1-yl)methyl)phenyl)-3-ethylurea (58a)

Compound **58a** was obtained, in 44% total yield; mp: 244-245 °C; TLC (petroleum ether/ethyl acetate = 6/4) R_f : 0.15. ^{1}H NMR (400 MHz, DMSO-d_6) δ: 8.49 (1H, s, H-4), 8.44 (1H, s, NH), 8.37 (1H, s, H-7), 7.33 (1H, d, J = 8,4 Hz, H-4′), 7.31 (1H, s, H-2′), 7.19 (1H, t, H-5′), 6.88 (1H, d, J = 7.6 Hz, H-6′), 6.03 (1H, t, NH), 5.92 (2H, s, CH_2), 3.07 (2H, q, CH_2), 1.02 (3H, t, CH_3). ^{13}C-NMR (DMSO-d_6) δ: 154.9 (CO), 144.2 (C), 141.1 (C), 135.8 (C), 132.0 (C), 130.7 (C), 129.1 (CH), 127.2 (C), 120.6 (CH), 120.2 (CH), 117.3 (CH), 116.5 (CH), 112.5 (CH), 51.3 (CH_2), 34.0 (CH_2), 15.4 (CH_3). LC/MS *m/z*: 364 $[M+H]^+$.

1-(3-((5,6-dichloro-1H-benzo[d][1,2,3]triazol-1-yl)methyl)phenyl)-3-propylurea (59a)

Compound **59a** was obtained, in 45% total yield; mp: >300 °C; TLC (petroleum ether/ethyl acetate = 6/4) R_f: 0.38. ^{1}H NMR (400 MHz, DMSO-d_6) δ: 8.50 (1H, s, H-4), 8.43 (1H, s, NH), 8.37 (1H, s, H-7), 7.33 (1H, d, *J* = 8.0 Hz, H-4′), 7.32 (1H, s, H-2′), 7.20 (1H, t, H-5′), 6.88 (1H, d, *J* = 7.6 Hz, H-6′,), 6.07 (1H, t, NH), 5.92 (2H, s, CH_2), 3.00 (2H, q, CH_2), 1.43-1.38 (2H, m, CH_2), 0.85 (3H, t, CH_3). ^{13}C-NMR (DMSO-d_6) δ: 155.0 (CO), 144.2 (C), 141.1 (C), 135.8 (C), 132.1 (C), 130.7 (C), 129.1 (CH), 127.2 (C), 121.2 (CH), 120.6 (CH), 117.3 (CH), 116.5 (CH), 112.5 (CH), 51.3 (CH_2), 40.8 (CH_2), 22.9 (CH_2), 11.3 (CH_3). LC/MS *m*/*z*: 378 $[M+H]^+$.

1-butyl-3-(3-((5,6-dichloro-1H-benzo[d][1,2,3]triazol-1-yl)methyl)phenyl)urea (60a)

Compound **60a** was obtained, in 19% total yield; mp: 232-233 °C; TLC (petroleum ether/ethyl acetate = 6/4) R_f: 0.41. ^{1}H NMR (400 MHz, DMSO-d_6) δ: 8.49 (1H, s, H-4), 8.42 (1H, s, NH), 8.35 (1H, s, H-7), 7.33 (1H, d, *J* = 8.0 Hz, H-4′,), 7.30 (1H, s, H-2′), 7.19 (1H, t, H-5′), 6.88 (1H, d, *J* = 7.6 Hz, H-6′,), 6.05 (1H, t, NH), 5.92 (2H, s, CH_2), 3.04 (2H, q, $\underline{CH_2}$-CH_3), 1.41-1.31 (2H, m, CH_2), 1.31-1.27 (2H, m, CH_2), 0.87 (3H, t, CH_3). ^{13}C-NMR (DMSO-d_6) δ: 155.5 (CO), 144.7 (C), 141.5 (C), 136.3 (C), 132.5 (C), 131.2 (C), 129.6 (CH), 127.7 (C), 121.1 (CH), 120.7 (CH), 117.7 (CH), 116.9 (CH), 113.0 (CH), 51.7 (CH_2), 32.3 (CH_2), 19.9 (CH_2), 14.1 (CH_3). LC/MS *m*/*z*: 392, 394 $[M+H]^+$.

1-cyclopentyl-3-(3-((5,6-dichloro-1H-benzo[d][1,2,3]triazol-1-yl)methyl)phenyl)urea (61a)

Compound **61a** was obtained, in 10% total yield; mp: 231-232 °C; TLC (petroleum ether/ethyl acetate = 6/4) R_f: 0.36. ^{1}H NMR (400 MHz, DMSO-d_6) δ: 8.49 (1H, s, H-4), 8.34 (1H, s, NH), 8.28 (1H, s, H-7), 7.28 (1H, s, H-2′), 7.31 (1H, d, *J* = 8.0 Hz, H-4′), 7.19 (1H, t, H-5′), 6.87 (1H, d, *J* = 7.6 Hz, H-6′,), 6.08 (1H, d, *J* = 6.8 Hz, NH,), 5.92 (2H, s, CH_2), 1.83-1.78 (1H, m, CH), 1.61-1.59 (2H, m, CH_2), 1.53-1.50 (2H, m, CH_2), 1.34-1.30 (4H, m, $2CH_2$). ^{13}C-NMR (DMSO-d_6) δ: 154.6 (CO), 144.2 (C), 141.0 (C), 135.8 (C), 132.0 (C), 130.7 (C), 129.2 (CH), 127.2 (C), 120.6 (CH), 120.2 (CH), 117.2 (CH), 116.4 (CH), 112.5 (CH), 51.3 (CH_2), 50.8 (CH), 32.7 ($2CH_2$), 23.1 ($2CH_2$). LC/MS *m*/*z*: 404 $[M+H]^+$.

1-cyclohexyl-3-(3-((5,6-dichloro-1H-benzo[d][1,2,3]triazol-1-yl)methyl)phenyl)urea (62a)

Compound **62a** was obtained, in 10% total yield; mp: 200-201 °C; TLC (petroleum ether/ethyl acetate = 6/4) R_f: 0.18. ^{1}H NMR (400 MHz, DMSO-d_6) δ: 10.17 (1H, s, NH), 8.49 (1H, s, H-4), 8.37 (1H, s, H-7), 7.52 (1H, s, H-2′), 7.37-7.27 (2H, m, H-4′,5′), 7.06 (1H, d, *J* = 6.8 Hz, H-6′), 6.08-5.87 (1H, m, NH-CH), 5.56 (2H, d, *J* = 5.2 Hz, CH_2), 1.73-1.42 (4H, m, $2CH_2$), 1.23-1.03 (6H, m, $3CH_2$). ^{13}C-NMR (DMSO-d_6) δ: 156.6 (CO), 144.2 (C), 138.6 (C), 136.2 (C), 132.1 (C), 130.8 (C), 129.4 (CH), 129.2 (C), 122.9 (CH), 120.6 (CH), 118.9 (CH), 118.1 (CH), 112.5 (CH), 51.0 (CH_2), 47.5 (CH-N), 33.3 ($3CH_2$), 24.4 ($2CH_2$). LC/MS *m*/*z*: 404 $[M+H]^+$.

1-(3-((5,6-dimethyl-1H-benzo[d][1,2,3]triazol-1-yl)methyl)phenyl)-3-ethylurea (63b)

Compound **63b** was obtained, in 79% total yield; mp: 271-273 °C; TLC (petroleum ether/ethyl acetate = 6/4) R_f: 0.07. ^{1}H NMR (400 MHz, DMSO-d_6) δ: 8.45 (1H, s, NH), 7.79 (1H, s, H-4), 7.55 (1H, s, H-7), 7.33 (1H, d, *J* = 9.2 Hz, H-6'), 7.23 (1H, s, H-2'), 7.17 (1H, t, H-5'), 6.81 (1H, d, *J* = 7.6 Hz, H-4'), 6.03 (1H, t, NH), 5.82 (2H, s, CH_2), 3.12-3.00 (2H, m, CH_2), 2.35 (6H, s, $2CH_3$), 1.01 (3H, t, CH_3). ^{13}C-NMR (DMSO-d_6) δ: 155.0 (CO), 144.5 (C), 140.9 (C), 137.4 (C), 136.4 (C), 133.5 (C), 131.7 (C), 129.0 (CH), 120.0 (CH), 118.1 (CH), 117.1 (CH), 116.4 (CH), 109.7 (CH), 50.9 (CH_2), 33.9 (CH_2), 20.4 (CH_3), 19.8 (CH_3), 15.4 (CH_3). LC/MS *m*/*z*: 324 $[M+H]^+$.

1-(3-((5,6-dimethyl-1H-benzo[d][1,2,3]triazol-1-yl)methyl)phenyl)-3-propylurea (64b)

Compound **64b** was obtained, in 31% total yield; mp: 189-191 °C; TLC (petroleum ether/ethyl acetate = 6/4) R_f: 0.14. ^{1}H NMR (400 MHz, DMSO-d_6) δ: 8.42 (1H, s, NH), 7.79 (1H, s, H-4), 7.55 (1H, s, H-7), 7.33 (1H, d, *J* = 8.8 Hz, H-6'), 7.22 (1H, s, H-2'), 7.17 (1H, t, H-5'), 6.81 (1H, d, *J* = 7.6 Hz, H-4'), 6.06 (1H, t, NH), 5.82 (2H, s, CH_2), 3.05-2.95 (2H, m, CH_2), 2.35 (6H, s, $2CH_3$), 1.40 (2H, q, CH_2-NH), 0.84 (3H, t, CH_3). ^{13}C-NMR (DMSO-d_6) δ: 155.0 (CO), 144.5 (C), 141.0 (C), 137.4 (C), 136.5 (C), 133.5 (C), 131.7 (C), 129.2 (CH), 120.0 (CH), 118.1 (CH), 117.1 (CH), 116.3 (CH), 109.7 (CH), 50.8 (CH_2), 40.8 (CH_2), 22.9 (CH_2), 20.4 (CH_3), 19.8 (CH_3), 11.3 (CH_3). LC/MS *m*/*z*: 338$[M+H]^+$.

1-(sec-butyl)-3-(3-((5,6-dimethyl-1H-benzo[d][1,2,3]triazol-1-yl)methyl)phenyl)urea (65b)

Compound **65b** was obtained, in 31% total yield; mp: 202-204 °C; TLC (petroleum ether/ethyl acetate = 6/4) R_f: 0.22. ^{1}H NMR (400 MHz, DMSO-d_6) δ: 8.31 (1H, s, NH), 7.79 (1H, s, H-4), 7.55 (1H, s, H-7), 7.31 (1H, d, *J* = 7.6 Hz, H-6'), 7.22 (1H, s, H-2'), 7.17 (1H, t, H-5'), 6.81 (1H, d, *J* = 7.6 Hz, H-4'), 5.86 (1H, q, NH), 5.82 (2H, s, CH_2), 2.35 (6H, s, $2CH_3$), 1.45-1.38 (1H, m, CH), 1.02 (3H, t, CH_3), 0.83 (2H, t, CH_2). ^{13}C-NMR (DMSO-d_6) δ: 154.5 (CO), 144.5 (C), 140.9 (C), 137.4 (C), 136.5 (C), 133.5 (C), 131.7 (C), 129.0 (CH), 120.0 (CH), 118.1 (CH), 117.0 (CH), 116.2 (CH), 109.7 (CH), 50.8 (CH_2), 46.0 (CH), 29.2 (CH_2), 20.6 (CH_3), 20.4 (CH_3), 19.8 (CH_3), 10.2 (CH_3). LC/MS *m*/*z*: 352 $[M+H]^+$.

1-butyl-3-(3-((5,6-dimethyl-1H-benzo[d][1,2,3]triazol-1-yl)methyl)phenyl)urea (66b)

Compound **66b** was obtained, in 37% total yield; mp: 195-197 °C; TLC (petroleum ether/ethyl acetate = 6/4) R_f: 0.18. ^{1}H NMR (400 MHz, DMSO-d_6) δ: 8.40 (1H, s, NH), 7.79 (1H, s, H-4), 7.55 (1H, s, H-7), 7.33 (1H, d, *J* = 8.00 Hz, H-6'), 7.22 (1H, s, H-2'), 7.17 (1H, t, H-5'), 6.81 (1H, d, *J* = 7.6 Hz, H-4'), 6.03 (1H, t, NH), 5.82 (2H, s, CH_2), 3.04 (2H, q, CH_2), 2.35 (6H, s, $2CH_3$), 1.45-1.39 (2H, m, CH_2), 1.39-1.20 (2H, m, CH_2), 0.87 (3H, t, CH_3). ^{13}C-NMR (DMSO-d_6) δ:

160.2 (CO), 149.8 (C), 146.2 (C), 142.6 (C), 141.7 (C), 138.7 (C), 137.0 (C), 134.3 (CH), 125.2 (CH), 123.3 (CH), 122.3 (CH), 121.5 (CH), 115.0 (CH), 56.1 (CH_2), 43.9 (CH_2), 37.0 (CH_3), 25.6 (CH_3), 25.1 (CH_3), 24.7 (CH_2), 18.9 (CH_3). LC/MS *m/z*: 352 $[M+H]^+$.

1-(tert-butyl)-3-(3-((5,6-dimethyl-1H-benzo[d][1,2,3]triazol-1-yl)methyl)phenyl)urea (67b)

Compound **67b** was obtained, in 22% total yield; mp: 189-191 °C; TLC (petroleum ether/ethyl acetate = 6/4) R_f: 0.33. ^{1}H NMR (400 MHz, DMSO-d_6) δ: 8.24 (1H, s, NH), 7.79 (1H, s, H-4), 7.56 (1H, s, H-7), 7.26 (1H, d, *J* = 8.4 Hz, H-6'), 7.23 (1H, s, H-2'), 7.16 (1H, t, H-5'), 6.78 (1H, d, *J* = 7.6 Hz, H-4'), 5.91 (1H, s, NH), 5.82 (2H, s, CH_2), 2.35 (6H, s, CH_3), 1.26 (9H, s, CH_3). ^{13}C-NMR (DMSO-d_6) δ: 159.4 (CO), 149.8 (C), 146.2 (C), 142.6 (C), 141.7 (C), 138.7 (C), 137.0 (C), 134.3 (C), 125.1 (CH), 123.3 (CH), 122.1 (CH), 121.4 (CH), 115.0 (CH), 56.1 (C), 54.6 (CH_2), 34.2 ($3CH_3$), 25.6 (CH_3), 25.1 (CH_3). LC/MS *m/z*: 352 $[M+H]^+$.

1-cyclopentyl-3-(3-((5,6-dimethyl-1H-benzo[d][1,2,3]triazol-1-yl)methyl)phenyl)urea (68b)

Compound **68b** was obtained, in 33% total yield; mp: 290-292 °C; TLC (petroleum ether/ethyl acetate = 6/4) R_f: 0.21. ^{1}H NMR (400 MHz, DMSO-d_6) δ: 8.25 (1H, s, NH), 7.79 (1H, s, H-4), 7.55 (1H, s, H-7), 7.31 (1H, d, *J* = 8.4 Hz, H-6'), 7.20 (1H, s, H-2'), 7.17 (1H, t, H-5'), 6.81 (1H, d, *J* = 7.6 Hz, H-4'), 6.06 (1H, d, NH), 3.95-3.8 (1H, m, CH), 2.35 (6H, s, CH_3), 1.89-1.72(2H, m, CH_2), 1.68-1.48 (4H, m, $2CH_2$), 1.33 (2H, t, CH_2). ^{13}C-NMR (DMSO-d_6) δ: 154.6 (CO), 144.5 (C), 140.9 (C), 137.3 (C), 136.5 (C), 133.4 (C), 131.7 (C), 129.0 (CH), 120.0 (CH), 118.1 (CH), 117.0 (CH), 116.2 (CH), 109.7 (CH), 50.8 (CH), 50.8 (CH_2), 32.8 ($2CH_2$), 23.1 ($2CH_2$), 20.4 (CH_3), 19.8 (CH_3). LC/MS *m/z*: 364 $[M+H]^+$.

1-cyclohexyl-3-(3-((5,6-dimethyl-1H-benzo[d][1,2,3]triazol-1-yl)methyl)phenyl)urea (69b)

Compound **69b** was obtained, in 25% total yield; mp: 233-235 °C; TLC (petroleum ether/ethyl acetate = 6/4) R_f: 0.26. ^{1}H NMR (400 MHz, DMSO-d_6) δ: 8.30 (1H, s, NH), 7.79 (1H, s, H-4), 7.55 (1H, s, H-7), 7.31 (1H, d, *J* = 8.8 Hz, H-6'), 7.22-7.11 (2H, m, H-5'/ H-2'), 6.81 (1H, d, *J* = 7.6 Hz, H-4'), 5.98 (1H, d, *J* = 7.6 Hz, NH), 5.82 (2H, s, CH_2), 3.54 (1H, m, CH), 2.35 (6H, s, $2CH_3$), 1.8-1.48 (4H, m, $2CH_2$), 1.35-1.05 (6H, m, $3CH_2$). ^{13}C-NMR (DMSO-d_6) δ: 154.2 (CO), 144.5 (C), 140.9 (C), 137.3 (C), 136.5 (C), 133.5 (C), 131.7 (C), 129.0 (CH), 120.0 (CH), 118.1 (CH), 117.0 (CH), 116.1 (CH), 109.7 (CH), 50.8 (CH_2), 47.5 (CH), 32.8 (CH_2), 25.2 ($2CH_2$), 24.2 ($2CH_2$), 20.4 (CH_3), 19.8 (CH_3). LC/MS *m/z*: 378 $[M+H]^+$.

1-(3-((5,6-dimethyl-1H-benzo[d][1,2,3]triazol-1-yl)methyl)phenyl)-3-(4-methoxyphenyl)urea (70b)

Compound **70b** was obtained, in 29% total yield; mp: 233-235 °C; TLC (petroleum ether/ethyl acetate = 6/4) R_f: 0.15. ^{1}H NMR (400 MHz, DMSO-d_6) δ: 8.65 (1H, s, NH), 8.43 (1H, d, NH), 7.85 (1H, s, H-4), 7.64 (1H, s, H-7), 7.44 (1H, d, *J* = 8.00 Hz, H-6'), 7.40-7.32 (3H, m, H-2', H-2'', H-6''), 7.29 (1H, t, H-5'), 6.98-6.85 (3H, m, H-4', H-3'', H-5''), 5.92 (1H, s, CH_2), 3.76 (3H, s, OCH_3), 2.41 (6H, s, $2CH_3$). ^{13}C-NMR (DMSO-d_6) δ: 154.5 (CO), 152.5 (C), 144.5 (C), 140.3 (C), 137.3 (C), 136.7 (C), 133.5 (C), 132.5 (C), 131.8 (C), 129.2 (CH), 120.7 (CH), 120.0 (2CH), 118.1 (CH), 117.5 (CH), 116.8 (CH), 113.9 (2CH), 109.7 (CH), 55.1 (OCH_3), 50.8 (CH_2), 20.4 (CH_3), 19.8 (CH_3). LC/MS *m/z*: 402 $[M+H]^+$.

1-(3-((5,6-dimethyl-1H-benzo[d][1,2,3]triazol-1-yl)methyl)phenyl)-3-(3,4,5-trimethoxyphenyl)urea (71b)

Compound **71b** was obtained, in 43% total yield; mp: 206-208 °C; TLC (petroleum ether/ethyl acetate = 6/4) R_f: 0.05. ^{1}H NMR (400 MHz, DMSO-d$_6$) δ: 8.64 (1H, s, NH), 8.53 (1H, s, NH), 7.80 (1H, s, H-4), 7.60 (1H, s, H-7), 7.30-7.40 (2H, m, H-2', H-6'), 7.24 (1H, t, H-5'), 6.88 (1H, d, *J* = 7.2 Hz, H-4'), 6.77 (2H, s, H-2'', H-6''), 5.87 (2H, s, CH_2), 3.74 (6H, s, CH_3), 3.60 (3H, s, CH_3), 2.35 (6H, s, $2CH_3$). ^{13}C-NMR (DMSO-d_6) δ: 152.8 (CO), 152.4 (C), 144.5 (C), 150.0 (C), 139.9 (C), 137.4 (C), 136.7 (C), 133.6 (C), 135.7 (C), 135.6 (C), 133.5 (C), 132.5 (C), 131.7 (C), 129.2 (CH), 120.9 (CH), 118.1 (CH), 117.8 (CH), 117.1 (CH), 109.8 (CH), 96.0 (CH), 60.1 (OCH_3), 55.7 ($2OCH_3$), 50.8 (CH_2), 20.4 (CH_3), 19.8 (CH_3). LC/MS *m/z*: 462 $[M+H]^+$.

1-(3-((5,6-dimethyl-1H-benzo[d][1,2,3]triazol-1-yl)methyl)phenyl)-3-(4-fluorophenyl)urea (72b)

Compound **72b** was obtained, in 38% total yield; mp: 245-247 °C; TLC (petroleum ether/ethyl acetate = 6/4) R_f: 0.18. ^{1}H NMR (400 MHz, DMSO-d_6) δ: 8.68 (1H, s, NH), 8.60 (1H, s, NH), 7.80 (1H, s, H-4), 7.58 (1H, s, H-7), 7.48-7.34 (3H, d, H-6', H-2'', H-6''), 7.30 (1H, s, H-2'), 7.25 (1H, t, H-5'), 7.10 (2H, t, H-3'', H-5''), 6.90 (1H, d, *J* = 7.6 Hz, H-4'), 5.87 (1H, s, CH_2), 2.35 (6H, s, $2CH_3$). ^{13}C-NMR (DMSO-d_6) δ: 157.3 (C-F), 152.5 (CO), 140.1 (C), 137.4 (C), 136.7 (C), 135.8 (C), 133.5 (C), 131.8 (C), 129.2 (CH), 120.9 (CH), 120.0 (CH), 119.9 (CH), 118.1 (CH), 117.7 (CH), 116.9 (CH), 115.3 (CH), 115.1 (CH), 109.7 (CH), 50.8 (CH_2), 20.4 (CH_3), 19.8 (CH_3). LC/MS *m/z*: 390 $[M+H]^+$.

1-(3-((5,6-dimethyl-1H-benzo[d][1,2,3]triazol-1-yl)methyl)phenyl)-3-(4-chlorophenyl)urea (73b)

Compound **73b** was obtained, in 17% total yield; mp: 249-251 °C; TLC (petroleum ether/ethyl acetate = 6/4) R_f: 0.20. ^{1}H NMR (400 MHz, DMSO-d_6) δ: 8.72 (2H, s, 2NH), 7.80 (1H, s, H-4), 7.58 (1H, s, H-7), 7.45 (2H, d, *J* = 8.8 Hz, H-3'', H-5''), 7.40 (1H, d, *J* = 8.00 Hz, H-6'), 7.35-7.28 (3H, m, H-2', H-2'', H-6''), 7.26 (1H, t, H-5'), 6.91 (1H, d, *J* = 7.6 Hz, H-4'), 5.87 (1H, s, CH_2), 2.35 (6H, s, $2CH_3$). ^{13}C-NMR (DMSO-d_6) δ: 152.3 (CO), 144.5 (C), 139.9 (C), 138.5 (C), 137.4 (C), 136.7 (C), 133.5 (C), 131.7 (C), 129.2 (CH), 128.6 (2CH), 125.4 (CH), 121.0 (CH), 119.7 (2CH), 118.1 (CH), 117.8 (CH), 117.0 (CH), 109.7 (CH), 50.7 (CH_2), 20.4 (CH_3), 19.8 (CH_3). LC/MS *m/z*: 406 $[M+H]^+$.

1-(3-((5,6-dimethyl-1H-benzo[d][1,2,3]triazol-1-yl)methyl)phenyl)-3-(4-(trifluoromethyl)phenyl)urea (74b)

Compound **74b** was obtained, in 33% total yield; mp: 290-292 °C; TLC (petroleum ether/ethyl acetate = 6/4) R_f : 0.18. ^{1}H NMR (400 MHz, DMSO-*d6*) δ: 9.03 (1H, s, NH), 8.85 (1H, s, NH), 7.80 (1H, s, H-4), 7.62 (4H, s, H-2'', H-3'', H-5'', H-6''), 7.59 (1H, s, H-7), 7.41 (1H, d, *J* = 8.4 Hz, H-6'), 7.33 (1H, s, H-2'), 7.27 (1H, t, H-5'), 6.93 (1H, d, *J* = 7.2 Hz, H-4'), 5.88 (1H, s, CH_2), 2.35 (6H, s, $2CH_3$). ^{13}C-NMR (DMSO-*d6*) δ: 152.1 (CO), 144.5 (C), 143.3 (C), 139.7 (2C), 137.4 (C), 136.8 (2C), 133.5 (C), 131.8 (C), 129.2 (CH), 126.0 (CH), 121.3 (CH), 118.1 (CH), 117.8 (4CH), 117.2 (CH), 109.7 (CH), 50.7 (CH_2), 20.4 (CH_3), 19.8 (CH_3). LC/MS *m/z*: 440 $[M+H]^+$.

N-(4-(5,6-dimethyl-2H-benzo[d][1,2,3]triazol-2-yl)phenyl)acetamide (75b)

Compound **75b** was obtained, in 89% total yield; mp: 166-168 °C; TLC (petroleum ether /ethyl acetate 1:1): R_f : 0.22. ^{1}H NMR (400 MHz, DMSO-*d6*) δ: 9.98 (1H, s, NH), 7.64 (1H, s, H-4,7), 7.53 (2H, d, *J* = 8.8, H-2′,6′), 7.27 (2H, d, *J* = 8.4, H-3′,5′), 5.80 (2H, s, CH_2), 2.34 (6H, s, $2CH_3$), 2.01 (3H, s, CH_3). ^{13}C-NMR (DMSO-*d6*): 168.9 (C), 145.5 (2C), 136.6 (2C), 135.2 (C), 130.9 (C), 128.8 (2CH), 124.1 (CH), 121.5 (2CH), 61.6 (CH_2), 24.0 (CH_3), 18.8 ($2CH_3$). LC/MS *m/z* 295 $[M+H]^+$.

N-(4-(5,6-dimethyl-2H-benzo[d][1,2,3]triazol-2-yl)phenyl)propionamide (76b)

Compound **76b** was obtained, in 82% total yield; mp: 177-179 °C; TLC (petroleum ether /ethyl acetate 6:4): R_f : 0.29. ^{1}H NMR (400 MHz, DMSO-*d6*) δ: 9.91 (1H, s, NH), 7.64 (1H, s, H-4,7), 7.56 (2H, d, *J* = 7.8, H-2′,6′), 7.29 (2H, d, *J* = 7.8, H-3′,5′), 5.81 (2H, s, CH_2), 2.46 (2H, q, *CH_2*CH_3), 2.35 (6H, s, $2CH_3$), 1.06 (3H, t, CH_2*CH_3*). ^{13}C-NMR (DMSO-*d6*): 168.9 (C), 145.5 (2C), 136.6 (2C), 135.2 (C), 130.9 (C), 128.8 (2CH), 124.1 (CH), 121.5 (2CH), 61.6 (CH_2), 30.0 (CH_2), 18.8 ($2CH_3$), 10.4 (CH_3). LC/MS *m/z* 309 $[M+H]^+$.

***N-(4-(5,6-dimethyl-1H-benzo[d][1,2,3]triazol-1-yl)phenyl)acetamide* (77b)**

Compound **77b** was obtained, in 90% total yield; mp: 205-207 °C; TLC (petroleum ether /ethyl acetate 4:6): R_f : 0.24. ^{1}H NMR (400 MHz, DMSO-*d6*) δ: 9.96 (1H, s, NH), 7.77 (1H, s, H-4), 7.58 (1H, s, H-7),7.51 (2H, d, *J* = 8.0, H-2′,6′), 7.24 (2H, d, *J* = 8.4, H-3′,5′), 5.81 (2H, s, CH_2), 2.34 (6H, s, $2CH_3$), 2.00 (3H, s, CH_3). ^{13}C-NMR (DMSO-*d6*): 168.9 (C), 145.6 (C), 136.6 (2C), 135.2 (C), 132.8 (C), 130.9 (C), 128.8 (2CH), 124.1 (CH), 121.5 (2CH), 61.6 (CH_2), 24.0 (CH_3), 18.8 ($2CH_3$). LC/MS *m/z* 295 $[M+H]^+$.

N-(4-(5,6-dimethyl-1H-benzo[d][1,2,3]triazol-1-yl)phenyl)propionamide (78b)

Compound **78b** was obtained, in 30% total yield; mp: 218-220 °C; TLC (petroleum ether /ethyl acetate 1:1): R_f : 0.32. ^{1}H NMR (400 MHz, DMSO-*d6*) δ: 9.89 (1H, s, NH), 7.78 (1H, s, H-4), 7.58 (1H, s, H-7), 7.54 (2H, d, *J* = 8.6, H-2′,6′), 7.24 (2H, d, *J* = 8.6, H-3′,5′), 5.81 (2H, s, CH_2), 2.34 (6H, s, $2CH_3$), 2.32 (2H, q, CH_2CH_3), 1.04 (3H, t, CH_2CH_3). ^{13}C-NMR (DMSO-*d6*): 168.9 (C), 145.6 (C), 136.6 (2C), 135.2 (C), 132.8 (C), 130.9 (C), 128.8 (2CH), 124.1 (CH), 121.5 (2CH), 61.6 (CH_2), 32.0 (CH_2), 18.8 ($2CH_3$). 10.4 (CH_3). LC/MS *m/z* 309 $[M+H]^+$.

N-(4-((1H-benzo[d][1,2,39]triazol-1-yl)methyl)phenyl)acetamide (79c)

Compound **79c** was obtained, in 75% total yield; mp: 187-189 °C; TLC (petroleum ether /ethyl acetate 6:4): R_f : 0.10. ^{1}H NMR (400 MHz, DMSO-*d6*) δ:9.97 (1H, s, NH), 8,04 (1H, d, *J* = 8.2, H-4), 7,82 (1H, d, *J* = 8.2, H-7), 7.53-7.48 (3H, m, H-3′,5′,H-5), 7.40 (1H, t, H-6), 7.28 (2H, d, *J* = 8.6, H-2′,6′), 5.90 (2H, s, CH_2), 2.00 (3H, s, CH_3).^{13}C-NMR (DMSO-*d6*) δ: 168.9 (CO), 145.5 (C), 132.8 (C), 131.8 (C), 135.5 (C), 129.2 (2CH), 126.2 (2CH), 121.5 (2CH), 119.6 (CH), 110.0 (CH), 54.7 (CH_2), 24.0 (CH_3). LC/MS *m/z* 267$[M+H]^+$.

N-(4-((1H-benzo[d][1,2,3]triazol-1-yl)methyl)phenyl)propionamide (80c)

Compound **80c** was obtained, in 73% total yield; mp: 192-195 °C; TLC (petroleum ether /ethyl acetate 6:4): R_f : 0.32. ^{1}H NMR (400 MHz, DMSO-*d6*) δ:9.90 (1H, s, NH), 8,04 (1H, d, *J* = 8.0, H-4), 7,82 (1H, d, *J* = 8.0, H-7), 7.59-7.48 (3H, m, H-3′,5′, H-5), 7.36 (1H, t, H-6), 7.28 (2H, d, *J* = 8.0, H-2′,6′), 5.90 (2H, s, CH_2), 2.31 (2H, q, $\underline{CH_2}CH_3$), 1.04 (3H, t, $CH_2\underline{CH_3}$). ^{13}C-NMR (DMSO-*d6*): 168.8 (CO), 145.5 (C), 132.8 (C), 131.8 (C), 135.5 (C), 129.2 (2CH), 126.2 (2CH), 121.5 (2CH), 119.6 (CH), 110.0 (CH), 54.7 (CH_2), 30.6 (CH_2), 10.0 (CH_3). LC/MS *m/z* 281 $[M+H]^+$.

***N-(4-((5,6-difluoro-1H-benzo[d][1,2,3]triazol-1-yl)methyl)phenyl)acetamide* (81d)**

Compound **81d** was obtained, in 50% total yield; mp: 242-243 °C; TLC (petroleum ether /ethyl acetate 6:4): R_f : 0.10. ^{1}H NMR (400 MHz, DMSO-*d6*) δ: 9.97 (H, s, NH), 8.21 (H, t, H-4), 8.13 (H, t, H-7), 7.53 (2H, d, *J* = 8.0 Hz, H-3′, 5′), 7.31 (2H, d, *J* = 8.0 Hz, H-2′, 6′), 5.87 (2H, s, CH_2), 2.00 (3H, s, CH_3).^{13}C-NMR (DMSO-*d6*): δ 168.9 (C), 149.5 (2C), 145.5 (C), 135.5 (C), 132.8 (C), 131.8 (C), 129.2 (2CH), 121.5 (2CH), 117.1 (2CH), 54.7 (CH_2), 24.0 (CH_3). LC/MS: *m/z* 303 $[M+H]^+$.

N-(4-((5,6-difluoro-1H-benzo[d][1,2,3]triazol-1-yl)methyl)phenyl)propionamide (82d)

Compound **82d** was obtained, in 22% total yield; mp: 240-242 °C; TLC (petroleum ether /ethyl acetate 6:4): R_f : 0.24. ^{1}H NMR (400 MHz, DMSO-*d6*) δ: 9.90 (H, s, NH), 8.19 (H, t, H-4), 8.10 (H, t, H-7), 7.55 (2H, d, *J* = 8.0 Hz, H-3′, 5′), 7.31 (2H, d, *J* = 8.0 Hz, H-2′, 6′), 5.87 (2H, s, CH_2), 2.28 (2H, q, CH_2), 1.05 (3H, s, CH_3).^{13}C-NMR (DMSO-*d6*): δ 172. 0 (C), 149.5 (2C), 145.5 (C), 135.5 (C), 132.8 (C), 131.8 (C), 129.2 (2CH), 121.5 (2CH), 117.1 (2CH), 54.7 (CH_2), 30.6 (CH_2), 10.0 (CH_3). LC/MS: *m/z* 317 $[M+H]^+$.

N-(4-((5,6-dimethyl-1H-benzo[d][1,2,3]triazol-1-yl)methyl)phenyl)-4-methoxylbenzamide (83b)

Compound **83b** was obtained, in 53% total yield; mp: 196-198 °C; TLC (petroleum ether/ethyl acetate = 1/1) R_f : 0.30. ^{1}H NMR (400 MHz, DMSO-d_6) δ: 10.13 (1H, s, NH), 7.93 (2H, d, *J* = 8.6 Hz, H-2″, 6″), 7.80 (1H, s, H-4), 7.73 (2H, d, *J*=8.2 Hz, H-3′, 5′), 7.61 (1H, s, H-7), 7.30 (2H, d, *J*=8.2 Hz, H-2′, 6′), 7.05 (2H, d, *J*=8.4 Hz, H-3″, 5″), 5.86 (2H, s, CH_2), 3.83 (3H, s, OCH_3), 2.36 (6H, s, 2CH_3). ^{13}C-NMR (DMSO-d_6) δ: 164.9 (CO), 161.9 (C), 144.6 (C), 139.7 (C), 137.3 (C), 136.4 (C), 133.5 (C), 131.7 (C), 129.6 (CH), 129.3 (CH), 128.9 (CH), 126.7 (C), 122.6 (CH), 119.9 (CH), 119.2 (CH), 118.1 (CH), 113.7 (CH), 113.5 (CH), 109.7 (CH), 55.4 (OCH_3), 50.8 (CH_2), 20.1 (2CH_3). LC/MS *m/z* 387 [M+H]+.

N-(4-((5,6-dimethyl-1H-benzo[d][1,2,3]triazol-1-yl)methyl)phenyl)-4-nitrobenzamide (84b)

Compound **84b** was obtained, in 34% total yield; mp: 192-194 °C; TLC (petroleum ether/ethyl acetate = 1/1) R_f : 0.28. ^{1}H NMR (400 MHz, DMSO-d_6) δ: 10.19 (1H, s, NH), 8.21 (1H, s, H-4), 7.76 (1H, s, H-7), 7.58-7.60 (6H, m, H-3′,5′,2″,3″,5″,6″), 7.25 (2H, d, *J*=8.2 Hz, H-2′,6′), 5.81 (2H, s, CH_2), 2.33 (6H, s, 2CH_3). ^{13}C-NMR (DMSO-d_6) δ: 163.9 (CO), 149.1 (C), 144.6 (C), 140.4 (C), 139.1 (C), 137.4 (C), 136.7 (C), 133.5 (C), 131.7 (C), 129.2 (2CH), 129.1 (CH), 123.5 (CH), 123.4 (CH), 122.7 (CH), 120.0 (CH), 119.3 (CH), 118.1 (CH), 109.7 (CH), 50.7 (CH_2), 20.1 (2CH_3). LC/MS *m/z* 402 [M+H]+.

N-(4-((1H-benzo[d][1,2,3]triazol-1-yl)methyl)phenyl)-4-nitrobenzamide (85c)

Compound **85c** was obtained, in 34% total yield; mp: 245-246 °C; TLC (petroleum ether/ethyl acetate = 4/6) R_f : 0.68. ^{1}H NMR (400 MHz, DMSO-d_6) δ: 10.62 (1H, s, NH), 8.36 (2H, d, *J* = 7.8, H-3″, 5″), 8.15 (2H, d, *J* = 7.8, H-2″, 6″), 8.06 (1H, d, *J* = 7.6, H-4), 7.85 (1H, d, *J* = 7.6, H-7), 7.75 (2H, d, *J* = 7.8, H-3′, 5′), 7.55 (1H, t, H-5), 7.44-7.36 (3H, m, H-6, 2′, 6′), 5.97 (2H, s, CH_2). ^{13}C-NMR (DMSO-d_6) δ: 163.9 (CO), 149.1 (C), 144.6 (C), 140.4 (C), 139.1 (C), 133.5 (C), 131.7 (C), 129.2 (2CH), 129.1 (CH), 126.2 (2CH), 123.5 (CH), 123.3 (CH), 122.7 (CH), 120.0 (CH), 119.3 (CH), 118.1 (CH), 109.7 (CH), 50.7 (CH_2). LC/MS *m/z* 374, 375 [M+H]+.

N-(4-((1H-benzo[d][1,2,3]triazol-1-yl)methyl)phenyl)-4-methoxybenzamide (86c)

Compound **86c** was obtained, in 41% total yield; mp: 172-174 °C; TLC (petroleum ether/ethyl acetate = 6/4) R_f : 0.21. ^{1}H NMR (400 MHz, DMSO-d_6) δ: 10.14 (1H, s, NH), 8.06 (1H, d, *J* = 8.2, H-4), 7.94 (2H, d, *J* = 8.4, H-2″,6″) 7.85 (1H, d, *J* = 9.0, H-7), 7.75 (2H, d, *J* = 7.6, H-3′,5′), 7.56 (1H, t, H-5), 7.44-7.33 (3H, m, H-6 e 2′,6′), 7.04 (2H, d, *J* = 8.0, H-3″,5″), 5.95 (2H, s, CH_2), 3.84 (3H, s, OCH_3). ^{13}C-NMR (DMSO-d_6) δ: 163.9 (CO), 154.1 (C), 144.6 (C), 140.4 (C), 139.1 (C), 137.4 (C), 136.7 (C), 133.5 (C), 131.7 (C), 129.2 (2CH), 129.1 (CH), 126.2 (2CH), 123.5 (CH), 123.3 (CH), 122.7 (CH), 120.0 (CH), 119.3 (CH), 118.1 (CH), 109.7 (CH), 60.1 (OCH_3), 50.7 (CH_2). LC/MS *m/z* 359 [M+H]+.

N-(4-((1H-benzo[d][1,2,3]triazol-1-yl)methyl)phenyl)-3, 4, 5-trimethoxybenzamide (87c)

Compound **87c** was obtained, in 54% total yield; mp: 147-149 °C; TLC (petroleum ether/ethyl acetate = 4/6) R_f : 0.35. ^{1}H NMR (400 MHz, DMSO-d_6) δ: 10.17 (1H, s, NH), 8.05 (1H, d, *J* = 8.0, H-4), 7.95 (1H, s, H-2″), 7.86 (1H, d, *J* = 8.0, H-7), 7.70 (2H, d, *J* = 8.0, H-3′,5′), 7.53 (1H, t, H-5), 7.40-7.26 (3H, m, H-6, 2′,6′), 7.24 (1H, s, H-6″), 5.95 (2H, s, CH_2), 3.84 (6H, s, 2OCH_3), 3.71 (3H, s, OCH_3).^{13}C-NMR (DMSO-d_6) δ: 165.0 (CO), 152.6 (2CH), 144.3 (C), 140.3 (C), 139.5 (C), 135.8 (C), 129.8 (C), 129.1 (CH), 127.3 (C), 126.1 (CH), 126.3 (CH), 123.2 (C), 120.6 (CH), 120.4 (C), 119.7 (CH), 112.5 (CH), 105.3 (2CH), 60.1 (OCH_3), 56.1 (2OCH_3), 51.2 (CH_2). LC/MS *m/z* 419 [M+H]+.

N-(4-((1H-benzo[d][1,2,3]triazol-1-yl)methyl)phenyl)-4-(trifluoromethoxy)benzamide (88c)

Compound **88c** was obtained, in 63% total yield; mp: 229-231 °C; TLC (petroleum ether/ethyl acetate = 6/4) R_f : 0.55. ^{1}H NMR (400 MHz, DMSO-d_6) δ: 10.52 (1H, s, NH), 8.14-8.00 (4H, m, H arom), 7.94-7.83 (4H, m, H arom) 7.80-7.72 (2H, m, H arom), 7.42-7.35 (2H, m, H arom), 5.96 (2H, s, CH_2). ^{13}C-NMR (DMSO-d_6) δ: 163.9 (CO), 154.1 (C), 144.6 (C), 140.4 (C), 139.1 (C), 137.4 (C), 136.7 (C), 133.5 (C), 131.7 (C), 129.7 (C), 129.2 (2CH), 129.1 (CH), 126.2 (2CH), 123.5 (CH), 123.3 (CH), 122.7 (CH), 120.0 (CH), 119.3 (CH), 118.1 (CH), 109.7 (CH), 50.7 (CH_2). LC/MS *m/z* 413 [M+H]+.

N-(4-((5,6-difluoro-1H-benzo[d][1,2,3]triazol-1-yl)methyl)phenyl)-4-methylbenzamide (89d)

Compound **89d** was obtained, in 12% total yield; mp: 297-299 °C; TLC (petrol ether/ethyl acetate = 7/3) R_f : 0.47. ^{1}H NMR (400 MHz, DMSO-d_6) δ: 10.70 (H, s, NH), 8.28-8.10 (2H, m, H-4, 7), 7.94 (2H, t, H-3″, 5″), 7.70 (2H, d, *J* = 7.8 Hz, H-3′, 5′), 7.35 (2H, d, *J* = 6.6 Hz, H-2′, 6′), 7.35 (2H, d, *J* = 6.6 Hz, H-2″, 6″), 6.00 (2H, s, CH_2). ^{13}C-NMR (DMSO-d_6): δ 164.7 (C), 149.5 (2C), 145.5 (C), 139.3 (C), 134.9 (C), 132.8 (C), 132.3 (C), 131.8 (C), 129.3 (2CH), 129.2 (2CH), 127.5 (2CH), 121.5 (2CH), 54.7 (CH_2). LC/MS: *m/z* 379 [M+H]+.

N-(4-((5,6-difluoro-1H-benzo[d][1,2,3]triazol-1-yl)methyl)phenyl)-4-nitrobenzamide (90d)

Compound **90d** was obtained, in 53% total yield; mp: 240-242 °C; TLC (chloroform/methanol = 99.5/0.5) R_f : 0.67. ^{1}H NMR (400 MHz, DMSO-d_6) δ: 10.67 (H, s, NH), 8.35 (2H, d, *J* = 6.6 Hz, H-3″, 5″), 8.34-8.19 (4H, m, H-4, 7, 2″, 6″), 7.81 (2H, d, *J* = 6.6 Hz, H-3′, 5′), 7.42 (2H, d, *J* = 6.6 Hz, H-2′, 6′), 5.99 (2H, s, CH_2). ^{13}C-NMR (DMSO-d_6): δ 164.7 (C), 151.3 (C), 149.5 (2C), 145.5 (C), 136.8 (C), 134.9 (C), 132.8 (C), 131.8 (C), 129.6 (2CH), 129.2 (2CH), 124.0 (2CH), 121.5 (2CH), 117.1 (2CH) 57.7 (CH_2). LC/MS: *m/z* 410 [M+H]+.

N-(4-((5,6-difluoro-1H-benzo[d][1,2,3]triazol-1-yl)methyl)phenyl)-3, 4, 5-trimethoxybenzamide (91d)

Compound **91d** was obtained, in 53% total yield; mp: 134-136 °C; TLC (chloroform/methanol = 99.5/0.5) R_f : 0.46. ^{1}H NMR (400 MHz, DMSO-*d*$_6$) *δ*: 10.16 (H, s, NH), 8.27 (H, td, H-4), 8.19 (H, td, H-7), 7.70 (2H, d, *J* = 8.4 Hz, H-3′, 5′), 7.38 (2H, d, *J* = 8.6 Hz, H-2′, 6′), 7.24 (2H, s, H-2″, 6″), 5.91 (2H, s, CH_2). ^{13}C-NMR (DMSO-*d*$_6$): *δ* 164.7 (C), 153.2 (2C), 149.5 (2C), 145.5 (C), 142.6 (C), 134.9 (C), 132.8 (C), 131.8 (C), 124.1 (C), 129.2 (2CH), 121.5 (2CH), 117.1 (2CH), 106.6 (2CH), 60.8 (CH_3), 56.1 ($2CH_3$), 54.7 (CH_2). LC/MS: *m/z* 455 [M+H]$^+$.

N-(4-((5,6-difluoro-1H-benzo[d][1,2,3]triazol-1-yl)methyl)phenyl)- 4-fluorobenzamide (92d)

Compound **92d** was obtained, in 50% total yield; mp: 241-242 °C; TLC (petroleum ether/ethyl acetate = 7/3) R_f : 0.21. ^{1}H NMR (400 MHz, DMSO-*d*$_6$) *δ*: 10.33 (H, s, NH), 8.30-8.05 (2H, m, H-4, 7), 7.98 (2H, t, H-3″, 5″), 7.74 (2H, d, *J* = 7.8 Hz, H-3′, 5′), 7.46-7.30 (4H, m, H-2′, 6′, 2″, 6″), 5.91 (2H, s, CH_2). ^{13}C-NMR (DMSO-*d*$_6$): *δ* 166.3 (C), 149.5 (2C), 145.5 (C), 134.9 (C), 132.8 (C), 131.8 (C), 129.8 (C), 129.2 (2CH), 129.1 (2CH), 121.5 (2CH), 117.1 (2CH) 115.6 (2CH), 54.7 (CH_2). LC/MS: *m/z* 383[M+H]$^+$.

N-(4-((5,6-difluoro-1H-benzo[d][1,2,3]triazol-1-yl)methyl)phenyl)- 4-chlorobenzamide (93d)

Compound **93d** was obtained, in 20% total yield; mp: 263-264 °C; TLC (chloroform/methanol = 99/1)) R_f : 0.35. ^{1}H NMR (400 MHz, DMSO-*d*$_6$) *δ*: 10.70 (H, s, NH), 8.28-8.10 (2H, m, H-4, 7), 8.08 (2H, t, H-3″, 5″), 7.70 (2H, d, *J* = 7.8 Hz, H-3′, 5′), 7.42 (2H, d, *J* = 6.6 Hz, H-2′, 6′), 7.35 (2H, d, *J* = 6.6 Hz, H-2″, 6″), 6.00 (2H, s, CH_2). ^{13}C-NMR (DMSO-*d*$_6$): *δ* 164.7 (C), 149.5 (2C), 145.5 (C), 137.7 (C), 134.9 (C), 132.8 (C), 132.3 (C), 131.8 (C), 130.1 (2CH), 129.2 (2CH), 128.2 (2CH), 121.5 (2CH), 54.7 (CH_2). LC/MS: *m/z* 400 [M+H]$^+$.

N-(4-((5,6-difluoro-1H-benzo[d][1,2,3]triazol-1-yl)methyl)phenyl)- 4-bromobenzamide (94d)

Compound **94d** was obtained, in 20% total yield; mp: 235-236 °C; TLC (petroleum ether/ethyl acetate = 7/3) R_f : 0.42. ^{1}H NMR (400 MHz, DMSO-*d*$_6$) *δ*: 10.70 (H, s, NH), 8.30-8.10 (2H, m, H-4, 7), 8.10 (2H, t, H-3″, 5″), 7.72 (2H, d, *J* = 7.8 Hz, H-3′, 5′), 7.40 (2H, d, *J* = 6.6 Hz, H-2′, 6′), 7.38 (2H, d, *J* = 6.6 Hz, H-2″, 6″), 5.98 (2H, s, CH_2). ^{13}C-NMR (DMSO-*d*$_6$): *δ* 164.7 (C), 149.5 (2C), 145.5 (C), 134.9 (C),133.2 (C), 132.8 (C), 131.8 (C), 126.5 (C), 131.7 (2CH), 129.7 (2CH), 129.2 (2CH), 121.5 (2CH), 117.1 (2CH) 54.7 (CH_2). LC/MS: *m/z* 444 [M+H]$^+$.

1-(4-((5,6-dichloro-1H-benzo[d][1,2,3]triazol-1-yl)methyl)phenyl)-3-ethylurea (95a)

Compound **95a** was obtained, in 80% total yield; mp: 211-212 °C; TLC (petroleum ether/ethyl acetate = 6/4) R_f : 0.15. ^{1}H NMR (400 MHz, DMSO-*d*$_6$) *δ*: 8.47 (2H, s, H-4 and *NH*-Ph), 8.36 (1H, s, H-7), :7.36 (2H, d, *J* = 7.2 Hz, H-3′,5′), 7.26 (2H, d, *J* = 7.2 Hz, H-2′,6′), 6.09 (H, t, *NH*-CH_2), 5.86 (2H, s, CH_2), 3.08 (2H, q, CH_2), 1.02 (3H, t, CH_3). ^{13}C-NMR (DMSO-*d*$_6$) *δ*: 155.0 (CO), 144.3 (C), 140.7 (C), 131.8 (C), 130.6 (C), 128.6 (2CH), 127.5 (C), 127.1 (C), 120.6 (CH), 117.7 (2CH), 112.5 (CH), 51.0 (CH_2-Ph), 33.9 (CH_2), 15.4 (CH_3). LC/MS *m/z*: 364, 366 [M+H]$^+$.

1-(4-((5,6-dichloro-1H-benzo[d][1,2,3]triazol-1-yl)methyl)phenyl)-3-propylurea (96a)

Compound **96a** was obtained, in 12% total yield; mp: 213-214 °C; TLC (petroleum ether/ethyl acetate = 6/4) R_f : 0.21. ^{1}H NMR (400 MHz, DMSO-*d*$_6$) *δ*: 8.46 (2H, s, H-4 and *NH*-Ph), 8.36 (1H, s, H-7), 7.36 (2H, d, *J* = 8.0 Hz, H-3′,5′), 7.25 (2H, d, *J* = 8.0 Hz, H-2′,6′), 6.12 (H, t, *NH*-CH_2), 5.86 (2H, s, CH_2), 3.01 (2H, q, *CH*$_2$-NH), 1.41 (2H, q, *CH*$_2$-CH_3), 0.85 (3H, t, CH_3). ^{13}C-NMR (DMSO-*d*$_6$) *δ* 155.0 (CO), 144.3 (C), 140.7 (C), 131.8 (C), 130.6 (C), 128.5 (2CH), 127.5 (C), 127.1 (C), 120.5 (CH), 117.7 (2CH), 112.5 (CH), 51.0 (CH_2-Ph), 40.8 (CH_2), 22.9 (CH_2), 11.3 (CH_3). LC/MS *m/z*: 378, 380 [M+H]$^+$.

1-butyl-3-(4-((5,6-dichloro-1H-benzo[d][1,2,3]triazol-1-yl)methyl)phenyl)urea (97a)

Compound **97a** was obtained, in 20% total yield; mp: 216-217 °C; TLC (petroleum ether/ethyl acetate = 6/4) R_f : 0.37. ^{1}H NMR (400 MHz, DMSO-*d*$_6$) *δ*: 8.52 (H, s, H-4), 8.49 (7H, s, *NH*-Ph), 8.42 (H, s, H-7), 7.41 (2H, d, *J* = 8.4 Hz, H-3′,5′), 7.31 (2H, d, *J* = 8.4 Hz, H-2′,6′), 6.15 (H, t, *NH*-CH_2), 5.92 (2H, s, CH_2), 3.10 (2H, q, *CH*$_2$-NH), 1.46-1.38 (2H, m, *CH*$_2$-CH_2), 1.37-1.31 (2H, m, *CH*$_2$-CH_3), 0.92 (3H, t, CH_3). ^{13}C-NMR (DMSO-*d*$_6$) *δ*: 155.0 (CO), 144.3 (C), 140.7 (C), 131.8 (C), 130.6 (C), 128.6 (2CH), 127.5 (C), 127.1 (C), 120.6 (CH), 117.6 (2CH), 112.5 (CH), 51.0 (CH_2-Ph), 38.6 (CH_2), 31.8 (CH_2), 19.5 (CH_2), 13.6 (CH_3). LC/MS *m/z*: 392, 394 [M+H]$^+$.

1-cyclohexyl-3-(4-((5,6-dichloro-1H-benzo[d][1,2,3]triazol-1-yl)methyl)phenyl)urea (98a)

Compound **98a** was obtained, in 80% total yield; mp: 130-132 °C; TLC (petroleum ether/ethyl acetate = 6/4) R_f : 0.46. ^{1}H NMR (400 MHz, DMSO-*d*$_6$) *δ*: 8.47 (H, s, H-4), 8.35 (H, s, H-7), 8.36 (H, s, *NH*-Ph), 7.34 (2H, d, *J* = 8 Hz, H-3′,5′), 7.25 (2H, d, *J* = 8 Hz, H-2′,6′), 6.08 (H, d, *NH*-CH), 5.86 (2H, s, CH_2), 3.35-3.20 (H, m, *CH*-NH), 1.78-1.49 (6H, m, $3CH_2$), 1.30-1.16 (4H, m, $2CH_2$). ^{13}C-NMR (DMSO-*d*$_6$) *δ*: 156.6 (CO), 154.2 (C), 144.3 (C), 140.6 (C), 131.8 (C), 130.6 (C), 128.6 (2CH), 127.3 (C), 120.7 (CH), 117.6 (2CH), 112.5 (CH), 51.0 (CH_2Ph), 47.5 (*CH*-NH), 33.3 ($2CH_2$), 32.9 (CH_2), 25.3 (CH_2), 24.4 (CH_2). LC/MS *m/z*: 418, 421 [M+H]$^+$.

1-(4-((5,6-dimethyl-1H-benzo[d][1,2,3]triazol-1-yl)methyl)phenyl)-3-propylurea (99b)

Compound **99b** was obtained, in 63% total yield; mp: 190-192 °C; TLC (petroleum ether/ethyl acetate = 6/4) R_f : 0.29. ^{1}H NMR (400 MHz, DMSO-*d*$_6$) *δ*: 8.42 (1H, s, *NH*-Ph), 7.78 (1H, s, H-4), 7.57 (1H, s, H-7), 7.33 (2H, d, *J* = 8.0, H-2′, 6′), 7.17 (2H, d, *J* = 8.0, H-3′, 5′), 6.14-6.05 (1H, m, *NH*-CH_2), 5.77 (2H, s, CH_2), 3.10-2.90 (2H, m, *CH*$_2$-NH), 2.31 (6H,

s, 2CH$_3$), 1.80-1.40 (4H, m, 2CH$_2$), 1.42-1.30 (2H, q, *CH*$_2$CH$_3$), 0.85 (3H, t, CH$_2$*CH*$_3$). ^{13}C-NMR (DMSO-*d*$_6$) δ:155.0 (CO), 144.3 (C), 140.7 (C), 134.6 (C), 133.8 (C), 128.5 (2CH), 127.5 (C), 127.1 (C), 120.5 (CH), 116.7 (2CH), 112.5 (CH), 51.0 (CH$_2$-Ph), 40.8 (CH$_2$), 22.9 (CH$_2$), 11.3 (CH$_3$). LC/MS *m/z* 338 [M+H]$^+$.

1-cyclohexyl-3-(4-((5,6-dimethyl-1H-benzo[d][1,2,3]triazol-1-yl)methyl)phenyl)urea (100b)

Compound **100b** was obtained, in 48% total yield; mp: 216-218 °C; TLC (petroleum ether/ethyl acetate = 6/4) R$_f$: 0.23. ^{1}H NMR (400 MHz, DMSO-*d*$_6$) δ: 8.29 (1H, s, *NH*-Ph), 7.74 (1H, s, H-4), 7.55 (1H, s, H-7), 7.28 (2H, d, *J* = 8.2, H-2′, 6′), 7.15 (2H, d, *J* = 8.0, H-3′, 5′), 6.01 (1H, d, *J* = 7.6, *NH*-CH), 5.74 (2H, s, CH$_2$), 3.51-3.25 (1H, m, CH), 2.31 (6H, s, 2CH$_3$), 1.80-1.40 (4H, m, 2CH$_2$), 1.35-1.00 (6H, m, 3CH$_2$). ^{13}C-NMR (DMSO-*d*$_6$) δ156.6 (CO), 154.2 (C), 144.3 (C), 140.6 (C), 131.8 (C), 130.6 (C), 128.6 (2CH), 127.3 (C), 121.7 (CH), 118.6 (2CH), 112.5 (CH), 51.0 (CH$_2$Ph), 47.5 (*CH*-NH), 33.3 (2CH$_2$), 32.9 (CH$_2$), 25.3 (CH$_2$), 24.4 (CH$_2$). LC/MS *m/z* 378 [M+H]$^+$.

1-(4-((1H-benzo[d][1,2,3]triazol-1-yl)methyl)phenyl)-3-propylurea (101c)

Compound **101c** was obtained, in 39% total yield; mp: 165-167 °C; TLC (diethyl ether) R$_f$: 0.35. ^{1}H NMR (400 MHz, DMSO-*d*$_6$) δ: 8.45 (1H, s, *NH*-Ph), 8.03 (1H, d, *J* = 7.8, H-4), 7.83 (1H, d, *J* = 7.4, H-7), 7.52 (1H, t, H-5), 7.42 (1H, t, H-6), 7.34 (2H, d, *J* = 8.4, H-3′, 5′), 7.23 (2H, d, *J* = 8.4, H-2′, 6′), 6.13 (1H, t, *NH*-CH$_2$), 5.86 (2H, s, CH$_2$), 3.10-2.88 (2H, m, *CH*$_2$-NH), 1.48-1.35 (2H, q, *CH*$_2$CH$_3$), 0.84 (3H, t, CH$_2$*CH*$_3$). ^{13}C-NMR (DMSO-*d*$_6$) δ: 168.9 (CO), 145.5 (C), 132.8 (C), 131.8 (C), 135.5 (C), 129.2 (2CH), 126.2 (2CH), 121.5 (2CH), 119.6 (CH), 110.0 (CH), 54.7 (CH$_2$), 40.8 (CH$_2$), 22.9 (CH$_2$), 11.3 (CH$_3$). LC/MS *m/z* 310 [M+H]$^+$.

1-(4-((1H-benzo[d][1,2,3]triazol-1-yl)methyl)phenyl)-3-cyclohexylurea (102c)

Compound **102c** was obtained, in 66% total yield; mp: 141-143 °C; TLC (diethyl ether/ petroleum ether = 8/2) R$_f$: 0.28. ^{1}H NMR (400 MHz, DMSO-*d*$_6$) δ: 8.32 (1H, s, *NH*-Ph), 8.03 (1H, d, *J* = 8.4, H-4), 7.55 (1H, d, *J* = 8. H-7), 7.55-7.38 (2H, m, H-5,6), 7.32 (2H, d, *J* = 8.2, H-2′, 6′), 7.22 (2H, d, *J* = 8.4, H-3′, 5′), 6.06 (1H, d, *J* = 7.6, *NH*-CH), 5.86 (2H, s, CH$_2$), 3.51-3.25 (1H, m, CH), 1.80-1.40 (4H, m, 2CH$_2$), 1.35-1.00 (6H, m, 3CH$_2$). ^{13}C-NMR (DMSO-*d*$_6$) δ :168.9 (CO), 145.5 (C), 132.8 (C), 131.8 (C), 135.5 (C), 129.2 (2CH), 126.2 (2CH), 121.5 (2CH), 119.6 (CH), 110.0 (CH), 54.7 (CH$_2$), 47.5 (*CH*-NH), 33.3 (2CH$_2$), 32.9 (CH$_2$), 25.3 (CH$_2$), 24.4 (CH$_2$). LC/MS *m/z* 350 [M+H]$^+$.

1-(4-((1H-benzo[d][1,2,3]triazol-1-yl)methyl)phenyl)-3-phenylurea (103c)

Compound **103c** was obtained, in 16% total yield; mp: 159-161 °C; TLC (diethyl ether/ petroleum ether = 8/2) R$_f$: 0.31. ^{1}H NMR (400 MHz, DMSO-*d*$_6$) δ: 10.24 (1H, s, NH), 8.25 (1H, s, NH-Ph), 8.04 (1H, d, *J* = 8.2, H-4), 7.82 (1H, d, *J* = 8.2, H-7), 7.55 (2H, d, *J* = 8.6, H-3′, 5′), 7.48-7.38 (4H, m, arom), 7.32 (2H, d, *J* = 8.4, H-2′, 6′), 7.18-7.14 (1H, m, arom), 5.92 (2H, s, CH$_2$). ^{13}C-NMR (DMSO-*d*$_6$) δ168.9 (CO), 145.5 (C), 139.4 (C), 132.8 (C), 131.8 (C), 135.5 (C), 129.2 (2CH), 128.9 (2CH), 126.2 (2CH), 126.10 (CH), 121.8 (2CH), 121.5 (2CH), 119.6 (CH), 110.0 (CH), 54.7 (CH$_2$). LC/MS *m/z* 344 [M+H]$^+$.

1-(4-((5,6-difluoro-1H-benzo[d][1,2,3]triazol-1-yl)methyl)phenyl)-3-ethylurea (104d)

Compound **104d** was obtained, in 47% total yield; mp: 286-287 °C; TLC (petroleum ether/ethyl acetate = 6/4) R$_f$: 0.11. ^{1}H NMR (400 MHz, DMSO-*d*$_6$) δ: 8.46 (H, s, *NH*-Ph), 8.21 (H, t, H-4), 8.10 (H, t, H-7), 7.36 (2H, d, *J* = 8.4 Hz, H-3′,5′), 7.27 (2H, d, *J* = 8.4 Hz, H-2′,6′), 6.08 (H, t, *NH*-CH$_2$), 5.83 (2H, s, *CH*$_2$-Ph), 3.11-3.04 (2H, m, CH$_2$), 1.03 (3H, t, CH$_3$). ^{13}C-NMR (DMSO-*d*$_6$) δ: 151.6 (CO), 149.1 (2CF), 140.6 (C), 140.5 (C), 128.7 (C), 128.6 (2CH), 128.5 (C), 118.4 (2CH), 106.0 (CH), 98.5 (CH), 51.0 (CH$_2$-Ph), 33.9 (CH$_2$), 15.4 (CH$_3$). LC/MS *m/z* 332 [M+H]$^+$.

1-(4-((5,6-difluoro-1H-benzo[d][1,2,3]triazol-1-yl)methyl)phenyl)-3-propylurea(105d)

Compound **105d** was obtained, in 41% total yield; mp: 286-287 °C; TLC (petroleum ether/ethyl acetate = 6/4) R$_f$: 0.17. ^{1}H NMR (400 MHz, DMSO-*d*$_6$) δ: 8.45 (H, s, *NH*-Ph), 8.21 (H, t, H-4), 8.10 (H, t, H-7), 7.35 (2H, d, *J* = 8.8 Hz, H-3′,5′), 7.30 (2H, d, *J* = 8.8 Hz, H-2′,6′), 6.12 (H, t, *NH*-CH$_2$), 5.83 (2H, s, CH$_2$), 3.00 (2H, q, *CH*$_2$-NH), 1.41 (2H, q, *CH*$_2$-CH$_3$), 0.84 (3H, t, CH$_3$). ^{13}C-NMR (DMSO-*d*$_6$) δ: 152.3 (CO), 149.2 (2CF), 140.6 (2C), 128.7 (2CH), 127.5 (2C), 117.7 (2CH), 106.1 (CH), 98.4 (CH), 51.0 (CH$_2$), 40.8 (CH$_2$), 22.9 (CH$_2$), 11.3 (CH$_3$). LC/MS *m/z* 345 [M+H]$^+$.

1-butyl-3-(4-((5,6-difluoro-1H-benzo[d][1,2,3]triazol-1-yl)methyl)phenyl)urea (106d)

Compound **106d** was obtained, in 18% total yield; mp: 193-194 °C; TLC (petroleum ether/ethyl acetate = 6/4) R$_f$: 0.26. ^{1}H NMR (400 MHz, DMSO-*d*$_6$) δ: 8.44 (H, s, *NH*-Ph), 8.21 (H, t, H-4), 8.10 (H, t, H-7), 7.35 (2H, d, *J* = 8.4 Hz, H-3′,5′), 7.26 (2H, d, *J* = 8.4 Hz, H-2′,6′), 6.10 (H, t, *NH*-CH$_2$), 5.83 (2H, s, CH$_2$), 3.05 (2H, t, *CH*$_2$-NH), 1.38 (2H, q, CH$_2$-*CH*$_2$), 1.33-1.23 (2H, m, *CH*$_2$-CH$_2$-CH$_3$), 0.88 (3H, t, CH$_3$). ^{13}C-NMR (DMSO-*d*$_6$) δ: 155.0 (CO), 149.2 (2CF), 140.6 (2C), 128.7 (2CH), 127.5 (2C), 177.6 (2CH), 106.1 (CH), 98.5 (CH), 51.0 (CH$_2$-Ph), 32.1 (CH$_2$), 31.8 (CH$_2$), 19.5 (CH$_2$), 13.7 (CH$_3$). LC/MS *m/z* 360 [M+H]$^+$.

1-cyclohexyl-3-(4-((5,6-difluoro-1H-benzo[d][1,2,3]triazol-1-yl)methyl)phenyl)urea (107d)

Compound **107d** was obtained, in 16% total yield; mp: 203-204 °C; TLC (petroleum ether/ethyl acetate = 6/4) R$_f$: 0.32. ^{1}H NMR (400 MHz, DMSO-*d*$_6$) δ: 8.36 (H, s, *NH*-Ph), 8.21 (H, t, H-4), 8.10 (H, t, H-7), 7.34 (2H, d, *J* = 8,8 Hz, H-

3′,5′), 7.26 (2H, d, *J* = 8,8 Hz, H-2′,6′), 6.08 (H, d, *NH*-CH), 5.80 (2H, s, CH_2), 3.44-3.40 (H, m, *CH*-NH), 1.82-1.45 (6H, m, $3CH_2$), 1.30-0.98 (4H, m, $2CH_2$). ^{13}C-NMR (DMSO-d_6) δ: 156.6 (CO), 154.2 (C), 149.2 (2CF), 140.6 (C), 140.4 (C), 128.7 (2CH), 128.6 (C), 117.6 (2CH), 106.1 (CH), 98.5 (CH), 51.0 (CH_2), 47.5 (CH), 33.3 ($2CH_2$), 32.9 (CH_2), 25.2 (CH_2), 24.3 (CH_2). LC/MS *m*/*z* 386 $[M+H]^+$.

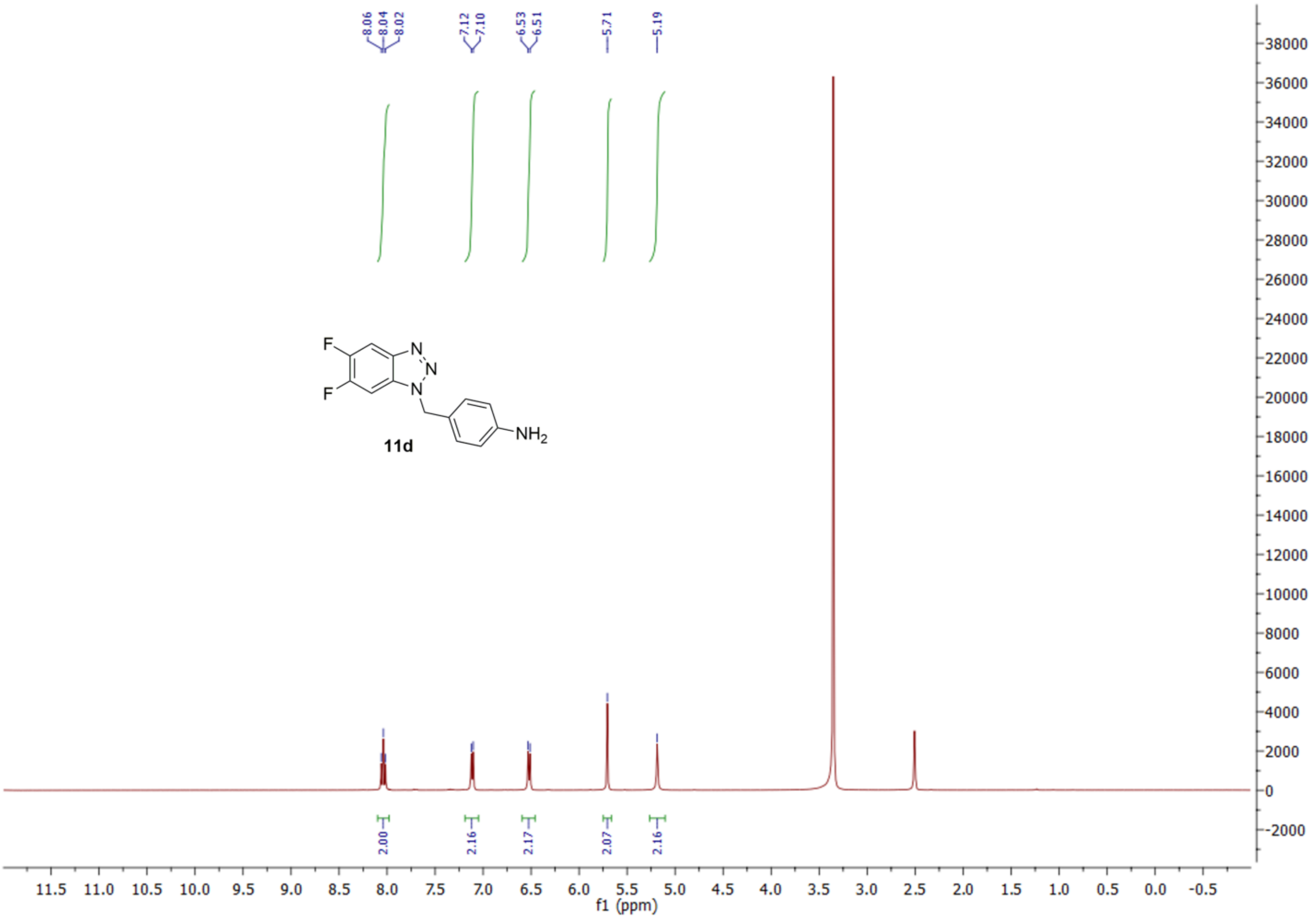

Figure S2. ^{1}H-NMR for compound **11d**.

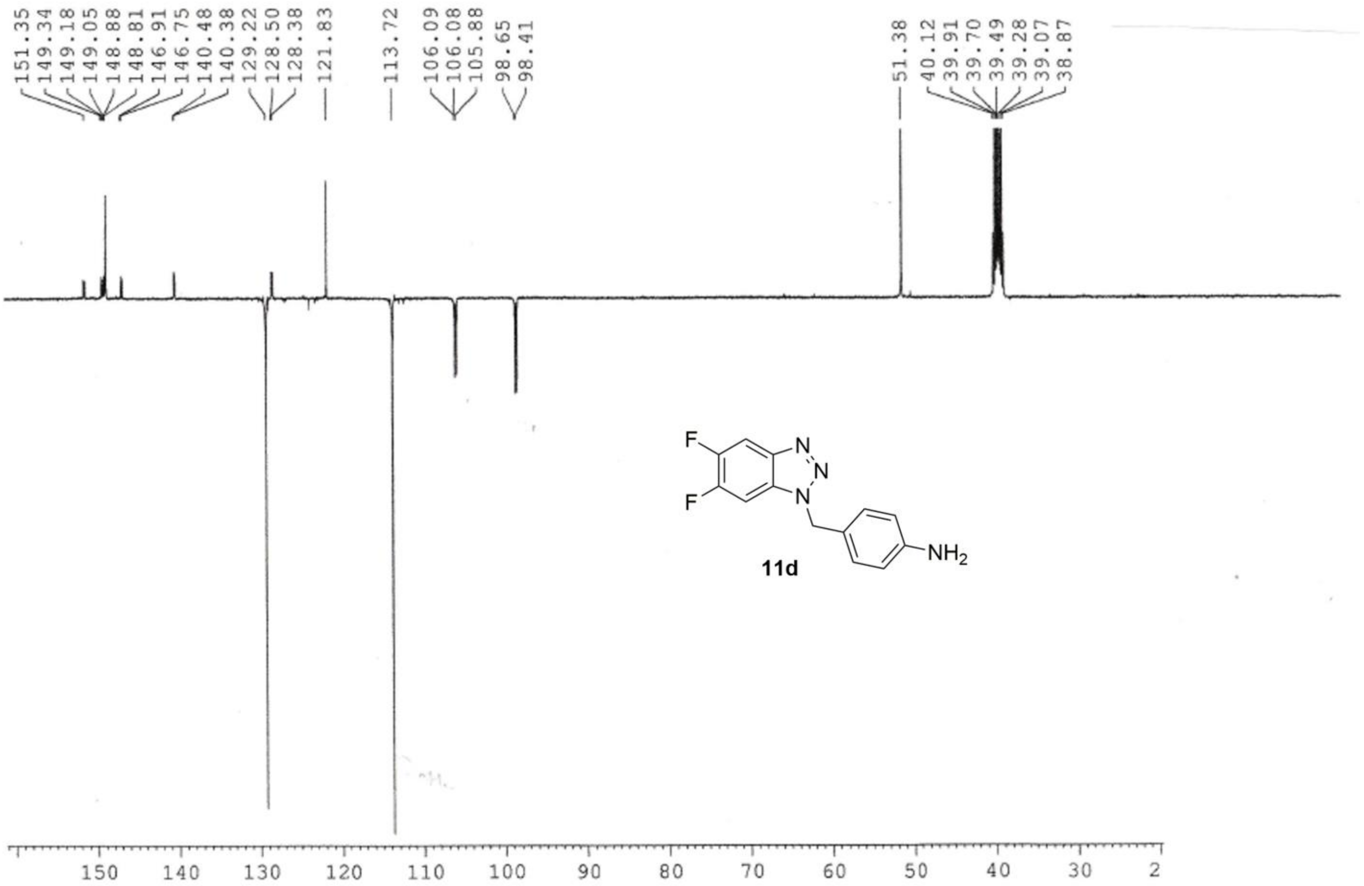

Figure S3. ^{13}C-NMR for compound **11d**.

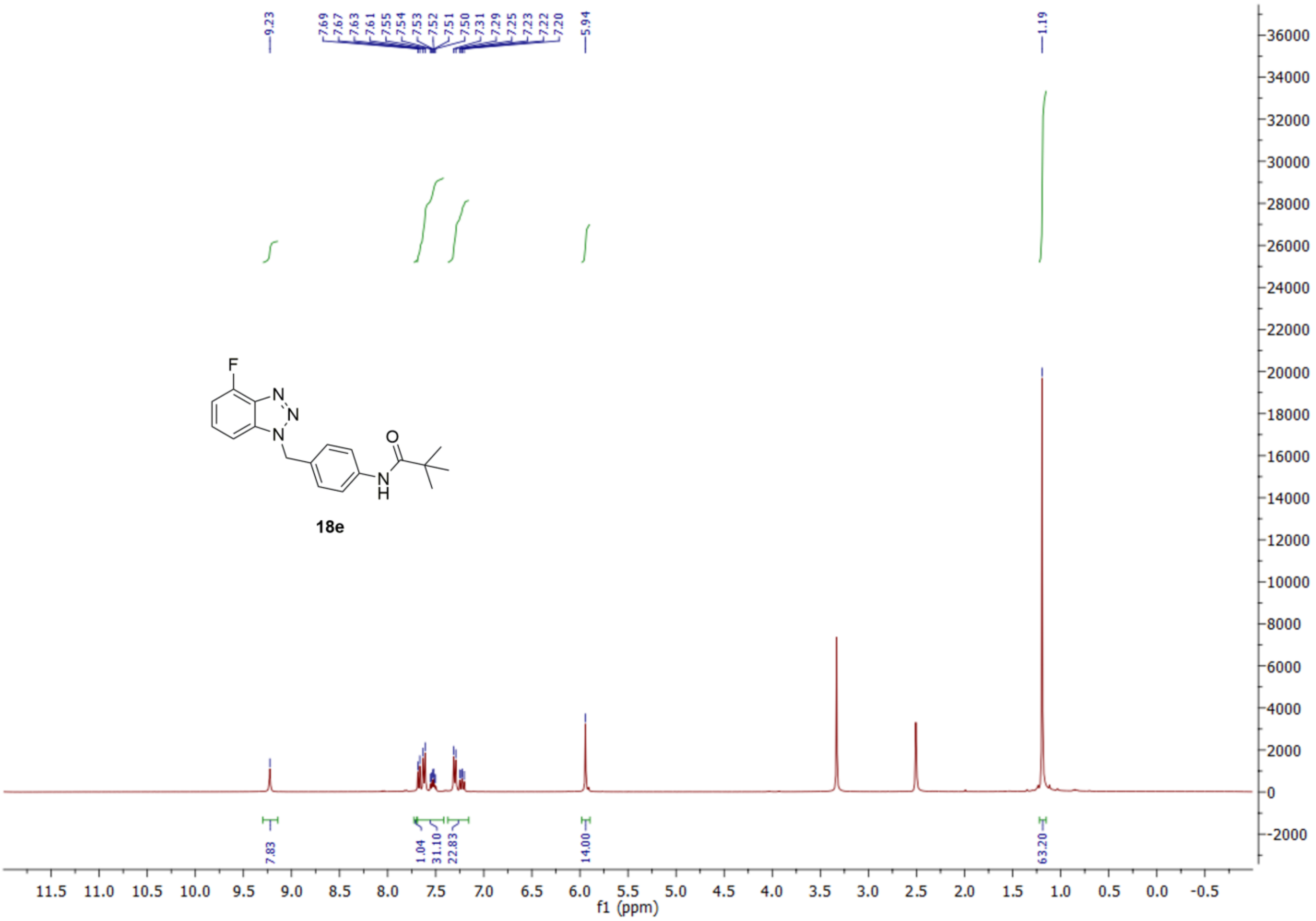

Figure S4. ^{1}H-NMR for compound 18e.

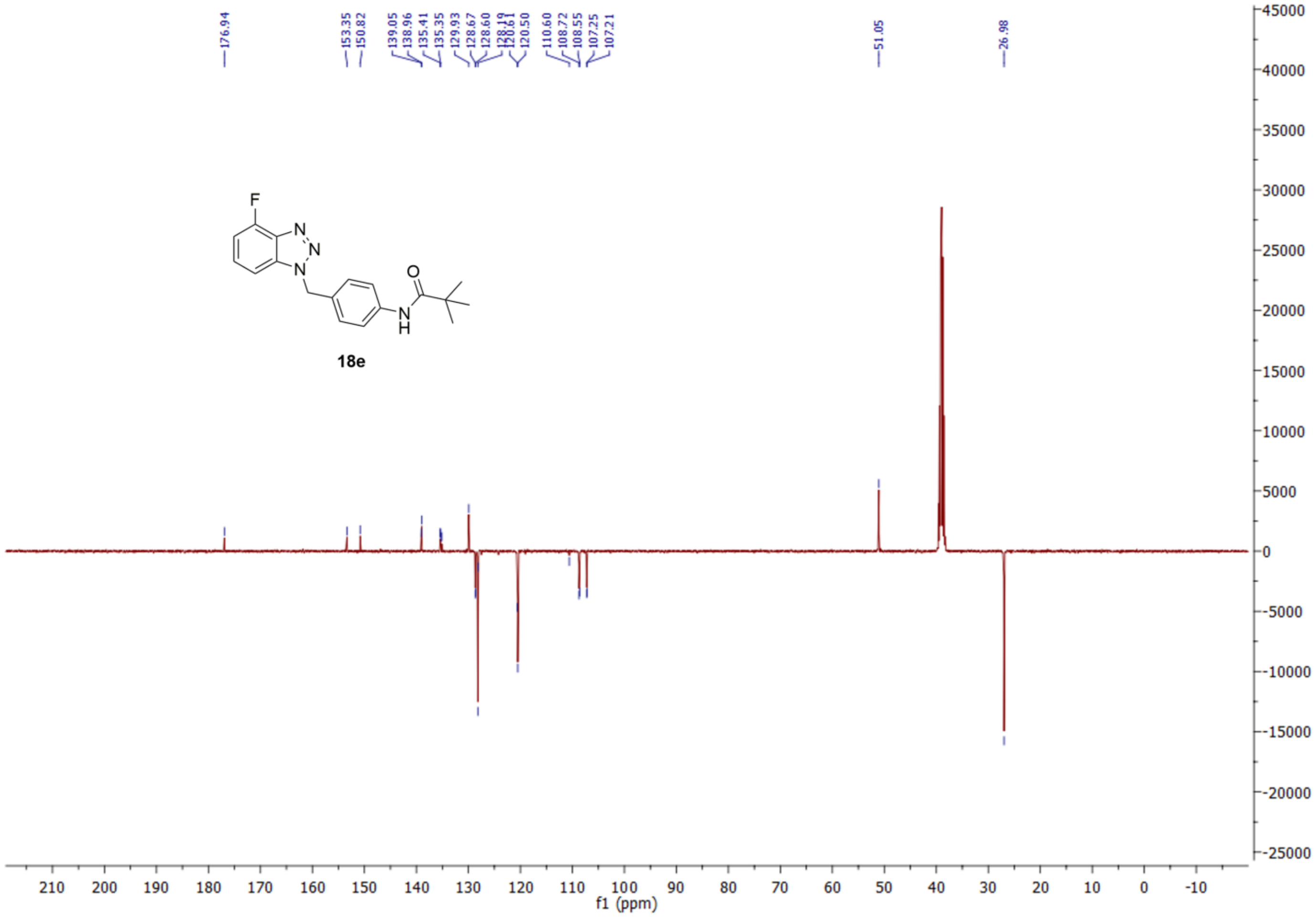

Figure S5. ^{13}C-NMR for compound **18e**.

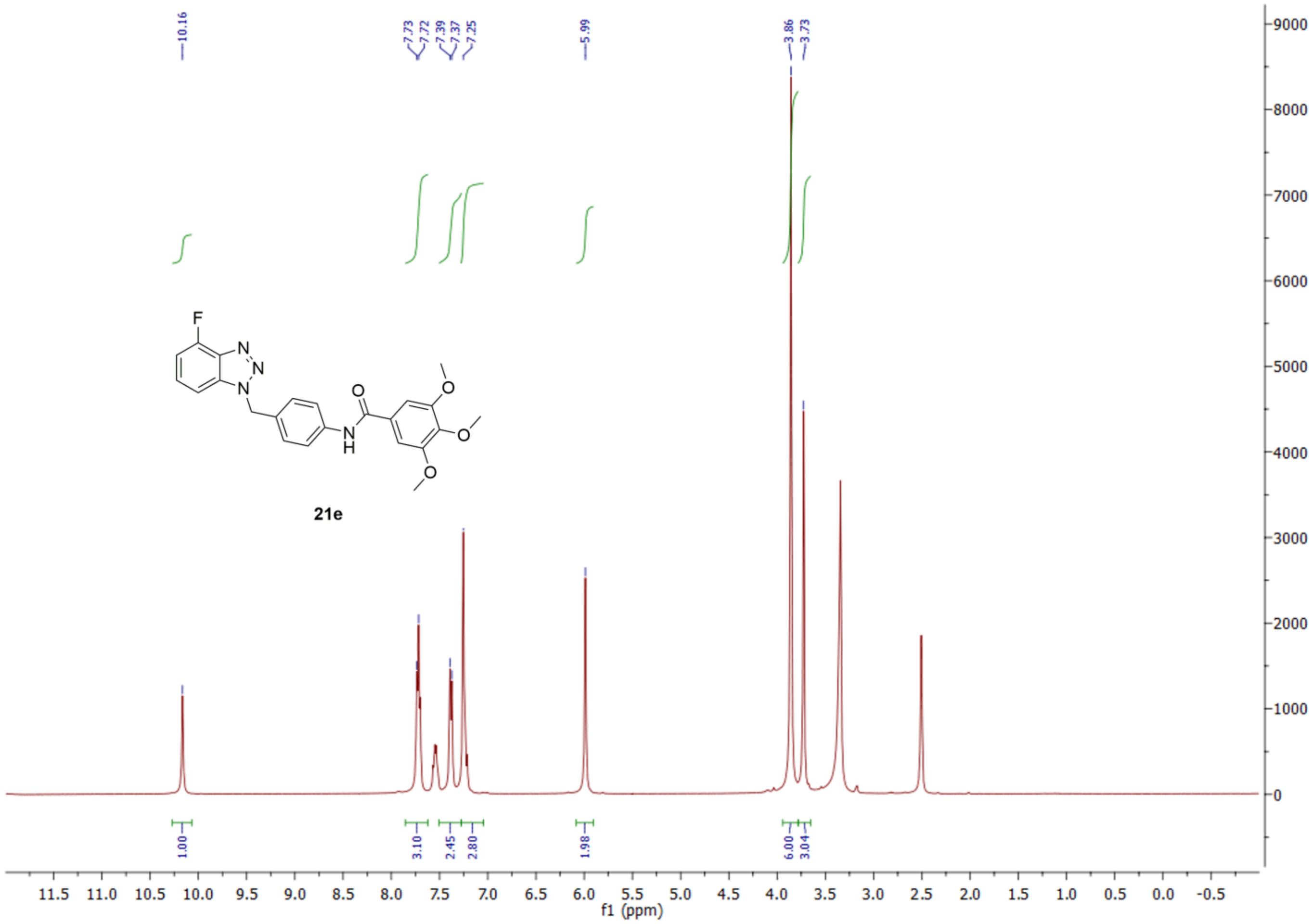

Figure S6. ^{1}H-NMR for compound **21e**.

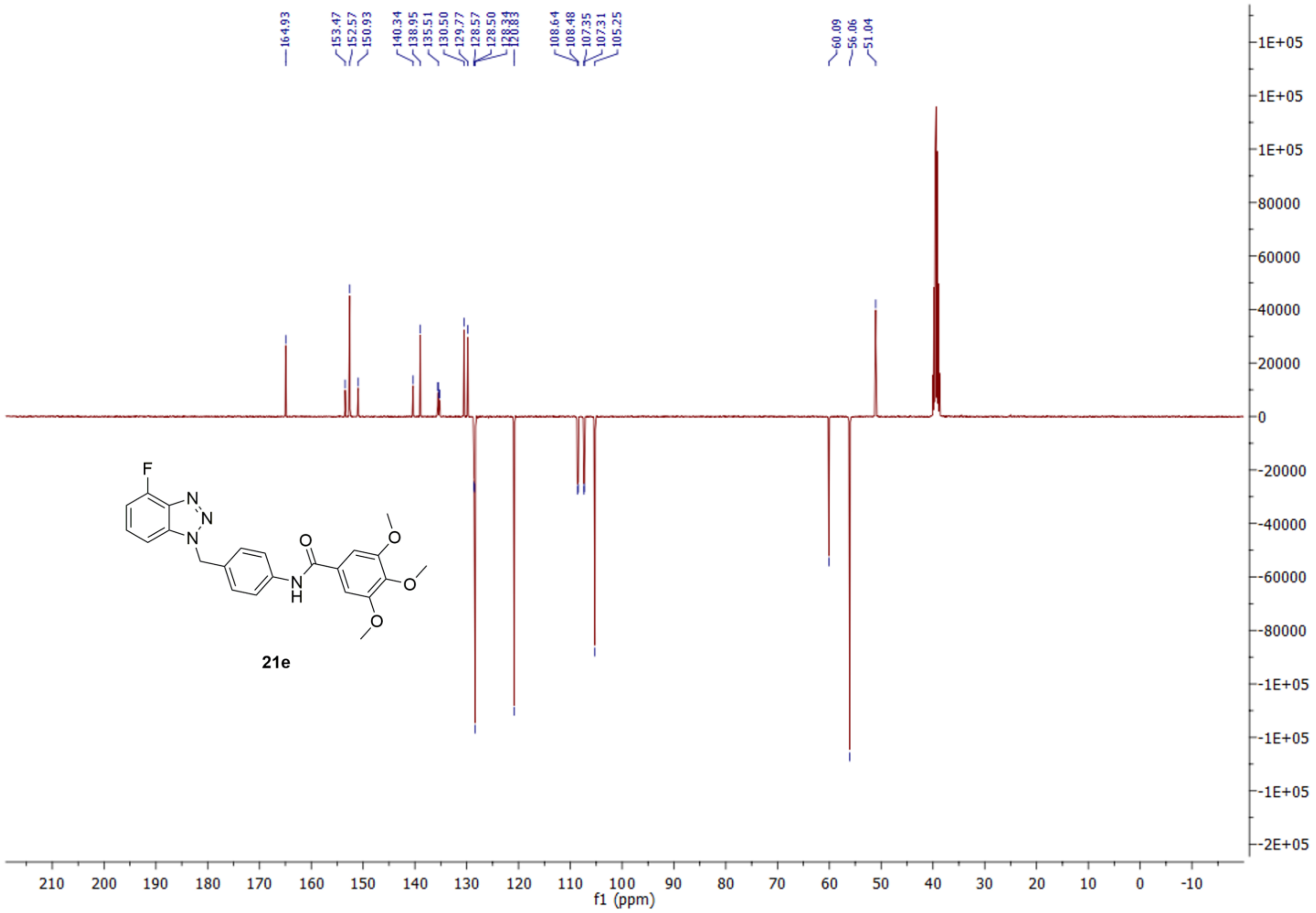

Figure S7. ^{13}C-NMR for compound **21e**.

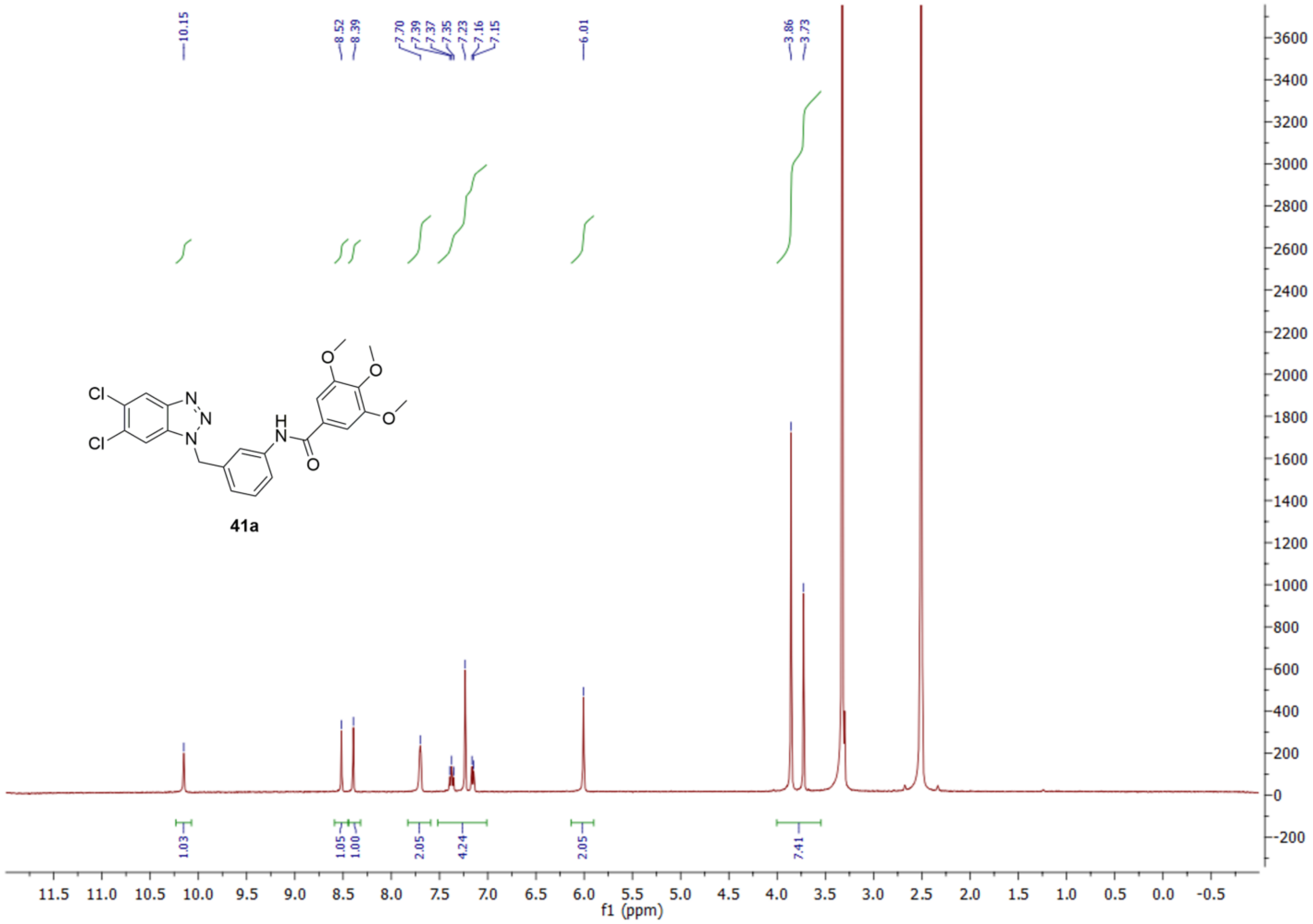

Figure S8. ^{1}H-NMR for compound **41a**.

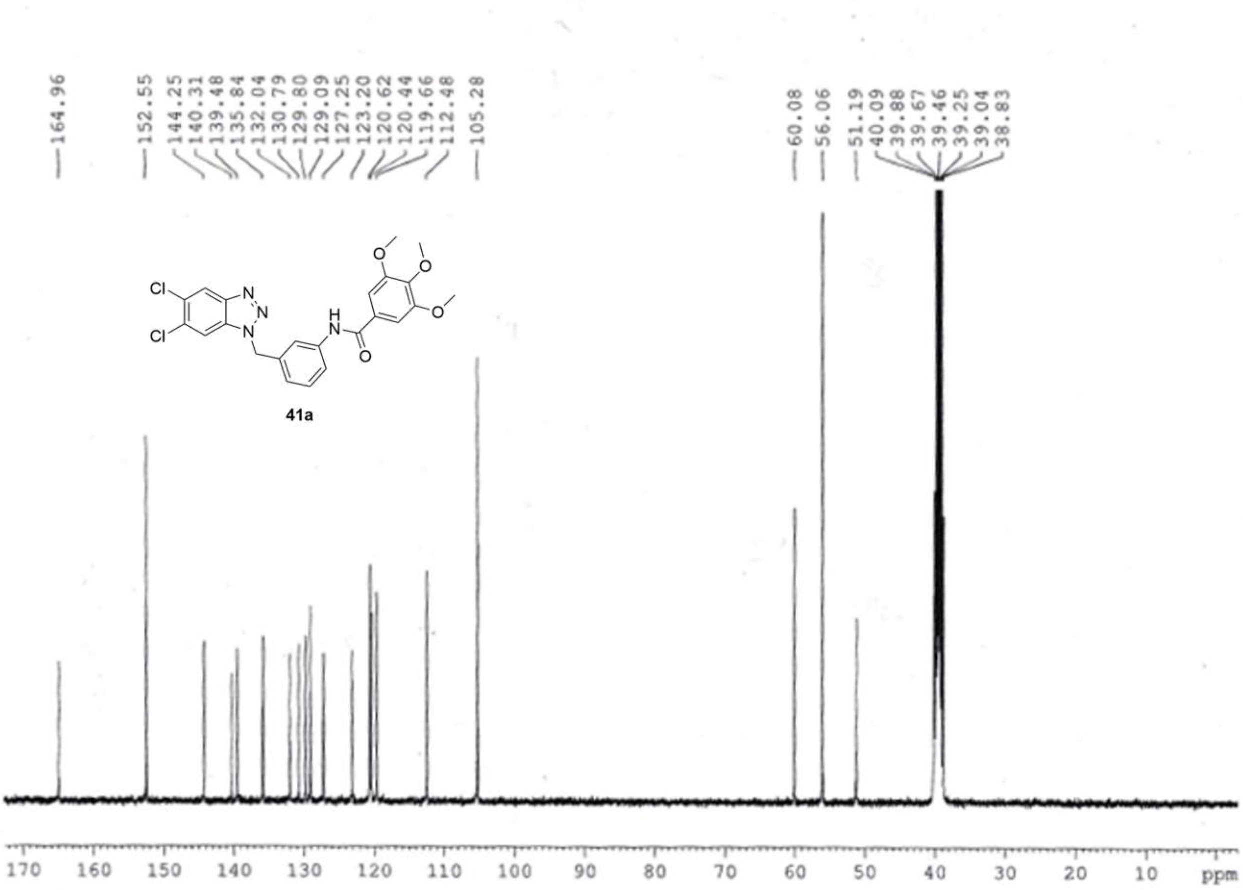

Figure S9. ^{13}C-NMR for compound **41a**.

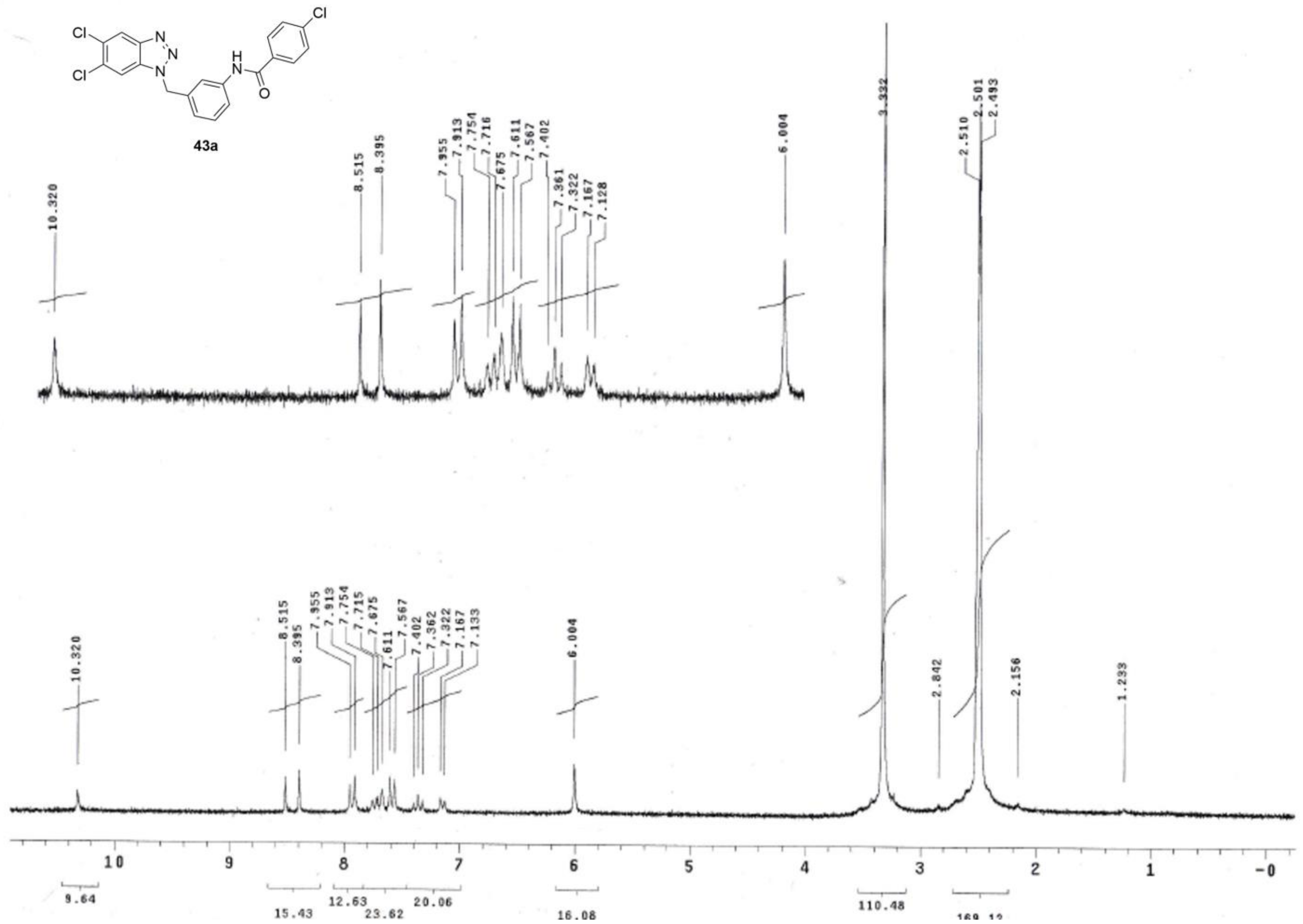

Figure S10. ^{1}H-NMR for compound **43a**.

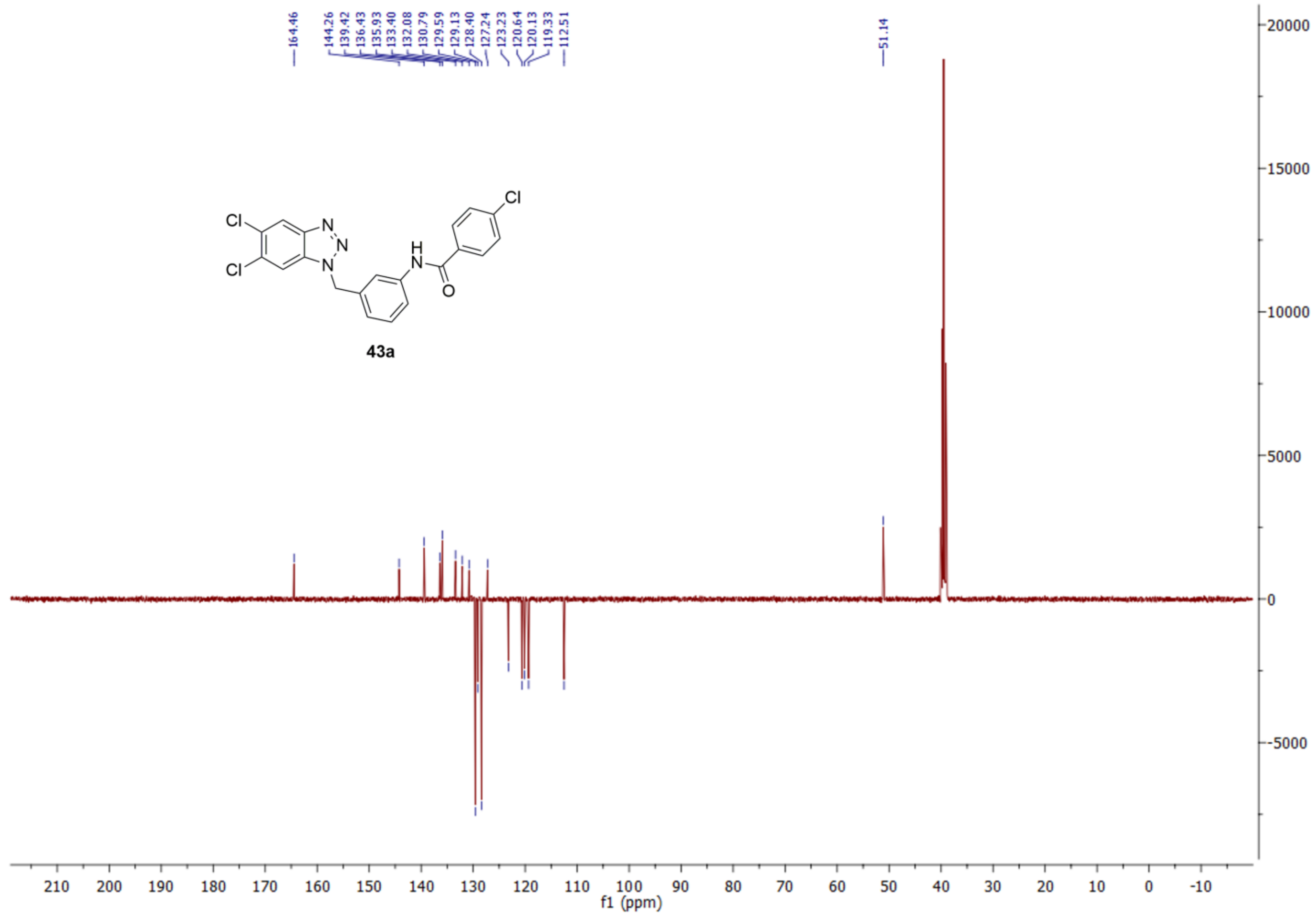

Figure S11. ^{13}C-NMR for compound **43a**.

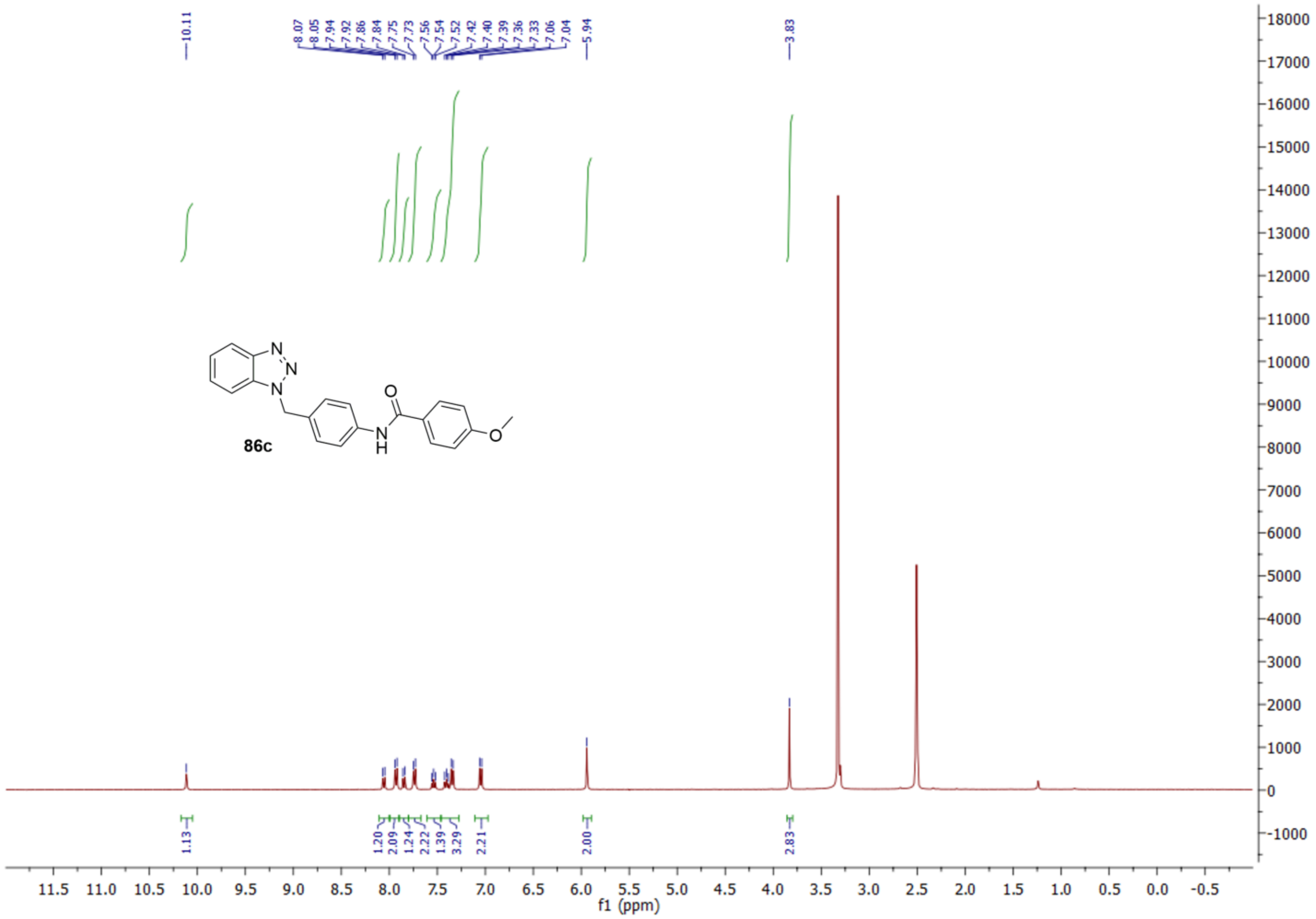

Figure S12. ^{1}H-NMR for compound **86c**.
